# Effects of different recovery and regeneration modalities on delayed-onset muscle soreness and post-exercise muscle damage markers: a systematic review and network meta-analysis

**DOI:** 10.3389/fbioe.2026.1956053

**Published:** 2026-10-26

**Authors:** Jianxin Li, Siyu Wang, Ming Wei, Weipeng Liu

**Affiliations:** School of Strength and Conditioning, Shenyang Sport University, Shenyang, China

**Keywords:** creatine kinase, delayed-onset muscle soreness, exercise-induced muscle damage, interleukin-6, network meta-analysis, post-exercise recovery

## Abstract

**Objective:**

This study employed a systematic review and Bayesian network meta-analysis to compare the effects of multiple post-exercise interventions during the early recovery period, examine whether these effects varied across recovery time points, and provide evidence to inform the selection of post-exercise recovery strategies.

**Methods:**

PubMed, Embase, the Cochrane Library, and Web of Science were systematically searched, and randomized controlled trials, including parallel-group and crossover designs, were included. A Bayesian random-effects network meta-analysis was used to estimate mean differences (MDs) and 95% credible intervals (CrIs). The revised Cochrane risk-of-bias tool for randomized trials (RoB 2) and the Confidence in Network Meta-Analysis (CINeMA) framework were used to assess risk of bias and confidence in the evidence, respectively. Sensitivity analyses and exploratory univariable network meta-regression analyses were also performed.

**Results:**

A total of 35 studies were included in this review. For delayed-onset muscle soreness (DOMS), whole-body cryotherapy (WBC) and photobiomodulation/phototherapy-related interventions (PHO) showed relatively large reductions in soreness at both 24 and 48 h after exercise. Compared with the combined control/placebo condition (CON/PBO), the effect estimates for WBC were MD = −38.92 (95% CrI: −69.08 to −9.15) at 24 h and MD = −40.03 (95% CrI: −67.74 to −12.44) at 48 h. The corresponding estimates for PHO were MD = −18.20 (95% CrI: −32.91 to −3.72) and MD = −19.46 (95% CrI: −33.55 to −5.99), respectively. For creatine kinase (CK), low-level laser therapy (LLLT) and PHO were associated with lower CK levels at both 24 and 48 h, with the largest estimated effect for LLLT at 48 h (MD = −499.96, 95% CrI: −831.59 to −203.59). For interleukin-6 (IL-6), CWI showed favorable estimates at both 24 and 48 h, although the evidence base was limited and the network was sparse.

**Conclusion:**

WBC and PHO showed relatively consistent favorable effects on DOMS at 24 and 48 h, whereas LLLT and PHO showed relatively consistent associations with lower CK levels. The IL-6 findings should be interpreted as exploratory because of the limited evidence base. Selection of post-exercise recovery strategies should consider the target outcome, recovery time window, evidence certainty, and network structure.

## 1 Introduction

Delayed-onset muscle soreness (DOMS) is a common post-exercise recovery concern following high-intensity, eccentric, or unaccustomed exercise. It typically develops gradually over the hours following exercise and may become most pronounced between 24 and 72 h (Lewis et al., 2012). DOMS is not attributable to a single factor and may instead involve multiple processes, including microscopic sarcomeric damage, impaired excitation–contraction coupling, altered cell membrane permeability, disrupted calcium homeostasis, release of inflammatory mediators, and nociceptive sensitization (Clarkson and Hubal, 2002; Nosaka et al., 2002; Proske and Allen, 2005; Peake et al., 2017). In exercise training and competitive sport, DOMS manifests not only as localized pain, tenderness, and stiffness but may also be accompanied by reduced muscle strength, restricted joint range of motion, and impaired exercise performance, thereby affecting training continuity, recovery between competitions, and subsequent load management.

Reliance on subjective soreness ratings alone cannot fully characterize post-exercise muscle damage or the recovery process. Creatine kinase (CK) is commonly used as an indirect biochemical marker of altered skeletal muscle cell membrane permeability and muscle-damage-related biochemical responses, whereas interleukin-6 (IL-6) is associated with exercise-induced inflammation, immune regulation, and the endocrine functions of skeletal muscle (Peake et al., 2017; Pedersen and Febbraio, 2008). Assessing DOMS together with CK and IL-6 therefore allows recovery interventions to be evaluated across multiple dimensions, including subjective soreness, muscle-damage-related biochemical responses, and inflammatory responses.

Interventions currently used for post-exercise recovery include cold-water immersion, contrast-water therapy, local cryotherapy, whole-body cryotherapy, compression therapy, kinesiology taping, vibration therapy, electrical stimulation/electrotherapy, stretching, massage/manual therapy, acupuncture, photobiomodulation/low-level laser therapy, shock wave therapy, and low-intensity focused ultrasound, among others. Previous studies and reviews suggest that different recovery strategies may have differing effects on DOMS, CK, inflammatory responses, or fatigue recovery (Dupuy et al., 2018; Higgins et al., 2017; Costello et al., 2015; Hill et al., 2014; Guo et al., 2017; Herbert and De Noronha, 2007). However, traditional systematic reviews have mostly focused on a single intervention or a small number of interventions, and considerable variation exists across studies in participants' training status, exercise-induced damage models, intervention parameters, intervention sites, outcome measurement methods, and follow-up time points, leaving the relative merits of multiple recovery strategies unclear.

Network meta-analysis (NMA) can integrate direct and indirect comparative evidence within a single model, enabling simultaneous comparisons of multiple interventions (Higgins et al., 2012; Salanti et al., 2011; Owen et al., 2019). The present evidence base included numerous recovery strategies, but head-to-head comparisons between active interventions were uncommon. Most networks were connected through a shared control condition. Pairwise meta-analysis alone could therefore not estimate all clinically relevant contrasts. We selected a Bayesian random-effects NMA to synthesize the connected evidence, account for between-study heterogeneity, and quantify uncertainty using posterior distributions and 95% credible intervals (CrIs). Ranking probabilities summarized the relative ordering of interventions but were considered supplementary to effect estimates, precision, evidence structure, and certainty. Within this framework, laser-only LLLT and LED-containing photobiomodulation regimens (PHO) were considered separate treatment categories to compare the light-based configurations represented in the included studies.

## 2 Methods

### 2.1 Registration

This systematic review and network meta-analysis followed the Preferred Reporting Items for Systematic Reviews and Meta-Analyses (PRISMA) 2020 statement and the PRISMA extension for network meta-analyses (PRISMA-NMA) (Page et al., 2021; Hutton et al., 2015). The protocol was registered with PROSPERO (registration number CRD420261397771).

### 2.2 Search strategy

PubMed, Embase, the Cochrane Library, and Web of Science were systematically searched from database inception to May 2026. Search terms were constructed according to the population, intervention, comparator, outcomes, and study design (PICOS) framework, covering healthy adults or exercising populations, exercise-induced DOMS, post-exercise recovery interventions, DOMS/CK/IL-6, and randomized trial designs. No restrictions were placed on year of publication or language. The complete search strategy is provided in Supplementary Table 1.

### 2.3 Inclusion and exclusion criteria

Inclusion criteria were as follows: (1) participants were healthy adults, physically active individuals, university students, or amateur or competitive athletes; (2) interventions were post-exercise recovery or regeneration interventions, including cold-water immersion, contrast-water therapy, local cryotherapy, whole-body cryotherapy, compression therapy, kinesiology taping, vibration therapy, electrical stimulation/electrotherapy, stretching, massage/manual therapy, acupuncture, photobiomodulation/low-level laser therapy, and other clearly classifiable recovery interventions; (3) comparators were passive recovery, usual-care recovery, no treatment, placebo/sham intervention, or other eligible active recovery interventions; (4) outcomes included at least one of DOMS, CK, or IL-6, with data reported at 24 or 48 h after exercise; and (5) eligible study designs were randomized controlled trials, including parallel-group and crossover designs.

The exclusion criteria were as follows: non-randomized controlled studies, animal experiments, reviews, case reports, conference abstracts, studies for which the full text could not be obtained, studies that did not use a delayed-onset muscle soreness or exercise-induced muscle damage model, studies in which the intervention could not be clearly classified, and studies for which 24 h or 48 h outcome data could not be extracted or converted.

For intervention-node classification, LLLT was defined as laser-only low-level laser therapy; the included protocols used 810-nm laser diodes without an LED component. PHO was defined operationally as LED-containing photobiomodulation regimens, encompassing diode-based phototherapy and combined laser-LED protocols, some of which also incorporated a static magnetic field. Laser-only protocols were assigned to LLLT, and LED-containing protocols were assigned to PHO. These mutually exclusive operational categories distinguished the delivered treatment configurations within the broader photobiomodulation family. Their separate representation enabled comparison of the two categories while retaining the broader scope of PHO. Study-specific light sources and additional components are detailed in Supplementary Table 3A.

### 2.4 Study selection and data extraction

After search records were imported into EndNote and duplicates were removed, two reviewers independently screened titles and abstracts and subsequently assessed the full texts. The screening process was assisted by the ASReview active-learning tool for prioritization, but final inclusion decisions were made by the reviewers according to the prespecified criteria (Van De Schoot et al., 2021). Disagreements were resolved through discussion and, when necessary, adjudication by a third reviewer.

Two reviewers independently extracted the data and cross-checked the extracted information. Extracted variables included first author, publication year, sample size, age, sex, study population, study design, exercise-induced damage model, intervention, comparator, intervention site, outcomes, measurement time points, means, standard deviations, and group sample sizes. DOMS data were converted to a 0–100 mm visual analog scale (VAS); CK data were converted to U/L; and IL-6 data were converted to pg/mL wherever possible. When a study reported only standard errors, confidence intervals, quartiles, or graphical data, values were converted using methods recommended in the Cochrane Handbook or extracted with an image-digitization tool. For multi-arm studies, all eligible intervention and comparator arms were retained, and correlations among arms arising from shared controls were accounted for in the model.

For randomized crossover trials, the primary NMA used the condition-specific means, standard deviations, and sample sizes reported for each intervention period as marginal arm-level summaries. Paired-difference standard deviations and within-participant correlation coefficients were unavailable in the extracted dataset for the required outcome and time-point combinations. Consequently, no correlation coefficient was imputed, and the arm-based MetaInsight model did not explicitly account for within-participant covariance. Because paired analyses are preferable for crossover data when the required information is available (Elbourne et al., 2002), we conducted a design-specific sensitivity analysis that excluded randomized crossover trials. The crossover-specific risk-of-bias assessment separately considered intervention-sequence randomization, washout, period effects, and carryover effects (Supplementary Table 5). No additional adjustment for period or carryover effects was applied because the required study-level information was not consistently available.

### 2.5 Outcomes

The primary outcome was DOMS, assessed mainly using the VAS. The secondary outcome was CK, used as an indirect biochemical marker of muscle-damage-related responses after exercise. The exploratory outcome was IL-6, used to characterize post-exercise inflammatory and immune responses.

Measurement time points in the included studies were concentrated mainly within the early post-exercise recovery period. DOMS typically develops gradually from several hours after exercise to the following day. Accordingly, 24 h reflects the early stage of symptom emergence, whereas 48 h more closely represents the period when soreness and functional discomfort are often more pronounced (Cheung et al., 2003; Mizumura and Taguchi, 2016). CK and IL-6 also exhibit clear time-dependent changes. CK reflects alterations in muscle-cell membrane permeability and biochemical responses related to muscle damage, whereas IL-6 reflects post-exercise inflammatory and muscle-derived cytokine responses (Kuipers, 1994; Bruunsgaard et al., 1997). Previous systematic reviews of indirect markers of exercise-induced muscle damage have similarly treated 24 and 48 h as important time points for assessing early damage and recovery (Dupuy et al., 2018). Therefore, this study extracted outcome data at 24 and 48 h after exercise and constructed separate network meta-analysis models for each time point. For all three outcomes, lower values were coded as favoring the intervention in the statistical analyses. However, reductions in IL-6 were interpreted cautiously because IL-6 has complex physiological roles in exercise and recovery.

### 2.6 Risk-of-bias assessment of included studies

Two reviewers independently assessed risk of bias using the revised Cochrane risk-of-bias tool for randomized trials (RoB 2) (Sterne et al., 2019). Disagreements were resolved through discussion or adjudication by a third reviewer. For parallel-group randomized controlled trials, we assessed the randomization process, deviations from intended interventions, missing outcome data, outcome measurement, and selective reporting. For randomized crossover trials, we also considered intervention sequence, washout period, period effects, and carryover effects. Confidence in the network estimates was assessed using the Confidence in Network Meta-Analysis (CINeMA) framework across six domains: within-study bias, reporting bias, indirectness, imprecision, heterogeneity, and incoherence (Nikolakopoulou et al., 2020). Certainty was also structured according to Grading of Recommendations Assessment, Development and Evaluation (GRADE) guidance for NMA by considering direct, indirect, and network evidence separately (Puhan et al., 2014; Brignardello-Petersen et al., 2018). When closed loops were limited or formal local inconsistency testing was infeasible, interpretation integrated the available evidence sources, network structure, interval estimates, heterogeneity, and confidence assessments.

### 2.7 Statistical analysis

Network plots presented the evidence structure, with node size representing the number of participants assigned to each intervention and edge thickness representing the amount of direct evidence (Salanti et al., 2011). We assessed transitivity by comparing selected potential effect modifiers across direct treatment comparisons. These modifiers included the exercise-induced muscle damage model, intervention or body region, participant training status, and study design. They were selected because each could plausibly influence post-exercise muscle damage and recovery responses. Comparison-level distributions are presented in Supplementary Table 36. We conducted Bayesian random-effects NMA for continuous outcomes using mean differences (MDs) and 95% CrIs, with CON/PBO as the reference treatment. Analyses were implemented in MetaInsight (v7.1.1) (Owen et al., 2019) using Markov chain Monte Carlo (MCMC) simulation (Van Valkenhoef et al., 2012). Four MCMC chains were run with 5,000 burn-in iterations, followed by 20,000 sampling iterations per chain and a thinning interval of 1. This procedure yielded 80,000 retained posterior samples. The random seed was 74. Diffuse normal priors [N (0, 342,225), parameterized in MetaInsight as mean and variance] were assigned to relative treatment effects and study-specific intercepts. A Uniform (0, 39) prior was assigned to the between-study heterogeneity standard deviation. Convergence was assessed using trace plots, Brooks–Gelman–Rubin diagnostics, and posterior density plots (Brooks and Gelman, 1998). Model fit was evaluated using posterior mean deviance (Dbar), the effective number of parameters (pD), deviance information criterion (DIC), and between-study heterogeneity (Spiegelhalter et al., 2002). Supplementary Tables 31-33 provide the model specifications and convergence diagnostics.

Global inconsistency was assessed by comparing the random-effects consistency model with the unrelated mean effects (UME) inconsistency model. Comparisons used Dbar, pD, DIC, and arm-level residual-deviance plots (Dias et al., 2013). Local inconsistency was assessed by node-splitting when treatment contrasts had independent direct and indirect evidence. A value of P < 0.05 was considered potential evidence of local inconsistency (Dias et al., 2013). When node-splitting was not estimable because the required evidence was unavailable, this limitation was reported explicitly. Supplementary Tables 34, 35 and Supplementary Figure 4 provide the global and local inconsistency results. Intervention rankings were summarized using the surface under the cumulative ranking curve (SUCRA) (Salanti et al., 2011). Rankings were considered supplementary to effect estimates, 95% CrIs, and confidence in the evidence.

Passive, no-treatment, usual-recovery, and placebo/sham controls were pooled as CON/PBO to provide a common reference for comparing recovery interventions, given limited evidence for individual control subtypes within several treatment comparisons. Similar grouping has been used in previous NMAs (Gianola et al., 2022; Recchia et al., 2022). Sensitivity to this classification was assessed by separating CON and PBO_SHAM for DOMS and CK at 24 and 48 h using the same Bayesian framework, excluding studies with unclear controls. Control separation disconnected the IL-6 networks, precluding corresponding analyses (Supplementary Section 11; Supplementary Tables 37-39).

Sensitivity analyses involved re-estimating the model after excluding, in turn, studies with potentially extreme effect sizes, small studies, and randomized crossover trials, and comparing the effect direction, 95% CrIs, intervention rankings, and retention of key nodes. If an intervention node disappeared from the network after exclusion of a given study, effect comparisons for that node were not performed; the disappearance of the node was instead interpreted as indicating complete dependence of that node on the excluded study. Primary Bayesian between-study heterogeneity was summarized using the posterior between-study standard deviation (τ) from the random-effects model (Supplementary Table 32). Separately, descriptive network-level heterogeneity statistics (τ^2^, τ, and I^2^) and prediction intervals for each intervention versus CON/PBO are reported in Supplementary Section 7 and Supplementary Tables 20-26. These supplementary quantities are distinct from the posterior τ estimates of the primary Bayesian models and were treated as descriptive uncertainty summaries rather than as evidence overriding the primary Bayesian 95% CrIs, certainty assessments, or conclusions.

For the crossover-design sensitivity analysis, all randomized crossover trials were excluded before the DOMS and CK networks were re-estimated. This analysis assessed whether the principal findings depended on crossover evidence whose within-participant covariance was not explicitly incorporated in the primary arm-based model. Details are provided in Supplementary Section 6.5 and Supplementary Table 19B.

We used exploratory univariable network meta-regression to examine whether study-level characteristics influenced the network estimates. Each random-effects shared-β model included one study-level covariate (Dias et al., 2013). Covariates included the exercise-induced damage model, intervention or body region, training status, study design, overall risk of bias, and small-sample status. Findings were interpreted jointly using β estimates, 95% CrIs, model fit, and changes in between-study heterogeneity (Supplementary Section 8).

## 3 Results

### 3.1 Study selection and characteristics

The searches of PubMed, Embase, Cochrane Library, and Web of Science identified 4,811 records. After 1,295 duplicates were removed, 3,516 records remained. Title and abstract screening used an active-learning-assisted strategy of “30% screening proportion +10% consecutive irrelevant records” in ASReview (Campos et al., 2024). A total of 188 potentially relevant reports proceeded to full-text assessment. Of these, 153 were excluded: 123 did not meet the inclusion criteria, 6 were abstracts or conference papers, 10 had ineligible designs, 2 had unobtainable full texts, and 12 had ineligible outcomes. The review included 35 randomized controlled trials. Figure 1 presents the screening process, and Table 1 and Supplementary Table 3 summarize study characteristics.

**FIGURE 1:**
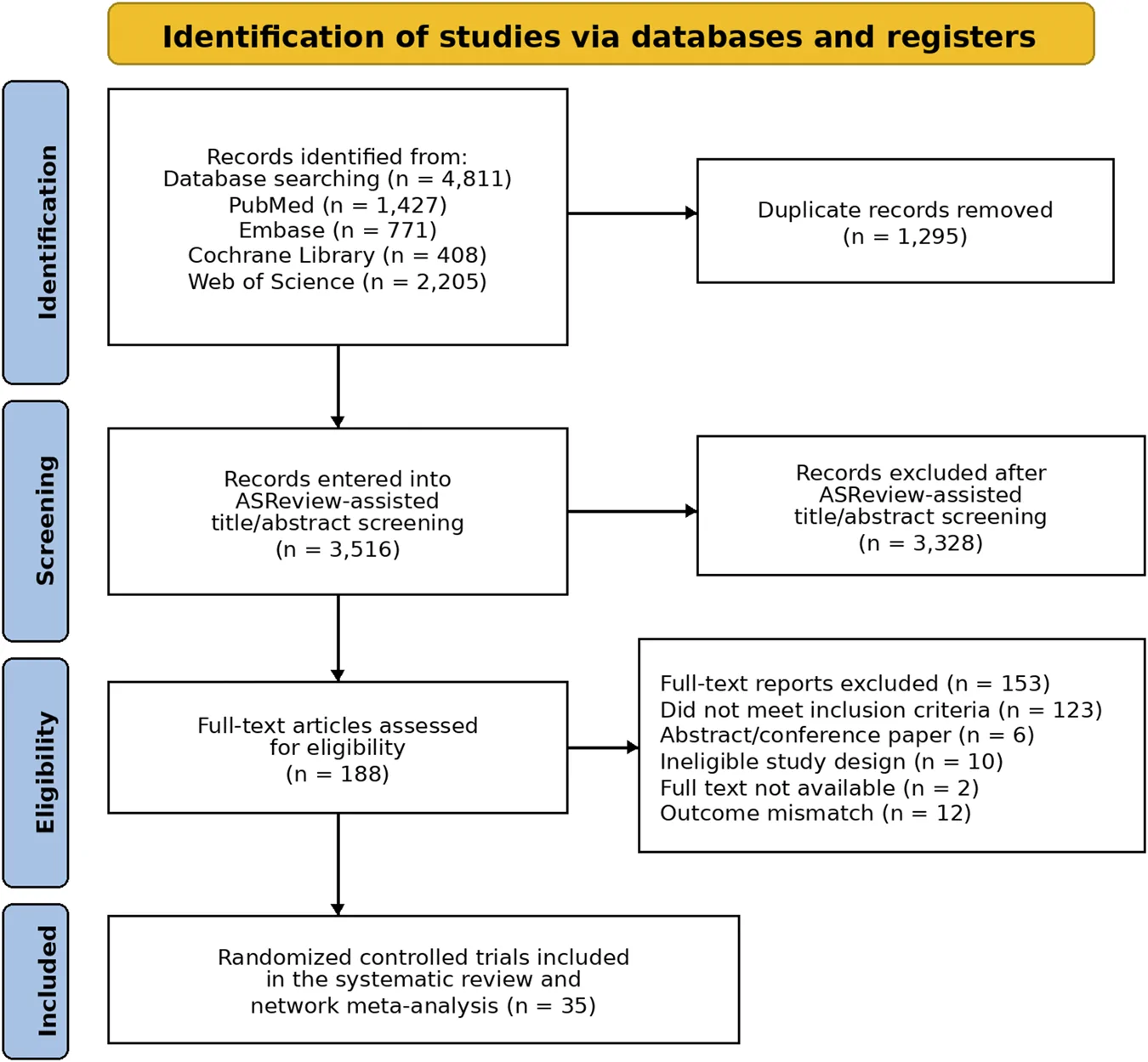
Flow diagram of literature search and study selection.

**TABLE 1:** Characteristics of the included studies.

Study	Sample size	Age, years	Population/sex	Intervention category and comparison	Study design	Outcomes and time points
(Antonialli et al., 2014)	10 vs. 10	24.10 ± 1.52	healthy untrained men	PHO vs. CON/PBO	RCT	CK, DOMS; 24 h, 48 h
(Boobphachart et al., 2017)	17 vs. 17 vs. 17	41.7 ± 8.6	healthy untrained women	KT; STR vs. CON/PBO	RCT	CK; 24 h, 48 h
(Cheng et al., 2022)	13 vs. 12	18.5 ± 2.9 vs. 19.3 ± 4.2	male field athletes	VIB vs. CON/PBO	RCT	CK, DOMS, IL-6; 24 h, 48 h
(Cheng et al., 2025)	11 vs. 11	20.3 ± 1.5 vs. 20.0 ± 1.2	field athletes (13 women, 9 men)	VIB vs. CON/PBO	RCT	CK, DOMS, IL-6; 24 h, 48 h
(De Marchi et al., 2017)	8 vs. 8 vs. 8	25.30 ± 3.32	healthy physically active men	CRY; PHO vs. CON/PBO	RCT	CK; 24 h, 48 h
(Duffield et al., 2010)	11	20.9 ± 2.7	trained team-sport athletes (sex NR)	COMP vs. CON/PBO	Crossover RCT	CK; 24 h
(Fonseca et al., 2016)	8	24.0 ± 3.6	healthy male athletes	CWI vs. CON/PBO	Crossover RCT	CK; 24 h, 48 h
(Guilhem et al., 2013)	12 vs. 12	23.9 ± 1.4 vs. 25.2 ± 1.1	healthy men	CRY vs. CON/PBO	RCT	CK, DOMS, IL-6; 24 h, 48 h
(Kim et al., 2017)	8 vs. 8	23.13 ± 3.76 vs. 24.25 ± 1.28	healthy male university students	COMP vs. CON/PBO	RCT	CK, DOMS; 24 h, 48 h
(Machado et al., 2020a)	10 vs. 10	26.83 ± 6.02	healthy men	PHO vs. CON/PBO	RCT	CK, DOMS; 24 h, 48 h
(Oakley et al., 2013)	20 vs. 10	24.0 ± 4.0	healthy trained and untrained adults (16 women, 17 men enrolled)	CRY vs. CON/PBO	RCT	CK; 24 h, 48 h
(Pruscino et al., 2013)	8	21.9 ± 2.3	highly trained male field hockey players	COMP vs. CON/PBO	Crossover RCT	CK, IL-6; 24 h, 48 h
(Qu et al., 2020)	12	21.00 ± 0.95	male middle- and long-distance runners	CWI; CWT; WBC vs. CON/PBO	Crossover RCT	CK, DOMS; 24 h, 48 h
(Eston and Peters, 1999)	8 vs. 7	22.0 ± 2.0	female students	CRY vs. CON/PBO	RCT	CK; 24 h, 48 h
(Torres et al., 2013)	14 vs. 14	21.4 ± 1.9	healthy untrained men	STR vs. CON/PBO	RCT	CK, DOMS; 24 h, 48 h
(Wei et al., 2025)	5 vs. 5 vs. 5 vs. 5 vs. 5 vs. 5	21.1 ± 1.4	healthy male university students	CWI; ES; STR; VIB; MAS vs. CON/PBO	RCT	CK, IL-6; 24 h, 48 h
(De Paiva et al., 2016)	10 vs. 10	24.98 ± 5.90	healthy untrained men	PHO; CRY vs. CON/PBO	RCT	CK, DOMS; 24 h, 48 h
(Hazar et al., 2019)	27 vs. 27	22.5 ± 2.0 vs. 23.0 ± 2.0	healthy nonathletic adults (28 women, 26 men)	KT vs. CON/PBO	RCT	CK, DOMS; 48 h
(Douris et al., 2006)	9 vs. 9	24.1 ± 3.0	healthy adults (sex NR)	PHO vs. CON/PBO	RCT	DOMS; 24 h, 48 h
(Fleckenstein et al., 2016)	12 vs. 12	23.6 ± 2.8	healthy adults (14 women, 10 men)	AC vs. CON/PBO	RCT	DOMS; 24 h, 48 h
(Gatterer et al., 2015)	7	25.6 ± 2.2	sport students (3 women, 4 men)	ES vs. CON/PBO	Crossover RCT	DOMS; 24 h, 48 h
(Itoh et al., 2008)	7 vs. 10	NR	healthy student volunteers (13 women, 17 men enrolled)	AC vs. CON/PBO	RCT	DOMS; 24 h, 48 h
(Kirmizigil et al., 2020)	22	21.36 ± 1.68	healthy male amateur athletes	KT vs. CON/PBO	Crossover RCT	DOMS; 24 h, 48 h
(Lee et al., 2015)	18 vs. 19	23.5 ± 1.2 vs. 22.5 ± 1.4	healthy men	KT vs. CON/PBO	RCT	DOMS; 24 h, 48 h
(Mcloughlin et al., 2004)	14	23.9 ± 4.6	healthy men	ES vs. CON/PBO	Crossover RCT	DOMS; 24 h, 48 h
(Cerqueira et al., 2015)	7 vs. 6	22 ± 2.65 vs. 20 ± 2.45	healthy men	COMP vs. CON/PBO	RCT	DOMS; 24 h, 48 h
(Szymura et al., 2016)	10	21.2 ± 1.33	healthy men	KT vs. CON/PBO	Crossover RCT	DOMS; 24 h, 48 h
(Xue et al., 2023)	8 vs. 8 vs. 8	19.75 ± 1.59	healthy male college students without regular exercise training	KT; COMP vs. CON/PBO	RCT	DOMS, IL-6; 24 h, 48 h
(Krueger et al., 2019)	11	25.9 ± 2.1	healthy male athletes	WBC vs. CON/PBO	Crossover RCT	IL-6; 24 h
(Kay et al., 2018)	13 vs. 13	27.8 ± 8	recreationally active adults (10 women, 16 men)	STR vs. CON/PBO	RCT	CK; 24 h
(Aver et al., 2016)	7 vs. 7	18.81 ± 0.80	high-level male soccer athletes	LLLT vs. CON/PBO	RCT	CK, DOMS, IL-6; 24 h, 48 h
(Xia et al., 2022)	8 vs. 8	24.12 ± 1.03	healthy male college students	LIFU vs. CON/PBO	RCT	CK, DOMS; 24 h, 48 h
(Sudhakar et al., 2023)	16 vs. 16	20.4 ± 2.1 vs. 21.1 ± 2.8	male novice athletes	SW vs. CON/PBO	RCT	CK; 24 h, 48 h
(Pinto et al., 2022)	12	27 ± 5.54	male amateur CrossFit® athletes	PHO vs. CON/PBO	Crossover RCT	CK, IL-6; 24 h, 48 h
(Baroni et al., 2010)	18 vs. 18	25.35 ± 3.41 vs. 24.28 ± 5.48	healthy physically active male students	LLLT vs. CON/PBO	RCT	CK, DOMS; 24 h, 48 h

AC, acupuncture; CK, creatine kinase; COMP, compression therapy; CON/PBO, combined control/placebo condition; CWI, cold-water immersion; CWT, contrast-water therapy; CRY, local cryotherapy; DOMS, delayed-onset muscle soreness; ES, electrical stimulation/electrotherapy; IL-6, interleukin-6; KT, kinesiology taping; LIFU, low-intensity focused ultrasound; LLLT, laser-only low-level laser therapy; MAS, massage; NR, not reported; PHO, LED-containing photobiomodulation regimens; RCT, randomized controlled trial; STR, stretching; SW, shock wave therapy; VIB, vibration therapy; WBC, whole-body cryotherapy.

### 3.2 Risk-of-bias assessment

Risk-of-bias assessment results are presented in Supplementary Tables 4, 5. Among the 25 parallel-group randomized controlled trials, one was judged to be at low risk, 12 were judged to raise some concerns, and 12 were judged to be at high risk. Among the 10 randomized crossover trials, four were judged to raise some concerns and six were judged to be at high risk; no crossover trial was judged to be at overall low risk.

At the domain level, outcome measurement was the most frequent source of high risk among parallel-group trials (12/25). This judgment largely reflected absent or unclear blinding of participants or outcome assessors for subjective soreness ratings. Missing outcome data conferred high risk in 2/25 trials. Selective reporting raised concerns in most parallel-group trials because protocols or prespecified analysis plans were unavailable. Among 10 randomized crossover trials, 5 had high risk for outcome measurement. One additional trial had high risk because period and carryover effects were inadequately addressed. These limitations are particularly relevant to DOMS because knowledge of the assigned intervention may influence self-reported soreness. Risk-of-bias judgments therefore informed the certainty assessment and the cautious interpretation of effect estimates and rankings (Supplementary Tables 4, 5).

For randomized crossover trials, some studies did not adequately describe randomization of intervention sequence, the washout period, or the methods used to control for period effects or carryover effects, further increasing the overall risk of bias.

### 3.3 Network structure

Separate networks were constructed for DOMS, CK, and IL-6 at 24 and 48 h after exercise. Most networks were connected mainly through the CON/PBO node, with relatively few direct comparisons among active recovery interventions. The DOMS and CK networks were relatively well connected, whereas the IL-6 networks included fewer studies and were considerably sparser. Accordingly, the IL-6 results were interpreted primarily as exploratory evidence regarding inflammatory responses, and the IL-6 networks were not included in the main sensitivity analyses.

Potential effect modifiers were not distributed uniformly across direct treatment comparisons (Supplementary Table 36). Studies of CWI and VIB more often used systemic or whole-body exercise models and included trained participants. In contrast, studies of PHO and CRY more often used localized exercise-damage models and untrained or recreational participants. Although better-supported comparisons showed some overlap, important imbalances remained for several treatment contrasts. Exploratory network meta-regression did not identify consistent treatment–covariate interactions in the DOMS or CK networks. The transitivity assumption was therefore considered plausible but not definitively established. Indirect comparisons involving sparse or single-study nodes were interpreted cautiously.

For the light-based treatment nodes, (Baroni et al., 2010; Aver et al., 2016; Antonialli et al., 2014) and (De Paiva et al., 2016) used knee-extensor eccentric exercise protocols comprising 75 contractions and treated the quadriceps region (Supplementary Table 3A). These shared features support the clinical rationale for comparing the represented configurations and were considered alongside participant characteristics and treatment timing. The scope and precision of the available evidence were taken into account when interpreting comparisons across the two nodes.

### 3.4 Network meta-analysis results

#### 3.4.1 DOMS

The DOMS results showed time-point-specific differences in the relative effects of the interventions. The network plots and SUCRA-based cumulative ranking results for DOMS at 24 and 48 h are presented in Figures 2, 3, respectively. At 24 h, WBC (MD = −38.92, 95% CrI: −69.08 to −9.15), LIFU (MD = −34.54, 95% CrI: −62.45 to −6.61), and PHO (MD = −18.20, 95% CrI: −32.91 to −3.72) were associated with lower soreness than CON/PBO. At 48 h, LIFU (MD = −65.81, 95% CrI: −92.30 to −39.66) and WBC (MD = −40.03, 95% CrI: −67.74 to −12.44) were also associated with lower soreness than CON/PBO. The corresponding point estimates were also below zero in forest plots referenced against COMP (Figure 4). These findings suggest that the relative effects of some interventions may vary across the early recovery period. However, most active interventions were connected through CON/PBO, and direct comparisons between active interventions were limited. SUCRA rankings were therefore treated as supplementary rather than as stand-alone evidence of comparative benefit. Although LIFU ranked highest and had the largest estimated effect at 48 h, this result depended primarily on one small study. The WBC estimates likewise came from a single small crossover trial. Both findings were supported by very-low-certainty evidence and should not be interpreted as establishing comparative superiority.

**FIGURE 2:**
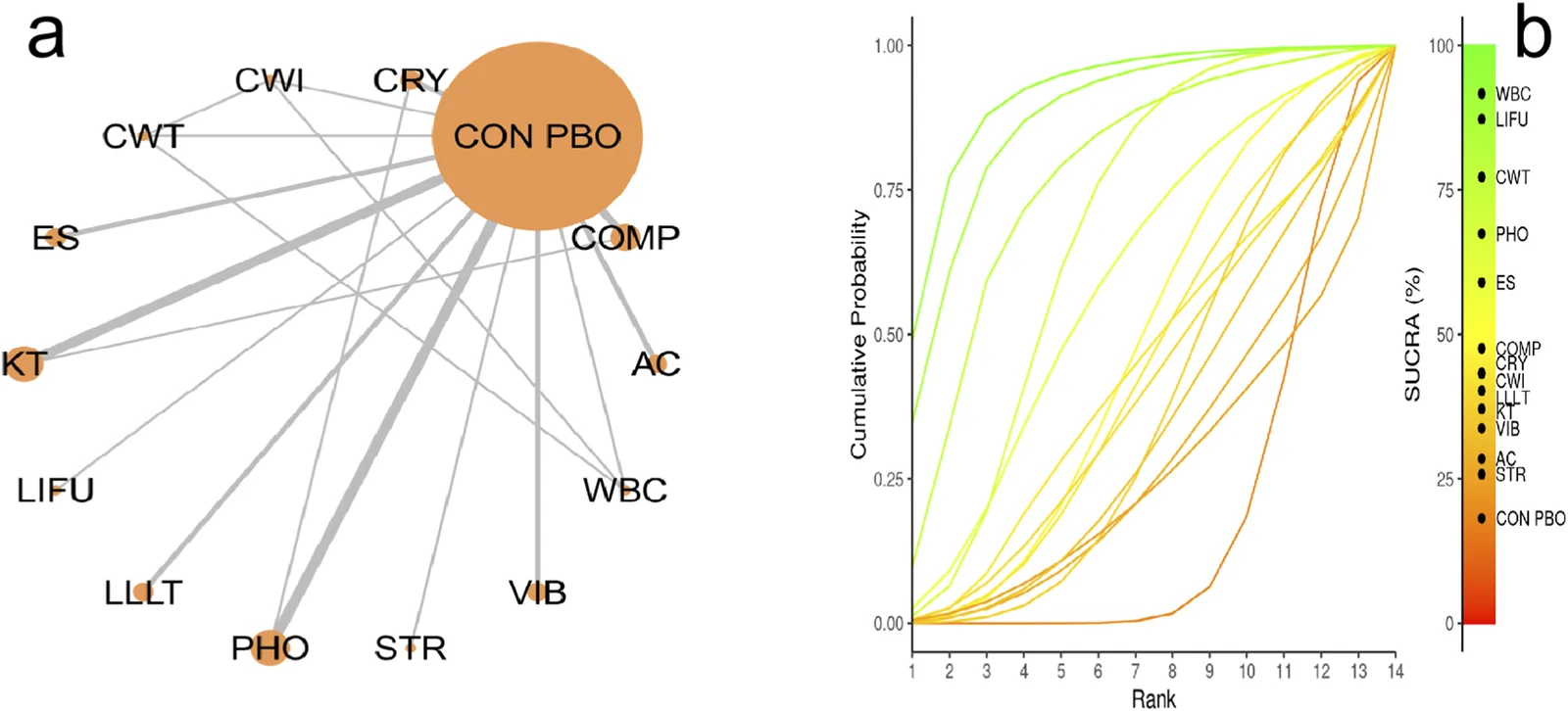
Effects of different recovery and regeneration modalities on DOMS at 24 h. **(a)** Network plot of the direct comparisons contributing to the network meta-analysis. Node size represents the number of participants assigned to each intervention. Edges represent direct comparisons, and edge thickness represents the amount of direct evidence. **(b)** Bayesian ranking panel. The cumulative probability represents the probability of a given intervention achieving a particular rank. Surface under the cumulative ranking curve (SUCRA) values summarize the relative rankings of the interventions; higher values indicate a higher relative rank but do not, by themselves, establish comparative superiority.

**FIGURE 3:**
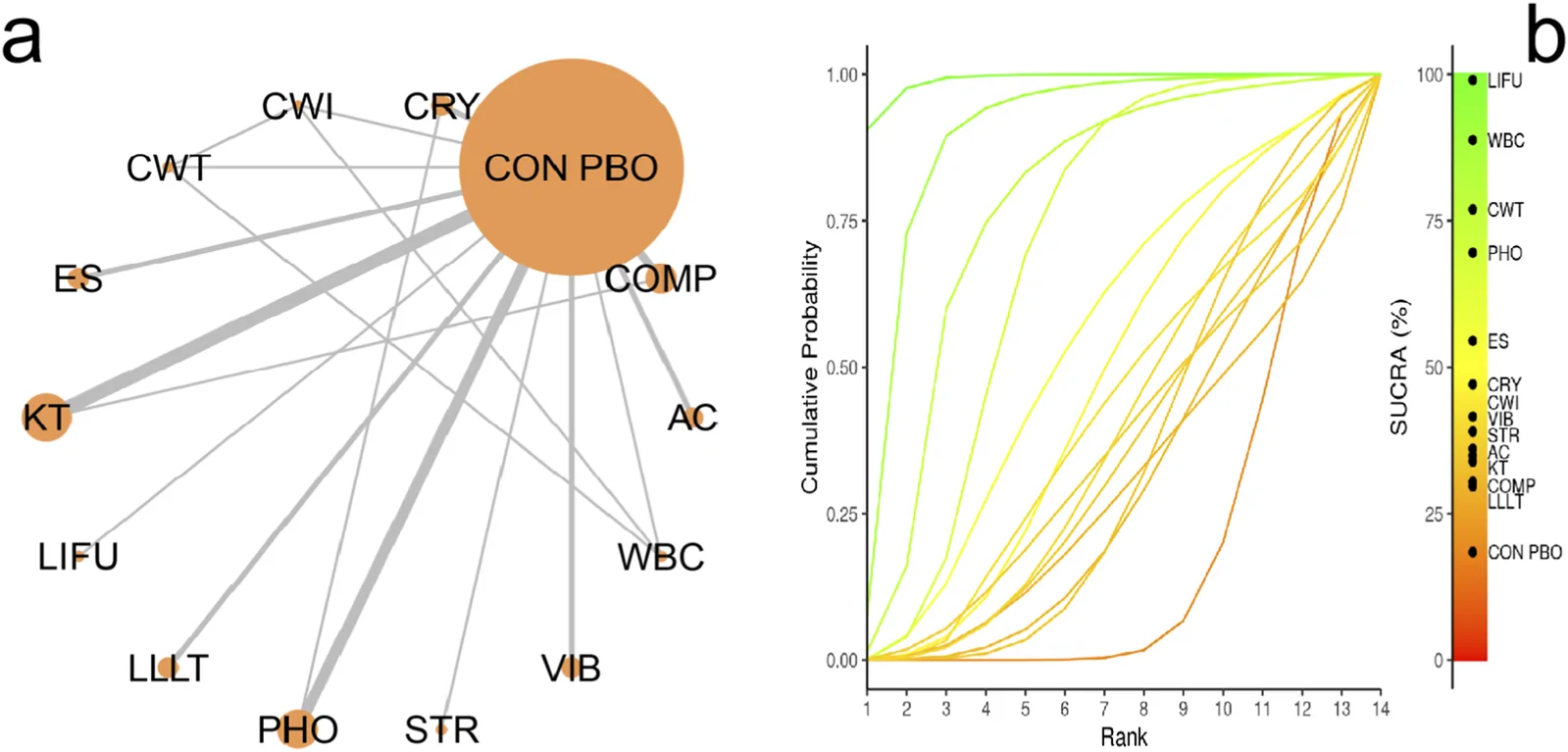
Effects of different recovery and regeneration modalities on DOMS at 48 h. **(a)** Network plot. **(b)** Bayesian cumulative ranking probability plot.

**FIGURE 4:**
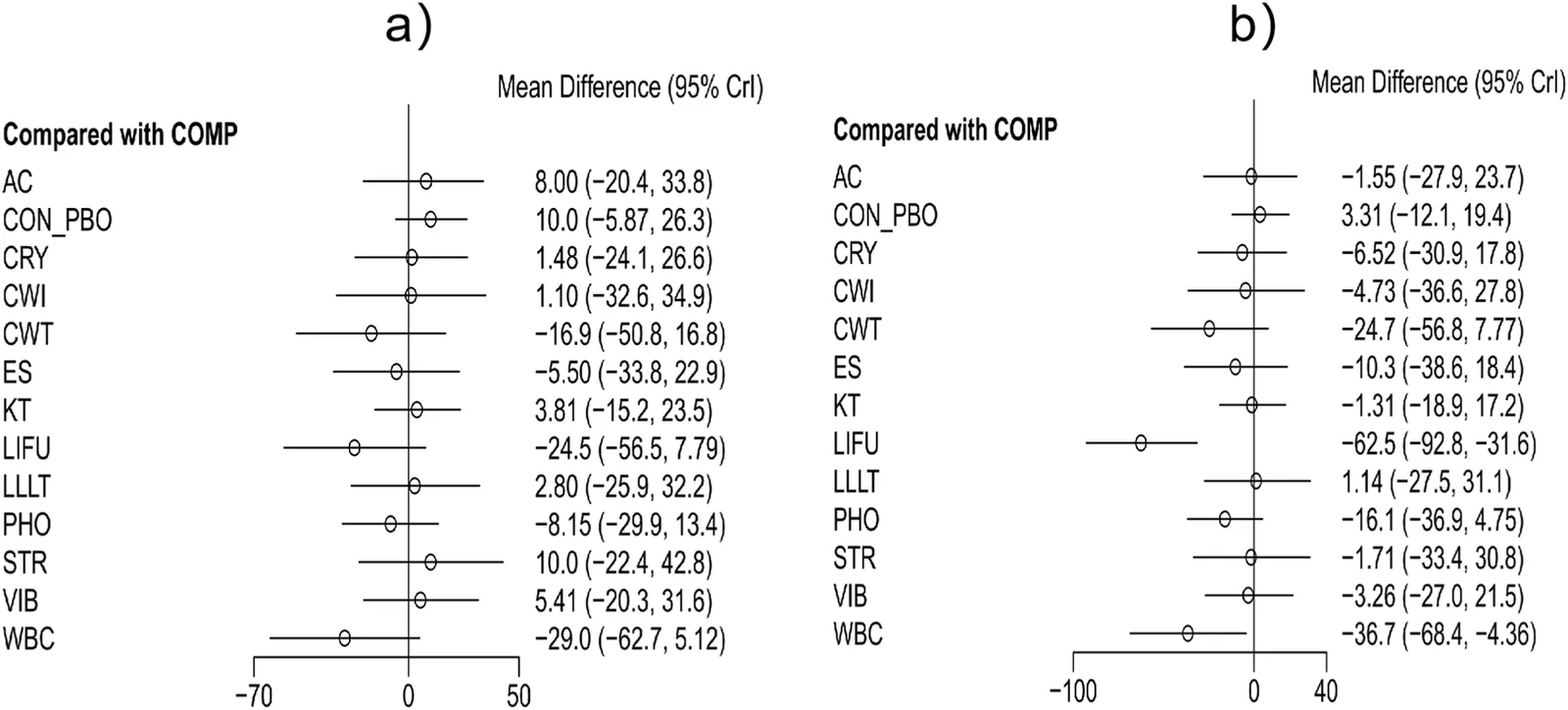
Forest plot for DOMS at 24 h and 48 h. **(a)** 24h DOMS, **(b)** 48h DOMS.

#### 3.4.2 CK

At 24 h, LLLT (MD = −249.70, 95% CrI: −502.23 to −1.08) and PHO (MD = −199.48, 95% CrI: −329.27 to −80.63) were associated with lower CK levels, with 95% CrIs excluding 0. At 48 h, LLLT (MD = −499.96, 95% CrI: −831.59 to −203.59) and PHO (MD = −252.50, 95% CrI: −420.45 to −87.47) were also associated with lower CK levels than CON/PBO. Estimates for most other interventions were imprecise and included the null. Estimated CK reductions were observed for LLLT and PHO at both time points. However, CK is an indirect biochemical marker and should not be interpreted alone as evidence of structural or functional recovery. The network plot and SUCRA-based cumulative ranking results for CK at 24 h are presented in Figure 5. The corresponding forest plots for CK at 24 and 48 h are shown in Figure 6, while the network plot and SUCRA-based cumulative ranking results at 48 h are presented in Figure 7.

**FIGURE 5:**
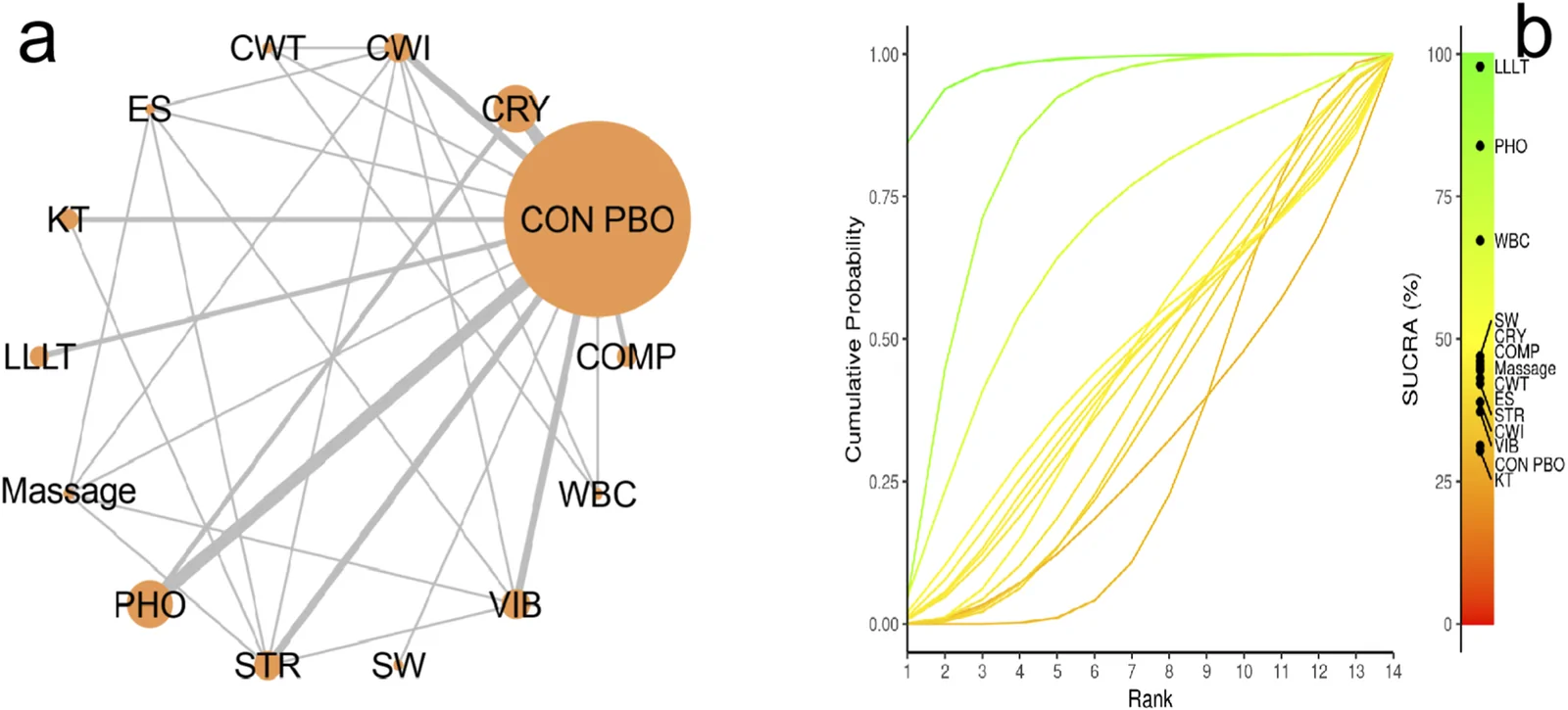
Effects of different recovery and regeneration modalities on CK at 24 h. **(a)** Network plot. **(b)** Bayesian cumulative ranking probability plot.

**FIGURE 6:**
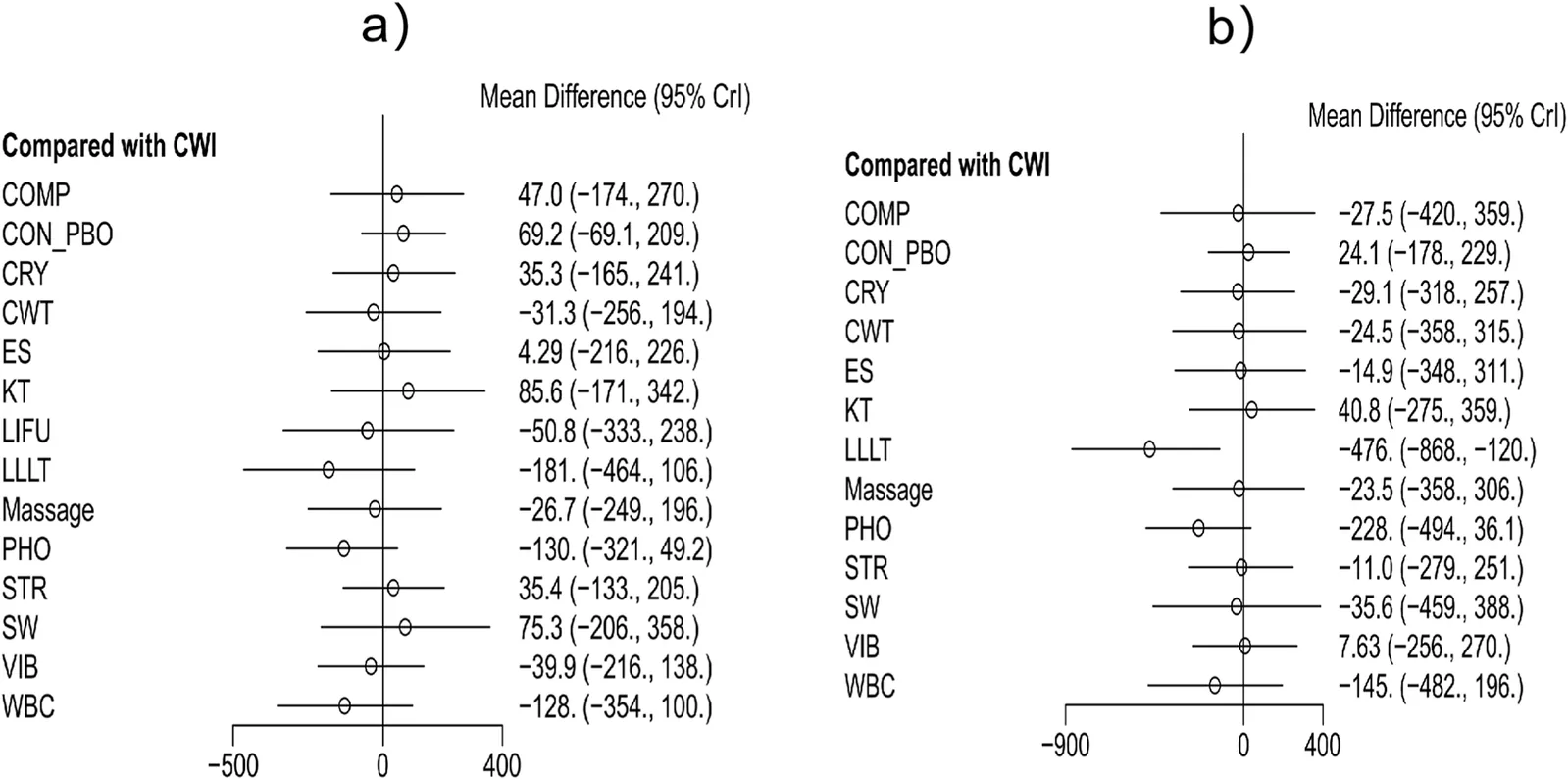
Forest plot for CK at 24 h and 48 h. **(a)** 24h CK **(b)** 48h CK.

**FIGURE 7:**
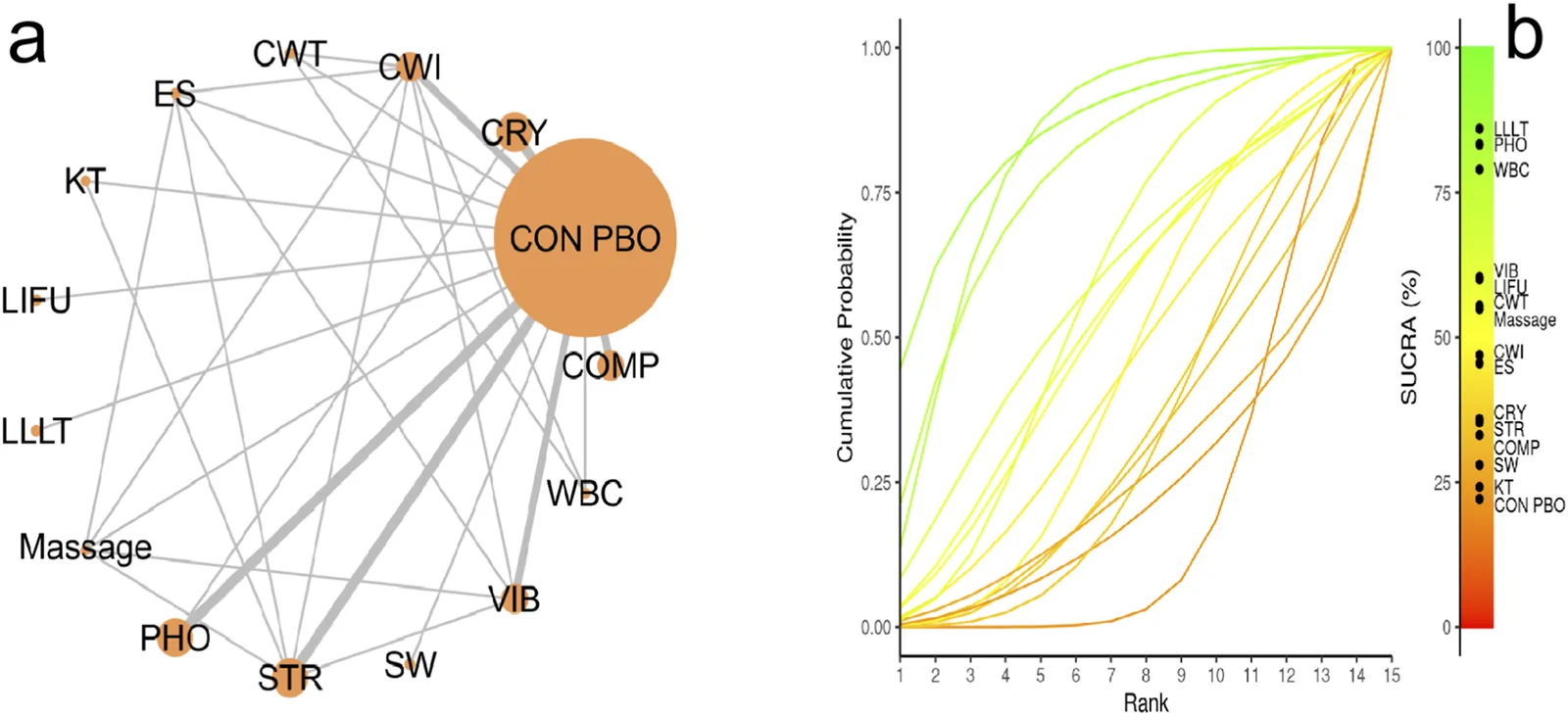
Effects of different recovery and regeneration modalities on CK at 48 h. **(a)** Network plot. **(b)** Bayesian cumulative ranking probability plot.

Comparisons between active treatments were less precise. For example, the 95% CrI for PHO versus CWI included 0, and the estimate for WBC versus CWI was also imprecise. Comparative superiority among active interventions could therefore not be established. Previous evidence similarly indicates that CK responses vary with exercise model, intervention parameters, and measurement timing (Machado AF. et al., 2020; Li et al., 2024; Moore et al., 2022).

The divergence between the DOMS and CK rankings is therefore not a contradiction but instead suggests that pain relief and muscle-damage-related biochemical responses are not necessarily synchronous. (Nosaka et al., 2002) noted that DOMS does not reliably reflect the degree of muscle damage from eccentric exercise, and (Baird et al., 2012) likewise emphasized that CK is strongly influenced by individual variability and sampling time. Given that the network was connected mainly through CON/PBO, with limited direct comparisons among active interventions, SUCRA should be regarded only as a supplementary indicator and should not be translated directly into an intervention recommendation.

#### 3.4.3 IL-6

The IL-6 network results indicated considerably greater uncertainty regarding post-exercise inflammatory responses to different recovery interventions. Results are shown in Supplementary Figures 1-3. At 24 h, CWI (MD = −97.26, 95% CrI: −186.18 to −15.25) and electrical stimulation (ES; MD = −91.35, 95% CrI: −177.45 to −11.83) were associated with lower IL-6 levels relative to CON/PBO. At 48 h, LLLT (MD = −79.34, 95% CrI: −153.29 to −5.12) and CWI (MD = −66.19, 95% CrI: −130.87 to −17.27) were associated with lower IL-6 levels than CON/PBO. Given the small number of studies, sparse network structure, limited direct comparisons among active interventions, and generally wide intervals, these findings should be regarded as hypothesis-generating evidence relevant to potential inflammatory mechanisms rather than as an independent basis for recommending an intervention. In particular, although WBC was associated with lower DOMS at both time points, its IL-6 results did not show the same pattern, further indicating that pain relief, muscle-damage-related biochemical responses, and inflammatory changes are not necessarily synchronous.

#### 3.4.4 Bayesian model diagnostics and inconsistency assessment

All six outcome and time-point networks showed satisfactory Bayesian model convergence. Brooks–Gelman–Rubin shrink factors approached 1, trace plots showed satisfactory chain mixing without persistent separation or trends, and posterior density plots were stable (Supplementary Table 33). UME inconsistency models did not materially improve model fit over the corresponding consistency models. DIC differences were small across the six networks (ΔDIC: −0.148 to 0.339; Supplementary Table 34; Supplementary Figure 4). Node-splitting was estimable for 13 treatment contrasts in the DOMS and CK networks. No statistically detectable disagreement between direct and indirect evidence was identified (all P > 0.05). Node-splitting was not estimable for IL-6 at either 24 h or 48 h because no contrast had sufficient independent direct and indirect evidence (Supplementary Table 35).

GRADE assessment indicated low-to-moderate certainty for direct evidence and generally low certainty for indirect evidence across the 13 evaluable DOMS and CK comparisons. All 13 corresponding network estimates were rated as very low certainty. Source-specific assessment was not feasible for IL-6 because independent direct and indirect evidence was unavailable (Supplementary Table 17A).

### 3.5 Sensitivity analysis

Sensitivity analyses examined the influence of individual studies, small samples, crossover designs, and comparator classification on effect estimates, rankings, and network structure (Supplementary Section 6,11; Supplementary Figures 6-9; Supplementary Tables 18,19B,37-39).

Leave-one-out analyses indicated that the robustness of several estimates varied. The LIFU node disappeared after exclusion of Xia 2022, confirming that this estimate was based on a single study. The PHO estimate for DOMS at 24 h became less precise after exclusion of Antonialli 2014, whereas PHO for CK at 24 h and LLLT for CK at 48 h had 95% CrIs below zero after removal of influential studies. Exclusion of Qu 2020 removed several cold-therapy nodes from the CK 48-h network, indicating dependence on limited evidence and network connectivity.

Excluding randomized crossover trials generally preserved the principal effect directions, with increased uncertainty for some comparisons. Separating CON from PBO_SHAM also generally retained the direction of the principal DOMS and CK point estimates, although some 95% CrIs widened to include zero (Supplementary Table 39). Control-split analyses were not estimable for IL-6 because the networks became disconnected. Overall, robustness was assessed jointly from sensitivity results, precision, and the evidence supporting each node.

### 3.6 Network meta-regression analysis

We constructed 6 unadjusted random-effects models and 33 univariable shared-β network meta-regression models to examine whether study-level characteristics influenced effect estimates. The corresponding networks included 8 to 23 studies. Supplementary Section 8 and Supplementary Table 29 provide the network-specific study counts.

Covariates included in the meta-regression were the exercise-induced damage model, intervention site, training status, study design, overall risk of bias, and small-study status. Model interpretation was based mainly on the regression coefficient (β) and its 95% CrI, the DIC, residual deviance, and heterogeneity standard deviation (τ). Given that some networks were sparse, meta-regression was used to explore potential effect modifiers.

Most β estimates in the DOMS and CK networks had 95% CrIs that included the null. Covariate adjustment did not consistently improve DIC or reduce between-study heterogeneity. We therefore considered CrI width, model fit, and changes in τ together when evaluating the precision and stability of potential effect-modification patterns.

At 48 h, several IL-6 covariates produced non-null β estimates, but each model included only eight studies and did not reduce heterogeneity. Because 33 exploratory models were examined without multiplicity adjustment, isolated signals may be false-positive or unstable. These coefficients were therefore not considered sufficient on their own to establish effect modification.

## 4 Discussion

### 4.1 Main findings

The relative effects of recovery and regeneration interventions differed by outcome and recovery time point. WBC and PHO were associated with lower DOMS, and LLLT and PHO with lower CK levels, at both 24 and 48 h; IL-6 findings varied across interventions and time points. Thus, the relative performance of each intervention depended on the outcome and assessment time point. Lower DOMS did not imply parallel improvement in muscle-damage-related biochemical or cytokine responses. The practical relevance of these findings was also outcome-specific. DOMS was standardized to a 0–100 mm visual analog scale; however, this review did not prespecify a clinically important difference, and no universal threshold was applicable across the included populations and exercise protocols. Therefore, a 95% CrI excluding 0 should not, by itself, be interpreted as establishing clinical superiority. Reductions in DOMS may inform symptom management, whereas changes in CK and IL-6 do not directly indicate functional recovery. Selection of recovery strategies may therefore consider the target outcome, recovery time window, evidence certainty, and network structure.

Exploratory network meta-regression did not identify any single study-level characteristic that consistently explained the main effect differences for DOMS or CK. These differences may instead reflect the combined influence of multiple clinical and methodological factors.

### 4.2 Interpretation of differences among recovery intervention outcomes

The DOMS results suggest that the effects of different recovery interventions on pain relief may not be explained entirely by muscle damage itself and may instead be related partly to nociceptive modulation, local tissue status, and neural conduction. Estimated reductions in DOMS with cold-related interventions may partly reflect the effects of low-temperature stimulation, which can lower local tissue temperature, slow sensory nerve conduction velocity, attenuate nociceptive input, and reduce local swelling and tenderness through vasoconstriction and fluid redistribution; contrast-water therapy may further influence local circulation through repeated vasomotor cycling (White and Wells, 2013; Wilcock et al., 2006; Hohenauer et al., 2015). These mechanisms may contribute to changes in pain and subjective recovery (Nosaka et al., 2002; Wilke and Behringer, 2021). The estimated DOMS reductions with LIFU and PHO suggest that, in addition to cold stimulation, mechanical stimulation or photobiomodulation may influence the local tissue microenvironment and sensory afferent input. However, because DOMS is also influenced by psychological expectations, pain sensitivity, and measurement method, relief of soreness should not be interpreted as evidence that structural muscle recovery has occurred in parallel.

The CK results require a different interpretation from those for DOMS. Phototherapy-related interventions were associated with lower CK levels at both time points, suggesting that their effects may be more closely related to cellular metabolic and biochemical recovery processes following muscle damage rather than solely to changes in pain perception. Both laser-only and LED-containing photobiomodulation regimens may attenuate exercise-induced changes in muscle cell membrane permeability and related enzymatic responses by influencing the mitochondrial respiratory chain, ATP generation, redox balance, and inflammatory signaling (Chen et al., 2025). However, because CK levels are readily influenced by individual physiological variability, the amount of muscle mass affected, and sampling time, a reduction in CK is more appropriately regarded as an indirect indicator of attenuated muscle-damage-related biochemical responses rather than evidence that structural damage has already been repaired (Baird et al., 2012). Recent systematic-review evidence also suggests that CK and other muscle-damage markers may show differing response patterns, supporting the use of complementary biochemical measures to characterize recovery (Jozi et al., 2026).

LIFU had a large estimated reduction in soreness at both DOMS time points, which may relate to modulation of the local tissue microenvironment and sensory afferent input, but the available evidence is derived mainly from a single small study (Xia et al., 2022). Kinesiology taping (KT), by contrast, did not show a consistent relative advantage within this network. This does not indicate that KT is ineffective; rather, its effects may be outcome-specific and inconsistent across studies. (Hazar et al., 2019) found that KT reduced muscle soreness but did not improve isometric strength, vertical jump, lactate dehydrogenase (LDH), myoglobin, or C-reactive protein (CRP), whereas CK increased. Consequently, when multiple recovery methods are compared simultaneously, an effect pattern as limited and inconsistent as that observed for KT is less likely to yield a consistent relative advantage (Hazar et al., 2019; Kirmizigil et al., 2020; Lee et al., 2015).

The IL-6 results should be regarded as exploratory evidence regarding inflammatory modulation rather than as an independent basis for recommendation. IL-6 is not simply a pro-inflammatory marker; it is also an important myokine released by skeletal muscle during exercise, involved in energy metabolism, immune regulation, and tissue repair. Pedersen and Febbraio (Pedersen and Febbraio, 2008) noted that exercise-induced IL-6 has complex endocrine and immunoregulatory roles, and (Peake et al., 2017) likewise emphasized that the post-exercise inflammatory response is itself part of the muscle repair process. Recent clinical evidence likewise shows that reductions in CK and some inflammatory markers may occur without a clear change in IL-6, highlighting the complementary information provided by these biomarkers during post-exercise recovery (Bideshki et al., 2026). A decrease in IL-6 should therefore not be interpreted simplistically as indicating “better recovery.”

### 4.3 Exploratory interpretation of sources of heterogeneity

The heterogeneity observed in this study may reflect clinical and methodological differences across studies rather than statistical variation alone (Higgins et al., 2003). Exercise-induced damage may arise from eccentric loading of a single local muscle group or from running, jumping, sport-specific activity, or high-intensity combined training; interventions may likewise act on a local muscle group, the lower limbs, or the whole body (Kanda et al., 2013; Chalchat et al., 2022). These differences may alter the time course of pain perception, biochemical marker release, and inflammatory responses, thereby influencing estimates for the same intervention across different networks (Baird et al., 2012).

Exploratory network meta-regression did not identify a reproducible single-covariate explanation for heterogeneity in the DOMS or CK networks. The amount of information and precision varied across networks, and multiple models were evaluated. Individual β estimates were therefore interpreted alongside their 95% CrIs, DIC, residual deviance, and changes in τ rather than as stand-alone evidence of effect modification (Dias et al., 2013).

### 4.4 Comparison with previous studies

The findings of this study are broadly consistent with previous evidence. Previous meta-analyses have shown that water immersion, contrast-water therapy, and cryotherapy may reduce DOMS, although biochemical markers such as CK and IL-6 do not necessarily improve synchronously (Dupuy et al., 2018; Hohenauer et al., 2015). The estimated DOMS reductions with cold-related interventions were in line with this evidence, while the phototherapy findings at 24 and 48 h were broadly consistent with those of a previous network meta-analysis of PBMT (Chen et al., 2025).

Some findings differed from those of previous studies. Dupuy et al. reported favorable effects of massage on DOMS and some biochemical markers, whereas the present network did not show a consistent relative advantage for massage. CWI was also prominent in previous research, whereas estimated DOMS reductions were observed for WBC and CWT in the present analysis (Dupuy et al., 2018; Moore et al., 2022). These differences may relate to analytical methods, intervention classification, and evaluation time windows. Traditional meta-analyses mainly perform pairwise comparisons between an intervention and a comparator, whereas network meta-analysis emphasizes relative effects across multiple interventions. In addition, this study differentiated among modes of cryotherapy and phototherapy and focused on the 24–48 h recovery period. The observed differences are therefore more appropriately interpreted as reflecting differences in study design and evidence structure rather than direct contradictions in conclusions.

### 4.5 Strengths and limitations

This study used Bayesian NMA to compare multiple recovery and regeneration interventions simultaneously. DOMS, CK, and IL-6 were evaluated separately at 24 h and 48 h. This approach characterized early recovery across subjective symptoms, muscle-damage-related biochemical responses, and inflammatory responses. CINeMA, sensitivity analyses, and exploratory network meta-regression assessed confidence, robustness, and potential sources of heterogeneity. Nevertheless, the study has several limitations. First, most networks were connected mainly through CON/PBO, and direct comparisons between active interventions were limited. Some relative effects therefore depended heavily on indirect evidence. Potential effect modifiers were not distributed uniformly across comparisons. Although exploratory meta-regression found no consistent effect-modification pattern, residual intransitivity cannot be excluded, particularly for sparse and single-study comparisons. For light-based interventions, PHO represents a broader category of LED-containing regimens, including some devices with a static magnetic field. The estimates therefore describe the treatment configurations represented in each node and do not isolate the contributions of individual light sources or magnetic components. Second, the combined CON/PBO node provided a common reference across recovery interventions, consistent with previous NMAs (Gianola et al., 2022; Recchia et al., 2022). Sensitivity analyses separating CON and PBO_SHAM broadly supported the direction of the principal estimates, with reduced precision for some comparisons. Potential differences in expectancy remain relevant to subjective DOMS; the separate-control analyses provide complementary information for interpreting these estimates (Supplementary Table 39). Third, several emerging interventions and the IL-6 networks were supported by few, generally small studies. Some estimates were therefore sensitive to individual datasets. Non-null estimates from sparse or single-study nodes and high SUCRA rankings should not be interpreted as robust evidence of clinical superiority. Control-split sensitivity analysis was not estimable for IL-6 because separating CON and PBO_SHAM disconnected the networks. Local inconsistency was also not estimable because no contrast had sufficient independent direct and indirect evidence. Fourth, the primary arm-based NMA did not explicitly model within-participant correlations for the 10 randomized crossover trials because paired data were unavailable. Excluding these trials broadly preserved the main effect directions, although uncertainty increased. Finally, the study did not include muscle strength, jump performance, sprint performance, or other performance outcomes. Improvements in DOMS or biochemical markers therefore do not establish synchronous recovery of function or athletic performance.

### 4.6 Future research

Future research should include more direct comparisons between active recovery strategies, particularly WBC and PHO for DOMS and LLLT and PHO for CK, to clarify the estimated reductions observed for the corresponding outcomes at both time points. LIFU showed a large estimated reduction in DOMS, but this finding requires replication in independent studies with larger samples. Subsequent trials should also assess DOMS, muscle-damage and inflammatory markers, and muscle strength, jump performance, or sport-specific performance together to clarify whether symptoms, biochemical responses, and functional recovery change in parallel. In addition, exercise-induced damage models, training status, intervention dose, and site of action should be reported in sufficient detail to facilitate identification of potential effect modifiers.

## 5 Conclusion

The relative effects of recovery and regeneration interventions were outcome- and time-specific, and no single method showed a consistent advantage across DOMS, CK, and IL-6. Effect estimates suggested lower DOMS with WBC and PHO and lower CK levels with LLLT and PHO at 24 and 48 h. LIFU had a large estimated effect on DOMS, but this finding requires confirmation in additional independent studies. The IL-6 findings should be considered exploratory evidence of potential inflammatory modulation. Selection of post-exercise recovery strategies should therefore consider the target outcome, recovery time window, evidence certainty, and network structure. Improvements in subjective symptoms should not be interpreted as evidence of parallel recovery in muscle-damage-related biochemical responses or overall function.

## Data Availability

The data analyzed in this systematic review and network meta-analysis were extracted from previously published studies. Relevant results and supporting information are provided in the article and supplementary material. Additional study-level extracted data used in the analyses are available from the corresponding author, Ming Wei (weiming2019@cupes.edu.cn), upon reasonable request.
