# Supplementary material for: Effects of different recovery and regeneration modalities on delayed-onset muscle soreness and post-exercise muscle damage markers: a systematic review and network meta-analysis
Source: Front Bioeng Biotechnol. 2026 Oct 26;14:1956053. doi: 10.3389/fbioe.2026.1956053 (PMC13745678; doi:10.3389/fbioe.2026.1956053)
Supplement: Supplementary file 1 [file Table1.docx]

**Effects of Different Recovery and Regeneration Modalities on Delayed-Onset Muscle Soreness and Post-Exercise Muscle Damage Markers: A Systematic Review and Network Meta-Analysis: Supplementary Material**

# **Contents**

[1 Search strategy	1](#_Toc13933)

[2. Eligibility criteria adopted by the PICOS framework	4](#_Toc12272)

[3. Characteristics of included studies	5](#_Toc28656)

[4. Risk of bias	9](#_Toc11015)

[4.1 Risk of bias in randomized controlled trials	9](#_Toc18030)

[4.2 Risk of bias in randomized crossover trials	10](#_Toc5220)

[5. Network meta-analysis	11](#_Toc30573)

[5.1 League tables	11](#_Toc8005)

[5.2 Certainty of evidence: direct, indirect, and network estimates	17](#_Toc19926)

[6. Sensitivity analyses	24](#_Toc17665)

[6.1 Potential studies with extreme effect sizes	24](#_Toc14170)

[6.2 Leave-one-out sensitivity analyses excluding potential extreme-effect studies	24](#_Toc30583)

[6.3 Robustness assessment for sparse and single-study findings	26](#_Toc21909)

[6.3.1 Statistical significance versus robustness	26](#_Toc29701)

[6.3.2 Interpretation of SUCRA rankings	26](#_Toc29127)

[6.4 Sensitivity analyses excluding small-sample studies	26](#_Toc8956)

[6.4.1 Sensitivity analysis excluding small-sample studies for DOMS	26](#_Toc11997)

[6.4.2 Sensitivity analysis excluding small-sample studies for CK	28](#_Toc4078)

[6.5 Sensitivity analyses excluding randomized crossover trials	29](#_Toc29969)

[6.5.1 Sensitivity analysis excluding randomized crossover trials for DOMS	29](#_Toc11139)

[6.5.2 Sensitivity analysis excluding randomized crossover trials for CK	30](#_Toc10824)

[7. Heterogeneity and prediction intervals	31](#_Toc14178)

[8. Exploratory network meta-regression	33](#_Toc4774)

[8.1 Rationale and covariate coding	33](#_Toc7723)

[8.2 Meta-regression methods	34](#_Toc20511)

[8.3 Summary of meta-regression results	34](#_Toc14631)

[8.4 Outcome-specific interpretation	36](#_Toc31143)

[8.5 Limitations of meta-regression	36](#_Toc370)

[9. Bayesian model-fit, convergence, and inconsistency diagnostics	37](#_Toc3721)

[9.1 Bayesian analysis specification	37](#_Toc26656)

[9.2 Model fit and heterogeneity	37](#_Toc8273)

[9.3 Deviance report for all studies	38](#_Toc16269)

[9.3.1 Residual deviance from NMA model and UME inconsistency model for all studies	38](#_Toc1631)

[9.3.2 Per-arm residual deviance for all studies	38](#_Toc4234)

[9.4 Inconsistency assessment	39](#_Toc12695)

[10. Transitivity assessment	40](#_Toc15728)

[11. Control-condition classification and sensitivity analyses	42](#_Toc979)

# **1. Search strategy**

Supplementary Table 1. Search strategy for PubMed, Cochrane Library, Web of Science, and Embase, May 2026.

**Search Strategy (n=4811)**

**PubMed (Results: 1427)**

((((adult*[Title/Abstract]) OR ("healthy adult"[Title/Abstract]) OR (athlete*[Title/Abstract]) OR (sport*[Title/Abstract]) OR ("physically active"[Title/Abstract]) OR (trained[Title/Abstract]) OR (untrained[Title/Abstract]) OR ("recreationally active"[Title/Abstract]))AND(("acute exercise"[Title/Abstract]) OR ("post-exercise"[Title/Abstract]) OR (postexercise[Title/Abstract]) OR ("after exercise"[Title/Abstract]) OR ("exercise recovery"[Title/Abstract]) OR ("recovery phase"[Title/Abstract]) OR ("exercise-induced muscle damage"[Title/Abstract]) OR (EIMD[Title/Abstract]) OR ("delayed onset muscle soreness"[Title/Abstract]) OR (DOMS[Title/Abstract]) OR ("muscle soreness"[Title/Abstract]) OR ("muscle damage"[Title/Abstract]) OR ("muscle injury"[Title/Abstract]) OR ("creatine kinase"[Title/Abstract]) OR (CK[Title/Abstract]) OR ("interleukin-6"[Title/Abstract]) OR (IL-6[Title/Abstract]) OR ("C-reactive protein"[Title/Abstract]) OR (CRP[Title/Abstract]) OR (inflammation[Title/Abstract]) OR (fatigue[Title/Abstract]))AND(("cold water immersion"[Title/Abstract]) OR (CWI[Title/Abstract]) OR (cryotherapy[Title/Abstract]) OR (icing[Title/Abstract]) OR ("ice pack"[Title/Abstract]) OR ("cold therapy"[Title/Abstract]) OR ("local cold"[Title/Abstract]) OR ("local cryotherapy"[Title/Abstract]) OR ("contrast water therapy"[Title/Abstract]) OR ("contrast bath"[Title/Abstract]) OR (CWT[Title/Abstract]) OR ("whole-body cryotherapy"[Title/Abstract]) OR (WBC[Title/Abstract]) OR ("active recovery"[Title/Abstract]) OR ("cool-down"[Title/Abstract]) OR (massage[Title/Abstract]) OR ("manual therapy"[Title/Abstract]) OR ("foam rolling"[Title/Abstract]) OR (compression[Title/Abstract]) OR ("compression garment"[Title/Abstract]) OR ("graduated compression"[Title/Abstract]) OR ("compression clothing"[Title/Abstract]) OR (stretch*[Title/Abstract]) OR (vibration[Title/Abstract]) OR ("whole-body vibration"[Title/Abstract]) OR ("electrical stimulation"[Title/Abstract]) OR ("electric stimulation"[Title/Abstract]) OR ("neuromuscular electrical stimulation"[Title/Abstract]) OR (NMES[Title/Abstract]) OR (TENS[Title/Abstract]) OR (electrotherapy[Title/Abstract]) OR (ultrasound[Title/Abstract]) OR (phonophoresis[Title/Abstract]) OR ("shockwave therapy"[Title/Abstract]) OR ("shock wave therapy"[Title/Abstract]) OR ("kinesio taping"[Title/Abstract]) OR ("kinesiology taping"[Title/Abstract]) OR ("elastic therapeutic taping"[Title/Abstract]) OR ("kinesio tape"[Title/Abstract]) OR (phototherapy[Title/Abstract]) OR (photobiomodulation[Title/Abstract]) OR ("low-level laser therapy"[Title/Abstract]) OR (LLLT[Title/Abstract]) OR (PBMT[Title/Abstract]) OR (LED[Title/Abstract]) OR (acupuncture[Title/Abstract]))AND((randomized[Title/Abstract]) OR (randomised[Title/Abstract]) OR (randomly[Title/Abstract]) OR (random*[Title/Abstract]) OR ("randomized controlled trial"[Publication Type]) OR (placebo[Title/Abstract]) OR (sham[Title/Abstract]) OR (trial[Title/Abstract]) OR (crossover[Title/Abstract]) OR ("cross-over"[Title/Abstract])))NOT((animals[MeSH Terms]) NOT (humans[MeSH Terms]))

**Cochrane Library (Results: 408)**

#1: MeSH descriptor: [Adult] explode all trees

#2: MeSH descriptor: [Athletes] explode all trees

#3: MeSH descriptor: [Exercise] explode all trees

#4: MeSH descriptor: [Sports] explode all trees

#5: (adult* or "healthy adult*" or athlete* or sport* or "physically active" or trained or untrained or "recreationally active" or student* or volunteer*):ti,ab,kw

#6: #1 OR #2 OR #3 OR #4 OR #5

#7: MeSH descriptor: [Exercise] explode all trees

#8: MeSH descriptor: [Muscle, Skeletal] explode all trees

#9: ("acute exercise" or post-exercise or postexercise or "after exercise" or "exercise recovery" or "recovery phase" or "exercise-induced muscle damage" or EIMD or "delayed onset muscle soreness" or DOMS or "muscle soreness" or "muscle damage" or "muscle injury" or "exercise-induced muscle soreness" or "exercise induced muscle damage" or "eccentric exercise" or "eccentric contraction*" or "downhill running" or "drop jump*" or "resistance exercise" or "plyometric exercise" or "high-intensity exercise" or "intermittent exercise"):ti,ab,kw

#10: #7 OR #8 OR #9

#11: MeSH descriptor: [Creatine Kinase] explode all trees

#12: MeSH descriptor: [Interleukin-6] explode all trees

#13: MeSH descriptor: [C-Reactive Protein] explode all trees

#14: ("creatine kinase" or CK or "interleukin-6" or IL-6 or "C-reactive protein" or CRP or inflammation or inflammatory or "muscle damage marker*" or "muscle damage biomarker*" or soreness or pain or fatigue):ti,ab,kw

#15: #11 OR #12 OR #13 OR #14

#16: MeSH descriptor: [Cryotherapy] explode all trees

#17: MeSH descriptor: [Massage] explode all trees

#18: MeSH descriptor: [Acupuncture Therapy] explode all trees

#19: MeSH descriptor: [Electric Stimulation Therapy] explode all trees

#20: MeSH descriptor: [Ultrasonic Therapy] explode all trees

#21: MeSH descriptor: [Low-Level Light Therapy] explode all trees

#22: ("cold water immersion" or CWI or cryotherapy or icing or "ice pack" or "cold therapy" or "local cold" or "local cryotherapy" or "contrast water therapy" or "contrast bath" or CWT or "whole-body cryotherapy" or WBC):ti,ab,kw

#23: ("active recovery" or "cool-down" or cooldown or massage or "manual therapy" or "foam rolling" or compression or "compression garment*" or "graduated compression" or "compression clothing"):ti,ab,kw

#24: (stretch* or vibration or "whole-body vibration" or "electrical stimulation" or "electric stimulation" or "neuromuscular electrical stimulation" or NMES or TENS or electrotherapy):ti,ab,kw

#25: (ultrasound or phonophoresis or "shockwave therapy" or "shock wave therapy" or "extracorporeal shockwave" or "extracorporeal shock wave"):ti,ab,kw

#26: ("kinesio tap*" or "kinesiology tap*" or "elastic therapeutic tap*" or "kinesio tape" or "kinesio taping" or "kinesiology taping"):ti,ab,kw

#27: (phototherapy or photobiomodulation or "low-level laser therapy" or LLLT or PBMT or LED or acupuncture):ti,ab,kw

#28: #16 OR #17 OR #18 OR #19 OR #20 OR #21 OR #22 OR #23 OR #24 OR #25 OR #26 OR #27

#29: MeSH descriptor: [Randomized Controlled Trials as Topic] explode all trees

#30: MeSH descriptor: [Cross-Over Studies] explode all trees

#31: (random* or randomized or randomised or randomly or placebo or sham or trial or crossover or "cross-over" or "randomized controlled trial" or "randomised controlled trial" or RCT):ti,ab,kw

#32: #29 OR #30 OR #31

#33: #6 AND #10 AND #15 AND #28 AND #32

**Web of Science(Results: 2205)**

(((((TS=(adult*)) OR TS=("healthy adult*") OR TS=(athlete*) OR TS=(sport*) OR TS=("physically active") OR TS=(trained) OR TS=(untrained) OR TS=("recreationally active") OR TS=(student*) OR TS=(volunteer*))) AND (((TS=("acute exercise")) OR TS=("post-exercise") OR TS=(postexercise) OR TS=("after exercise") OR TS=("exercise recovery") OR TS=("recovery phase") OR TS=("exercise-induced muscle damage") OR TS=("exercise induced muscle damage") OR TS=(EIMD) OR TS=("delayed onset muscle soreness") OR TS=(DOMS) OR TS=("muscle soreness") OR TS=("exercise-induced muscle soreness") OR TS=("muscle damage") OR TS=("muscle injury") OR TS=("eccentric exercise") OR TS=("eccentric contraction*") OR TS=("downhill running") OR TS=("drop jump*") OR TS=("resistance exercise") OR TS=("plyometric exercise") OR TS=("high-intensity exercise") OR TS=("intermittent exercise"))) AND (((TS=("creatine kinase")) OR TS=(CK) OR TS=("interleukin-6") OR TS=("IL-6") OR TS=("C-reactive protein") OR TS=(CRP) OR TS=(inflammation) OR TS=(inflammatory) OR TS=(fatigue) OR TS=(soreness) OR TS=(pain) OR TS=("muscle damage marker*") OR TS=("muscle damage biomarker*"))) AND (((TS=("cold water immersion")) OR TS=(CWI) OR TS=(cryotherapy) OR TS=(icing) OR TS=("ice pack") OR TS=("cold therapy") OR TS=("local cold") OR TS=("local cryotherapy") OR TS=("contrast water therapy") OR TS=("contrast bath") OR TS=(CWT) OR TS=("whole-body cryotherapy") OR TS=(WBC) OR TS=("active recovery") OR TS=("cool-down") OR TS=(cooldown) OR TS=(massage) OR TS=("manual therapy") OR TS=("foam rolling") OR TS=(compression) OR TS=("compression garment*") OR TS=("graduated compression") OR TS=("compression clothing") OR TS=(stretch*) OR TS=(vibration) OR TS=("whole-body vibration") OR TS=("electrical stimulation") OR TS=("electric stimulation") OR TS=("neuromuscular electrical stimulation") OR TS=(NMES) OR TS=(TENS) OR TS=(electrotherapy) OR TS=(ultrasound) OR TS=(phonophoresis) OR TS=("shockwave therapy") OR TS=("shock wave therapy") OR TS=("extracorporeal shockwave") OR TS=("extracorporeal shock wave") OR TS=("kinesio tap*") OR TS=("kinesiology tap*") OR TS=("elastic therapeutic tap*") OR TS=("kinesio tape") OR TS=("kinesio taping") OR TS=("kinesiology taping") OR TS=(phototherapy) OR TS=(photobiomodulation) OR TS=("low-level laser therapy") OR TS=(LLLT) OR TS=(PBMT) OR TS=(LED) OR TS=(acupuncture))) AND (((TS=(random*)) OR TS=(randomized) OR TS=(randomised) OR TS=(randomly) OR TS=(placebo) OR TS=(sham) OR TS=(trial) OR TS=(crossover) OR TS=("cross-over") OR TS=("randomized controlled trial") OR TS=("randomised controlled trial") OR TS=(RCT))))

**Embase (Results: 771)**

#1 adult*:ti,ab,kw OR 'healthy adult*':ti,ab,kw OR athlete*:ti,ab,kw OR sport*:ti,ab,kw OR 'physically active':ti,ab,kw OR trained:ti,ab,kw OR untrained:ti,ab,kw OR 'recreationally active':ti,ab,kw OR student*:ti,ab,kw OR volunteer*:ti,ab,kw OR 'adult'/exp OR 'athlete'/exp OR 'sportsman'/exp

#2 'acute exercise':ti,ab,kw OR 'post-exercise':ti,ab,kw OR postexercise:ti,ab,kw OR 'after exercise':ti,ab,kw OR 'exercise recovery':ti,ab,kw OR 'recovery phase':ti,ab,kw OR 'exercise-induced muscle damage':ti,ab,kw OR 'exercise induced muscle damage':ti,ab,kw OR eimd:ti,ab,kw OR 'delayed onset muscle soreness':ti,ab,kw OR doms:ti,ab,kw OR 'muscle soreness':ti,ab,kw OR 'exercise-induced muscle soreness':ti,ab,kw OR 'muscle damage':ti,ab,kw OR 'muscle injury':ti,ab,kw OR 'eccentric exercise':ti,ab,kw OR 'eccentric contraction*':ti,ab,kw OR 'downhill running':ti,ab,kw OR 'drop jump*':ti,ab,kw OR 'resistance exercise':ti,ab,kw OR 'plyometric exercise':ti,ab,kw OR 'high-intensity exercise':ti,ab,kw OR 'intermittent exercise':ti,ab,kw OR 'exercise'/exp OR 'muscle pain'/exp OR 'muscle injury'/exp

#3 'creatine kinase':ti,ab,kw OR ck:ti,ab,kw OR 'interleukin-6':ti,ab,kw OR 'il-6':ti,ab,kw OR 'c-reactive protein':ti,ab,kw OR crp:ti,ab,kw OR inflammation:ti,ab,kw OR inflammatory:ti,ab,kw OR fatigue:ti,ab,kw OR soreness:ti,ab,kw OR pain:ti,ab,kw OR 'muscle damage marker*':ti,ab,kw OR 'muscle damage biomarker*':ti,ab,kw OR 'creatine kinase'/exp OR 'interleukin 6'/exp OR 'c reactive protein'/exp OR 'inflammation'/exp

#4 'cold water immersion':ti,ab,kw OR cwi:ti,ab,kw OR cryotherapy:ti,ab,kw OR icing:ti,ab,kw OR 'ice pack':ti,ab,kw OR 'cold therapy':ti,ab,kw OR 'local cold':ti,ab,kw OR 'local cryotherapy':ti,ab,kw OR 'contrast water therapy':ti,ab,kw OR 'contrast bath':ti,ab,kw OR cwt:ti,ab,kw OR 'whole-body cryotherapy':ti,ab,kw OR wbc:ti,ab,kw OR 'active recovery':ti,ab,kw OR 'cool-down':ti,ab,kw OR cooldown:ti,ab,kw OR massage:ti,ab,kw OR 'manual therapy':ti,ab,kw OR 'foam rolling':ti,ab,kw OR compression:ti,ab,kw OR 'compression garment*':ti,ab,kw OR 'graduated compression':ti,ab,kw OR 'compression clothing':ti,ab,kw OR stretch*:ti,ab,kw OR vibration:ti,ab,kw OR 'whole-body vibration':ti,ab,kw OR 'electrical stimulation':ti,ab,kw OR 'electric stimulation':ti,ab,kw OR 'neuromuscular electrical stimulation':ti,ab,kw OR nmes:ti,ab,kw OR tens:ti,ab,kw OR electrotherapy:ti,ab,kw OR ultrasound:ti,ab,kw OR phonophoresis:ti,ab,kw OR 'shockwave therapy':ti,ab,kw OR 'shock wave therapy':ti,ab,kw OR 'extracorporeal shockwave':ti,ab,kw OR 'extracorporeal shock wave':ti,ab,kw OR 'kinesio tap*':ti,ab,kw OR 'kinesiology tap*':ti,ab,kw OR 'elastic therapeutic tap*':ti,ab,kw OR 'kinesio tape':ti,ab,kw OR 'kinesio taping':ti,ab,kw OR 'kinesiology taping':ti,ab,kw OR phototherapy:ti,ab,kw OR photobiomodulation:ti,ab,kw OR 'low-level laser therapy':ti,ab,kw OR lllt:ti,ab,kw OR pbmt:ti,ab,kw OR led:ti,ab,kw OR acupuncture:ti,ab,kw OR 'cryotherapy'/exp OR 'massage'/exp OR 'compression therapy'/exp OR 'electrostimulation therapy'/exp OR 'ultrasound therapy'/exp OR 'low level laser therapy'/exp OR 'acupuncture'/exp

#5 random*:ti,ab,kw OR randomized:ti,ab,kw OR randomised:ti,ab,kw OR randomly:ti,ab,kw OR placebo:ti,ab,kw OR sham:ti,ab,kw OR trial:ti,ab,kw OR crossover:ti,ab,kw OR 'cross-over':ti,ab,kw OR 'randomized controlled trial':ti,ab,kw OR 'randomised controlled trial':ti,ab,kw OR rct:ti,ab,kw OR 'randomized controlled trial'/exp OR 'crossover procedure'/exp

#6 #1 AND #2 AND #3 AND #4 AND #5

# 2. Eligibility criteria adopted by the PICOS framework

Supplementary Table 2. Inclusion criteria adopted by the PICOS framework.

| **Definition** | **Inclusion criteria applied** |
| --- | --- |
| ****Population**** | Healthy adults, physically active individuals, recreationally trained participants, university students, amateur athletes, or trained athletes of any sex who underwent exercise-induced muscle damage or delayed onset muscle soreness protocols. |
| ****Intervention**** | Post-exercise recovery or regeneration interventions aimed at reducing delayed onset muscle soreness or exercise-induced muscle damage, including cold-water immersion, contrast-water therapy, local cryotherapy, whole-body cryotherapy, compression garments, kinesio taping, vibration therapy, electrical stimulation/electrotherapy, stretching, massage/manual therapy, acupuncture, laser-only low-level laser therapy (LLLT) and LED-containing photobiomodulation regimens (PHO), shockwave therapy, phonophoresis, or other clearly defined recovery modalities. |
| ****Control**** | Passive recovery, usual recovery, no treatment, placebo/sham intervention, or other eligible active recovery intervention. |
| ****Outcome**** | Delayed onset muscle soreness, creatine kinase, and/or interleukin-6 measured at 24 h and/or 48 h after exercise. Studies were included when means, standard deviations, and sample sizes were available or could be calculated from reported data. |
| ****Study design**** | Randomized controlled trials, including parallel-group randomized controlled trials and randomized crossover trials. |

# 3. Characteristics of included studies

| Supplementary Table 3. Characteristics of included studies. | | | | | | | | | | | |
| --- | --- | --- | --- | --- | --- | --- | --- | --- | --- | --- | --- |
| Study | Participants (exercise vs control) | | | Interventions | | | | | Study  design | Analysis time points | Outcomes |
|  | Sample size | Age | Sex | Population | Induction method | Type | Comparator | Intervention site |  |  |  |
| Antonialli  2014 | 10 vs 10 | 24.10 ± 1.52 | Male | healthy untrained men | eccentric quadriceps exercise | PHO | CON/PBO | Quadriceps femoris | RCT | 24 h, 48 h | CK, DOMS |
| Boobphachart  2017 | 17 vs 17 vs 17 | 41.7 ± 8.6 | Female | healthy untrained women | eccentric quadriceps exercise | KT; STR | CON/PBO | Quadriceps femoris | RCT | 24 h, 48 h | CK |
| Cheng 2022 | 13 vs 12 | 18.5 ± 2.9  vs  19.3 ± 4.2 | Male | field athletes | downhill running | VIB | CON/PBO | Quadriceps femoris | RCT | 24 h, 48 h | CK, DOMS, IL-6 |
| Cheng 2025 | 11 vs 11 | 20.3 ± 1.5  vs  20.0 ± 1.2 | 13 women/9 men | field athletes | downhill running | VIB | CON/PBO | Quadriceps femoris | RCT | 24 h, 48 h | CK, DOMS, IL-6 |
| De Marchi 2017 | 8 vs 8 vs 8 | 25.30 ± 3.32 | Male | healthy physically active men | eccentric exercise | CRY; PHO | CON/PBO | Upper arm | RCT | 24 h, 48 h | CK |
| Duffield 2010 | 11 | 20.9 ± 2.7 | NR | trained team-sport athletes | intermittent sprint and plyometric exercise | COMP | CON/PBO | Lower body | crossover RCT | 24 h | CK |
| Fonseca 2016 | 8 | 24.0 ± 3.6 | Male | healthy athletes | jiu-jitsu training session | CWI | CON/PBO | Whole body immersion to the neck | crossover RCT | 24 h, 48 h | CK |
| Guilhem 2013 | 12 vs 12 | 23.9 ± 1.4  vs  25.2 ± 1.1 | Male | healthy adults | isokinetic eccentric contractions of the elbow flexors | CRY | CON/PBO | elbow flexors | RCT | 24 h, 48 h | CK, DOMS, IL-6 |
| Kim 2017 | 8 vs 8 | 23.13 ± 3.76  vs  24.25 ± 1.28 | Male | healthy university students | isokinetic eccentric contractions of the elbow flexors | COMP | CON/PBO | elbow flexors | RCT | 24 h, 48 h | CK, DOMS |
| Machado 2020 | 10 vs 10 | 26.83 ± 6.02 | Male | healthy adults | eccentric quadriceps exercise | PHO | CON/PBO | Quadriceps femoris | RCT | 24 h, 48 h | CK, DOMS |
| Oakley 2013 | 20 vs 10 | 24.0 ± 4.0 | 16 women/17 men enrolled | healthy trained and untrained adults | eccentric quadriceps exercise | CRY | CON/PBO | Quadriceps femoris | RCT | 24 h, 48 h | CK |
| Pruscino 2013 | 8 | 21.9 ± 2.3 | Male | highly trained field hockey players | LIST hockey simulation exercise protocol | COMP | CON/PBO | Systemic muscle | crossover RCT | 24 h, 48 h | CK, IL-6 |
| Qu 2020 | 12 | 21.00 ± 0.95 | Male | middle- and long-distance runners | 90-min treadmill running | CWI; CWT; WBC | CON/PBO | Whole body / lower limbs | crossover RCT | 24 h, 48 h | CK, DOMS |
| Eston  1999 | 8 vs 7 | 22.0 ± 2.0 | Female | students | eccentric exercise of the elbow flexors | CRY | CON/PBO | elbow flexors | RCT | 24 h, 48 h | CK |
| Torres  2013 | 14 vs 14 | 21.4 ± 1.9 | Male | healthy untrained men | eccentric quadriceps exercise | STR | CON/PBO | Quadriceps femoris | RCT | 24 h, 48 h | CK, DOMS |
| Wei 2025 | 5 vs 5 vs 5 vs 5 vs 5 vs 5 | 21.1 ± 1.4 | Male | healthy university students | walking deep-squat jumps and stationary half-squat jumps | CWI; ES; STR; VIB; MAS | CON/PBO | Quadriceps femoris | RCT | 24 h, 48 h | CK, IL-6 |
| de Paiva 2016 | 10 vs 10 | 24.98 ± 5.90 | Male | healthy untrained men | eccentric isokinetic contractions of knee extensors | PHO; CRY | CON/PBO | Quadriceps femoris | RCT | 24 h, 48 h | CK, DOMS |
| Hazar Kanik 2019 | 27 vs 27 | 22.5 ± 2.0  vs  23.0 ± 2.0 | 28/26 | healthy nonathletic adults | 100 consecutive drop-jumps from a 0.60 m high platform | KT | CON/PBO | Quadriceps femoris | RCT | 48 h | CK, DOMS |
| Douris 2006 | 9 vs 9 | 24.1 ± 3.0 | NR | healthy adults | eccentric exercise of non-dominant elbow flexors | PHO | CON/PBO | elbow flexors | RCT | 24 h, 48 h | DOMS |
| Fleckenstein 2016 | 12 vs 12 | 23.6 ± 2.8 | 14 women/10 men | healthy adults | biceps curls with dumbbell | AC | CON/PBO | elbow flexors | RCT | 24 h, 48 h | DOMS |
| Gatterer 2015 | 7 | 25.6 ± 2.2 | 3 women/4 men | sport science students | downhill running | ES | CON/PBO | Lower limb muscles | crossover RCT | 24 h, 48 h | DOMS |
| Itoh 2008 | 7 vs 10 | NR | 13 women/17 men enrolled | healthy student volunteers | biceps curls with dumbbell | AC | CON/PBO | elbow flexors | RCT | 24 h, 48 h | DOMS |
| Kirmizigil 2020 | 22 | 21.36 ± 1.68 | Male | healthy amateur athletes | drop jumps | KT | CON/PBO | Rectus femoris | crossover RCT | 24 h, 48 h | DOMS |
| Lee 2015 | 18 vs 19 | 23.5 ± 1.2  vs  22.5 ± 1.4 | Male | healthy adults | eccentric exercise of non-dominant biceps brachii | KT | CON/PBO | Biceps brachii | RCT | 24 h, 48 h | DOMS |
| McLoughlin 2004 | 14 | 23.9 ± 4.6 | Male | healthy adults | repetitive eccentric contractions of elbow flexors | ES | CON/PBO | Elbow flexors | crossover RCT | 24 h, 48 h | DOMS |
| Mikhail Santos Cerqueira 2015 | 7 vs 6 | 22 ± 2.65  vs  20 ± 2.45 | Male | healthy adults | eccentric exercise of non-dominant elbow flexors | COMP | CON/PBO | elbow flexors | RCT | 24 h, 48 h | DOMS |
| Szymura 2016 | 10 | 21.2 ± 1.33 | Male | healthy adults | downhill running | KT | CON/PBO | Lower limbs | crossover RCT | 24 h, 48 h | DOMS |
| Xue 2023 | 8 vs 8 vs 8 | 19.75 ± 1.59 | Male | healthy college students without regular exercise training | 5 sets × 20 drop jumps from 0.6 m box | KT; COMP | CON/PBO | Lower limbs | RCT | 24 h, 48 h | DOMS, IL-6 |
| Krueger 2019 | 11 | 25.9 ± 2.1 | Male | healthy athletes | High-intensity intermittent running | WBC | CON/PBO | Whole body | crossover RCT | 24 h | IL-6 |
| Kay  2018 | 13 vs 13 | 27.8 ± 8 | 10 women/16 men | recreationally active adults | downhill run | STR | CON/PBO | Knee extensors | RCT | 24 h | CK |
| Vanin  2016 | 7 vs7 | 18.81 ± 0.80 | Male | high-level soccer players | 75 eccentric isokinetic contractions of knee extensors | LLLT | CON/PBO | Knee extensors | RCT | 24 h, 48 h | CK, DOMS, IL-6 |
| Xia  2022 | 8 vs 8 | 24.12 ± 1.03 | Male | healthy college students | High-intensity exercise | LIFU | CON/PBO | Quadriceps femoris and calf muscles | RCT | 24 h, 48 h | CK, DOMS |
| Sudhakar 2023 | 16 vs 16 | 20.4 ± 2.1  vs  21.1 ± 2.8 | Male | novice athletes | Eccentric loading of non-dominant elbow | SW | CON/PBO | Non-dominant biceps brachii | RCT | 24 h, 48 h | CK |
| Pinto  2022 | 12 | 27 ± 5.54 | Male | amateur CrossFit® athletes | CrossFit® | PHO | CON/PBO | Bilateral lower limbs | crossover RCT | 24 h, 48 h | CK, IL-6 |
| Baroni  2010 | 18 vs 18 | 25.35 ± 3.41  vs  24.28 ± 5.48 | Male | healthy physically active students | 75 maximal knee extensor eccentric contractions | LLLT | CON/PBO | Quadriceps femoris | RCT | 24 h, 48 h | CK, DOMS |

Note. AC, acupuncture; CK, creatine kinase; COMP, compression therapy; CON/PBO, combined control/placebo condition; CRY, local cryotherapy; CWI, cold-water immersion; CWT, contrast-water therapy; DOMS, delayed-onset muscle soreness; ES, electrical stimulation/electrotherapy; IL-6, interleukin-6; KT, kinesiology taping; LIFU, low-intensity focused ultrasound; LLLT, laser-only low-level laser therapy; MAS, massage; NR, not reported; PHO, LED-containing photobiomodulation regimens; RCT, randomized controlled trial; STR, stretching; SW, shock wave therapy; VIB, vibration therapy; WBC, whole-body cryotherapy.

**Supplementary Table 3A. Operational classification and protocol characteristics of light-based interventions.**

LLLT denotes laser-only treatment. PHO denotes LED-containing photobiomodulation regimens, including diode-based phototherapy and combined laser-LED protocols. The categories distinguish the delivered light-source configuration; additional components are reported below.

| **Study** | **Node** | **Light sources and additional components** | **Region and exercise model** | **Participants** |
| --- | --- | --- | --- | --- |
| Baroni 2010 | LLLT | 810-nm laser only; five laser diodes. | Quadriceps; 75 maximal eccentric knee-extensor contractions. | Healthy physically active men. |
| Vanin 2016 | LLLT | 810-nm laser only; five laser diodes; multiple dose groups. | Quadriceps; 75 eccentric isokinetic knee-extensor contractions. | High-level male soccer athletes. |
| Douris 2006 | PHO | 660-nm visible diodes and 880-nm infrared superluminous diodes. | Biceps brachii; eccentric elbow-flexor exercise. | Healthy adults. |
| De Marchi 2017 | PHO | 660-nm red and 850-nm infrared LEDs. | Biceps brachii; five sets of ten eccentric/concentric elbow-flexor contractions. | Healthy physically active men. |
| Antonialli 2014 | PHO | 905-nm super-pulsed laser plus red and 875-nm infrared LEDs; 35-mT static magnetic field (see note). | Quadriceps; 75 eccentric isokinetic knee-extensor contractions. | Healthy untrained men. |
| de Paiva 2016 | PHO | 905-nm super-pulsed laser plus 640/875-nm LEDs; 35-mT static magnetic field. | Quadriceps; 75 eccentric isokinetic knee-extensor contractions. | Healthy untrained men. |
| Machado 2020 | PHO | 905-nm laser plus 640/875-nm LEDs; 35-mT static magnetic field. | Quadriceps; 75 eccentric knee-extensor contractions; local and non-local irradiation arms. | Sedentary men. |
| Pinto 2022 | PHO | 905-nm laser plus 633/850-nm LEDs; 110-mT static magnetic field. | Bilateral lower limbs; CrossFit workout. | Male amateur CrossFit athletes. |

# **4. Risk of bias**

4.1 Risk of bias in randomized controlled trials

| Supplementary Table 4. Risk-of-bias assessment for randomized controlled trials. | | | | | | |
| --- | --- | --- | --- | --- | --- | --- |
| **Study** | **Randomization**  **process** | **Deviations from the intended intervention** | **Missing**  **outcome data** | **Measurement of the outcome** | **Selection of the reported results** | **Overall Bias** |
| Antonialli 2014 | Low | Low | Low | Low | Some concerns | Some concerns |
| Boobphachart 2017 | Some concerns | Some concerns | Low | Some concerns | Some concerns | Some concerns |
| Cheng  2022 | Some concerns | Some concerns | Low | High | Some concerns | High |
| Cheng  2025 | Some concerns | Some concerns | Low | High | Some concerns | High |
| De Marchi 2017 | Some concerns | Some concerns | Low | Low | Some concerns | Some concerns |
| Guilhem  2013 | Some concerns | Some concerns | Low | High | Some concerns | High |
| Kim  2017 | Some concerns | Some concerns | Low | High | Some concerns | High |
| Machado  2020 | Low | Low | Low | Low | Low | Low |
| Oakley  2013 | Some concerns | Some concerns | Some concerns | Low | Some concerns | Some concerns |
| Eston  1999 | Some concerns | Some concerns | Low | Low | Some concerns | Some concerns |
| Torres  2013 | Some concerns | Some concerns | Low | High | Some concerns | High |
| Wei  2025 | Low | Some concerns | Low | Low | Some concerns | Some concerns |
| de Paiva  2016 | Low | Some concerns | Low | High | Some concerns | High |
| Hazar Kanik 2019 | Some concerns | Some concerns | Low | High | Some concerns | High |
| Douris  2006 | Some concerns | Some concerns | Low | Some concerns | Some concerns | Some concerns |
| Fleckenstein 2016 | Low | Some concerns | Low | Some concerns | Some concerns | Some concerns |
| Itoh  2008 | Some concerns | Some concerns | High | High | Some concerns | High |
| Lee  2015 | Some concerns | Some concerns | High | High | Some concerns | High |
| Mikhail Santos Cerqueira 2015 | Some concerns | Some concerns | Low | High | Some concerns | High |
| Xue  2023 | Low | Some concerns | Low | High | Some concerns | High |
| Kay  2018 | Some concerns | Some concerns | Low | Low | Some concerns | Some concerns |
| Vanin  2016 | Low | Low | Low | Low | Some concerns | Some concerns |
| Xia  2022 | Some concerns | Some concerns | Low | High | Some concerns | High |
| Sudhakar  2023 | Some concerns | Some concerns | Low | Low | Some concerns | Some concerns |
| Baroni  2010 | Some concerns | Low | Low | Low | Some concerns | Some concerns |

4.2 Risk of bias in randomized crossover trials

| Supplementary Table 5. Risk-of-bias assessment for randomized crossover trials. | | | | | | | |
| --- | --- | --- | --- | --- | --- | --- | --- |
| **Study** | **Randomization**  **process** | **Period and carryover effects** | **Deviations from intended intervention** | **Missing outcome data** | **Measurement of the outcome** | **Selection of reported results** | **Overall Bias** |
| Duffield 2010 | Some concerns | Some concerns | Some concerns | Low | Low | Some concerns | Some concerns |
| Fonseca 2016 | Some concerns | Some concerns | Some concerns | High | Low | Some concerns | High |
| Pruscino 2013 | Some concerns | Some concerns | Some concerns | Low | Low | Some concerns | Some concerns |
| Qu  2020 | High | High | Some concerns | Low | High | Some concerns | High |
| Gatterer 2015 | Some concerns | Some concerns | Some concerns | Some concerns | High | Some concerns | High |
| Kirmizigil 2020 | Some concerns | Some concerns | Some concerns | Some concerns | High | Some concerns | High |
| McLoughlin 2004 | Some concerns | Some concerns | Some concerns | Low | High | Some concerns | High |
| Szymura 2016 | Some concerns | Some concerns | Some concerns | Low | High | Some concerns | High |
| Malte Krueger 2019 | Some concerns | Low | Some concerns | Low | Low | Some concerns | Some concerns |
| Pinto  2022 | Low | Some concerns | Low | Low | Low | Low | Some concerns |

# 5. Network meta-analysis

5.1 League tables

Supplementary Table 6. League table for DOMS at 24 h.

| AC | -8 [-33.8, 20.41] | 2.13 [-18.56, 24.98] | -6.53 [-34.62, 23.72] | -6.76 [-42.86, 31.17] | -24.76 [-60.85, 13.03] | -13.41 [-44.22, 19.43] | -4.11 [-28.52, 23.17] | -32.48 [-66.48, 4.12] | -5.08 [-36.2, 28.54] | -16.03 [-41.17, 11.15] | 2.18 [-32.51, 38.93] | -2.53 [-30.75, 28.36] | -36.82 [-72.88, 1.21] |
| --- | --- | --- | --- | --- | --- | --- | --- | --- | --- | --- | --- | --- | --- |
| 8 [-20.41, 33.8] | COMP | 10.03 [-5.87, 26.34] | 1.48 [-24.13, 26.57] | 1.1 [-32.61, 34.95] | -16.88 [-50.81, 16.76] | -5.5 [-33.79, 22.91] | 3.81 [-15.21, 23.5] | -24.52 [-56.52, 7.79] | 2.8 [-25.91, 32.23] | -8.15 [-29.94, 13.4] | 10.03 [-22.37, 42.79] | 5.41 [-20.33, 31.56] | -28.96 [-62.75, 5.12] |
| -2.13 [-24.98, 18.56] | -10.03 [-26.34, 5.87] | CON/PBO | -8.59 [-28.4, 10.72] | -8.92 [-38.92, 20.66] | -26.92 [-56.89, 2.58] | -15.55 [-38.87, 7.64] | -6.2 [-20.06, 7.91] | **-34.54 [-62.45, -6.61]** | -7.21 [-30.97, 17.01] | **-18.2 [-32.91, -3.72]** | -0.03 [-28.4, 28.26] | -4.69 [-24.63, 15.73] | **-38.92 [-69.08, -9.15]** |
| 6.53 [-23.72, 34.62] | -1.48 [-26.57, 24.13] | 8.59 [-10.72, 28.4] | CRY | -0.26 [-35.7, 35.31] | -18.32 [-53.8, 17.42] | -6.99 [-37, 23.64] | 2.41 [-21.24, 26.66] | -25.94 [-59.72, 8.46] | 1.33 [-29.42, 32.73] | -9.58 [-31.26, 12.16] | 8.54 [-25.93, 43.46] | 4.01 [-23.82, 32.37] | -30.3 [-66.25, 5.37] |
| 6.76 [-31.17, 42.86] | -1.1 [-34.95, 32.61] | 8.92 [-20.66, 38.92] | 0.26 [-35.31, 35.7] | CWI | -18.06 [-47.58, 11.55] | -6.6 [-44.37, 31.29] | 2.64 [-29.92, 35.95] | -25.71 [-66.16, 15.22] | 1.69 [-36.06, 40.32] | -9.26 [-42.34, 23.99] | 8.93 [-32.12, 50.11] | 4.26 [-31.65, 40.33] | **-30.01 [-59.92, -0.3]** |
| 24.76 [-13.03, 60.85] | 16.88 [-16.76, 50.81] | 26.92 [-2.58, 56.89] | 18.32 [-17.42, 53.8] | 18.06 [-11.55, 47.58] | CWT | 11.47 [-26.71, 49.26] | 20.76 [-11.8, 53.68] | -7.64 [-48.22, 33.51] | 19.68 [-17.9, 58.41] | 8.67 [-24.24, 41.97] | 26.96 [-14.58, 68.04] | 22.3 [-13.6, 58.49] | -12 [-41.83, 17.82] |
| 13.41 [-19.43, 44.22] | 5.5 [-22.91, 33.79] | 15.55 [-7.64, 38.87] | 6.99 [-23.64, 37] | 6.6 [-31.29, 44.37] | -11.47 [-49.26, 26.71] | ES | 9.39 [-17.47, 36.45] | -19.04 [-55.14, 17.57] | 8.31 [-24.85, 42.06] | -2.66 [-30.05, 24.59] | 15.45 [-21.4, 52.12] | 10.87 [-19.54, 41.89] | -23.46 [-61.44, 14.66] |
| 4.11 [-23.17, 28.52] | -3.81 [-23.5, 15.21] | 6.2 [-7.91, 20.06] | -2.41 [-26.66, 21.24] | -2.64 [-35.95, 29.92] | -20.76 [-53.68, 11.8] | -9.39 [-36.45, 17.47] | KT | -28.41 [-59.84, 2.57] | -1.07 [-28.75, 26.84] | -11.94 [-32.5, 7.96] | 6.24 [-25.7, 37.68] | 1.54 [-23.1, 25.92] | **-32.74 [-65.9, -0.19]** |
| 32.48 [-4.12, 66.48] | 24.52 [-7.79, 56.52] | **34.54 [6.61, 62.45]** | 25.94 [-8.46, 59.72] | 25.71 [-15.22, 66.16] | 7.64 [-33.51, 48.22] | 19.04 [-17.57, 55.14] | 28.41 [-2.57, 59.84] | LIFU | 27.4 [-9.27, 64.68] | 16.36 [-15.17, 47.76] | 34.54 [-5.15, 74.42] | 29.91 [-4.48, 64.68] | -4.3 [-45.41, 36.52] |
| 5.08 [-28.54, 36.2] | -2.8 [-32.23, 25.91] | 7.21 [-17.01, 30.97] | -1.33 [-32.73, 29.42] | -1.69 [-40.32, 36.06] | -19.68 [-58.41, 17.9] | -8.31 [-42.06, 24.85] | 1.07 [-26.84, 28.75] | -27.4 [-64.68, 9.27] | LLLT | -10.95 [-39.55, 16.86] | 7.22 [-30.27, 44.39] | 2.55 [-28.98, 33.74] | -31.75 [-70.48, 6.14] |
| 16.03 [-11.15, 41.17] | 8.15 [-13.4, 29.94] | **18.2 [3.72, 32.91]** | 9.58 [-12.16, 31.26] | 9.26 [-23.99, 42.34] | -8.67 [-41.97, 24.24] | 2.66 [-24.59, 30.05] | 11.94 [-7.96, 32.5] | -16.36 [-47.76, 15.17] | 10.95 [-16.86, 39.55] | PHO | 18.27 [-13.56, 50.1] | 13.54 [-11.15, 38.7] | -20.74 [-54.43, 12.49] |
| -2.18 [-38.93, 32.51] | -10.03 [-42.79, 22.37] | 0.03 [-28.26, 28.4] | -8.54 [-43.46, 25.93] | -8.93 [-50.11, 32.12] | -26.96 [-68.04, 14.58] | -15.45 [-52.12, 21.4] | -6.24 [-37.68, 25.7] | -34.54 [-74.42, 5.15] | -7.22 [-44.39, 30.27] | -18.27 [-50.1, 13.56] | STR | -4.57 [-39.16, 30.37] | -39.04 [-80.19, 2.17] |
| 2.53 [-28.36, 30.75] | -5.41 [-31.56, 20.33] | 4.69 [-15.73, 24.63] | -4.01 [-32.37, 23.82] | -4.26 [-40.33, 31.65] | -22.3 [-58.49, 13.6] | -10.87 [-41.89, 19.54] | -1.54 [-25.92, 23.1] | -29.91 [-64.68, 4.48] | -2.55 [-33.74, 28.98] | -13.54 [-38.7, 11.15] | 4.57 [-30.37, 39.16] | VIB | -34.39 [-70.6, 1.53] |
| 36.82 [-1.21, 72.88] | 28.96 [-5.12, 62.75] | **38.92 [9.15, 69.08]** | 30.3 [-5.37, 66.25] | **30.01 [0.3, 59.92]** | 12 [-17.82, 41.83] | 23.46 [-14.66, 61.44] | **32.74 [0.19, 65.9]** | 4.3 [-36.52, 45.41] | 31.75 [-6.14, 70.48] | 20.74 [-12.49, 54.43] | 39.04 [-2.17, 80.19] | 34.39 [-1.53, 70.6] | WBC |
|  | | | | | | | | | | | | | |
|  | 95% CrI excludes 0; favors row | | |  | Trend favors row treatment | | |  | Trend favors column treatment | | |  | 95% CrI excludes 0; favors column |

Note. Data are presented as mean differences (MDs) with 95% credible intervals (95% CrIs). For DOMS at 24 h after exercise, an MD greater than 0 favors the row-defining intervention, whereas an MD less than 0 favors the column-defining intervention. Network meta-analysis estimates are presented in the lower-left half of the table, and pairwise meta-analysis estimates, where available, are presented in the upper-right half. Values in bold indicate estimates for which the 95% CrI excludes 0.

Supplementary Table 7. League table for DOMS at 48 h.

| AC | 1.55 [-23.67, 27.92] | 4.88 [-14.81, 26.46] | -5.07 [-32.02, 23.47] | -3.26 [-36.89, 32.25] | -23.19 [-57.06, 12.45] | -8.86 [-39.43, 23.92] | 0.18 [-22.6, 25.11] | **-60.96 [-93.43, -26.54]** | 2.69 [-28.58, 36.08] | -14.56 [-38.83, 10.64] | -0.22 [-33.87, 35.76] | -1.69 [-28.27, 27.11] | -35.14 [-68.64, 0.21] |
| --- | --- | --- | --- | --- | --- | --- | --- | --- | --- | --- | --- | --- | --- |
| -1.55 [-27.92, 23.67] | COMP | 3.31 [-12.08, 19.38] | -6.52 [-30.87, 17.83] | -4.73 [-36.58, 27.84] | -24.73 [-56.82, 7.77] | -10.29 [-38.58, 18.39] | -1.31 [-18.89, 17.23] | **-62.52 [-92.82, -31.59]** | 1.14 [-27.5, 31.14] | -16.14 [-36.86, 4.75] | -1.71 [-33.42, 30.84] | -3.26 [-26.96, 21.5] | **-36.69 [-68.4, -4.36]** |
| -4.88 [-26.46, 14.81] | -3.31 [-19.38, 12.08] | CON/PBO | -9.88 [-28.93, 8.48] | -8.06 [-35.87, 19.7] | **-28.08 [-56.27, -0.26]** | -13.64 [-37.64, 10.22] | -4.63 [-16.49, 7.36] | **-65.81 [-92.3, -39.66]** | -2.16 [-26.49, 22.86] | **-19.46 [-33.55, -5.99]** | -5.1 [-32.95, 23.22] | -6.56 [-24.97, 12.16] | **-40.03 [-67.74, -12.44]** |
| 5.07 [-23.47, 32.02] | 6.52 [-17.83, 30.87] | 9.88 [-8.48, 28.93] | CRY | 1.77 [-31.51, 35.76] | -18.22 [-51.65, 15.77] | -3.79 [-33.62, 27.12] | 5.22 [-16.6, 27.83] | **-55.96 [-88.14, -23.31]** | 7.63 [-22.77, 39.47] | -9.58 [-29.86, 11.05] | 4.78 [-28.45, 39.18] | 3.29 [-22.65, 30.17] | -30.13 [-63.46, 3.74] |
| 3.26 [-32.25, 36.89] | 4.73 [-27.84, 36.58] | 8.06 [-19.7, 35.87] | -1.77 [-35.76, 31.51] | CWI | -20.05 [-47.71, 7.79] | -5.59 [-42.22, 31.17] | 3.43 [-26.66, 33.93] | **-57.75 [-96.04, -19.33]** | 5.75 [-30.55, 43.57] | -11.35 [-42.79, 19.47] | 3.04 [-36.26, 42.35] | 1.55 [-31.81, 34.9] | **-31.96 [-59.42, -4.47]** |
| 23.19 [-12.45, 57.06] | 24.73 [-7.77, 56.82] | **28.08 [0.26, 56.27]** | 18.22 [-15.77, 51.65] | 20.05 [-7.79, 47.71] | CWT | 14.42 [-22.12, 51.59] | 23.45 [-6.75, 54.23] | -37.75 [-76.28, 0.85] | 25.88 [-10.95, 63.42] | 8.69 [-22.82, 39.75] | 23.08 [-16.4, 62.68] | 21.61 [-11.97, 55.45] | -11.9 [-39.6, 15.76] |
| 8.86 [-23.92, 39.43] | 10.29 [-18.39, 38.58] | 13.64 [-10.22, 37.64] | 3.79 [-27.12, 33.62] | 5.59 [-31.17, 42.22] | -14.42 [-51.59, 22.12] | ES | 9.05 [-17.73, 35.73] | **-52.2 [-87.67, -17.2]** | 11.44 [-22.76, 46.18] | -5.78 [-33.68, 21.41] | 8.62 [-28.14, 45.22] | 7.13 [-22.95, 37.4] | -26.3 [-63.31, 9.97] |
| -0.18 [-25.11, 22.6] | 1.31 [-17.23, 18.89] | 4.63 [-7.36, 16.49] | -5.22 [-27.83, 16.6] | -3.43 [-33.93, 26.66] | -23.45 [-54.23, 6.75] | -9.05 [-35.73, 17.73] | KT | **-61.18 [-90.34, -32.46]** | 2.45 [-24.61, 30.35] | -14.83 [-33.46, 3.03] | -0.39 [-30.83, 30.16] | -1.96 [-24.03, 20.08] | **-35.38 [-65.9, -5.53]** |
| **60.96 [26.54, 93.43]** | **62.52 [31.59, 92.82]** | **65.81 [39.66, 92.3]** | **55.96 [23.31, 88.14]** | **57.75 [19.33, 96.04]** | 37.75 [-0.85, 76.28] | **52.2 [17.2, 87.67]** | **61.18 [32.46, 90.34]** | LIFU | **63.65 [27.99, 100.09]** | **46.38 [16.23, 75.92]** | **60.74 [22.53, 99.29]** | **59.31 [27.33, 91.57]** | 25.79 [-12.49, 64.34] |
| -2.69 [-36.08, 28.58] | -1.14 [-31.14, 27.5] | 2.16 [-22.86, 26.49] | -7.63 [-39.47, 22.77] | -5.75 [-43.57, 30.55] | -25.88 [-63.42, 10.95] | -11.44 [-46.18, 22.76] | -2.45 [-30.35, 24.61] | **-63.65 [-100.09, -27.99]** | LLLT | -17.25 [-46.3, 10.32] | -2.84 [-40.73, 34.15] | -4.26 [-35.67, 26] | **-37.85 [-75.1, -1.17]** |
| 14.56 [-10.64, 38.83] | 16.14 [-4.75, 36.86] | **19.46 [5.99, 33.55]** | 9.58 [-11.05, 29.86] | 11.35 [-19.47, 42.79] | -8.69 [-39.75, 22.82] | 5.78 [-21.41, 33.68] | 14.83 [-3.03, 33.46] | **-46.38 [-75.92, -16.23]** | 17.25 [-10.32, 46.3] | PHO | 14.38 [-16.51, 46.1] | 12.94 [-9.79, 36.56] | -20.56 [-51.46, 10.84] |
| 0.22 [-35.76, 33.87] | 1.71 [-30.84, 33.42] | 5.1 [-23.22, 32.95] | -4.78 [-39.18, 28.45] | -3.04 [-42.35, 36.26] | -23.08 [-62.68, 16.4] | -8.62 [-45.22, 28.14] | 0.39 [-30.16, 30.83] | **-60.74 [-99.29, -22.53]** | 2.84 [-34.15, 40.73] | -14.38 [-46.1, 16.51] | STR | -1.47 [-35.43, 32] | -35.01 [-74.57, 4.55] |
| 1.69 [-27.11, 28.27] | 3.26 [-21.5, 26.96] | 6.56 [-12.16, 24.97] | -3.29 [-30.17, 22.65] | -1.55 [-34.9, 31.81] | -21.61 [-55.45, 11.97] | -7.13 [-37.4, 22.95] | 1.96 [-20.08, 24.03] | **-59.31 [-91.57, -27.33]** | 4.26 [-26, 35.67] | -12.94 [-36.56, 9.79] | 1.47 [-32, 35.43] | VIB | **-33.52 [-66.89, -0.18]** |
| 35.14 [-0.21, 68.64] | **36.69 [4.36, 68.4]** | **40.03 [12.44, 67.74]** | 30.13 [-3.74, 63.46] | **31.96 [4.47, 59.42]** | 11.9 [-15.76, 39.6] | 26.3 [-9.97, 63.31] | **35.38 [5.53, 65.9]** | -25.79 [-64.34, 12.49] | **37.85 [1.17, 75.1]** | 20.56 [-10.84, 51.46] | 35.01 [-4.55, 74.57] | **33.52 [0.18, 66.89]** | WBC |
|  | | | | | | | | | | | | | |
|  | 95% CrI excludes 0; favors row | | |  | Trend favors row treatment | | |  | Trend favors column treatment | | |  | 95% CrI excludes 0; favors column |

Note. Data are presented as mean differences (MDs) with 95% credible intervals (95% CrIs). For DOMS at 48 h after exercise, an MD greater than 0 favors the row-defining intervention, whereas an MD less than 0 favors the column-defining intervention. Network meta-analysis estimates are presented in the lower-left half of the table, and pairwise meta-analysis estimates, where available, are presented in the upper-right half. Values in bold indicate estimates for which the 95% CrI excludes 0.

Supplementary Table 8. League table for CK at 24 h.

| COMP | 22 [-150.41, 197.51] | -11.54 [-239.04, 219.79] | -46.99 [-270.17, 173.79] | -78.3 [-364.48, 209.32] | -42.45 [-312.47, 226.94] | 38.82 [-241.8, 320.17] | -97.24 [-399.72, 205.55] | -227.5 [-533.93, 74.71] | -73.88 [-342.28, 196.87] | -177.7 [-397.09, 32.59] | -11.84 [-223.15, 200.43] | 28.5 [-272.96, 328.56] | -87.38 [-306.21, 134.66] | -174.27 [-460.45, 111.56] |
| --- | --- | --- | --- | --- | --- | --- | --- | --- | --- | --- | --- | --- | --- | --- |
| -22 [-197.51, 150.41] | CON/PBO | -33.66 [-181.53, 116.66] | -69.16 [-209.3, 69.09] | -100.84 [-326.74, 125.49] | -64.14 [-272.28, 141.85] | 16.47 [-204.21, 236.75] | -119.76 [-366.03, 129.34] | **-249.7 [-502.23, -1.08]** | -95.54 [-302.75, 111.19] | **-199.48 [-329.27, -80.63]** | -33.86 [-156.92, 87.67] | 5.88 [-238.52, 252.05] | -109.17 [-246.21, 27.22] | -196.56 [-423.81, 30.34] |
| 11.54 [-219.79, 239.04] | 33.66 [-116.66, 181.53] | CRY | -35.33 [-241.06, 165.45] | -66.85 [-338.78, 202.37] | -30.97 [-291.4, 224.11] | 50.56 [-217.64, 316.91] | -86.24 [-375.39, 204.92] | -216.16 [-510.15, 73.45] | -61.98 [-318.44, 190.78] | -165.43 [-347.38, 2.11] | -0.03 [-195.54, 189.75] | 39.13 [-247.52, 325.22] | -75.61 [-279.72, 123.69] | -162.42 [-437.55, 106.91] |
| 46.99 [-173.79, 270.17] | 69.16 [-69.09, 209.3] | 35.33 [-165.45, 241.06] | CWI | -31.3 [-256.36, 194.13] | 4.29 [-215.72, 226.09] | 85.6 [-171.16, 342.22] | -50.75 [-332.53, 238.29] | -181.08 [-463.77, 105.81] | -26.71 [-248.64, 195.89] | -129.98 [-320.6, 49.19] | 35.4 [-132.78, 205.16] | 75.31 [-205.68, 357.92] | -39.93 [-215.99, 137.61] | -127.54 [-353.5, 100.33] |
| 78.3 [-209.32, 364.48] | 100.84 [-125.49, 326.74] | 66.85 [-202.37, 338.78] | 31.3 [-194.13, 256.36] | CWT | 36.26 [-260.85, 333.11] | 117.29 [-196.64, 430.01] | -19.23 [-354.14, 317.85] | -150.08 [-485.73, 188.31] | 5.16 [-290.01, 298.62] | -98.48 [-361.25, 153.26] | 66.48 [-184.98, 318.21] | 107.57 [-226.3, 438.28] | -8.29 [-266.44, 248.95] | -95.9 [-344, 151.08] |
| 42.45 [-226.94, 312.47] | 64.14 [-141.85, 272.28] | 30.97 [-224.11, 291.4] | -4.29 [-226.09, 215.72] | -36.26 [-333.11, 260.85] | ES | 80.79 [-212.29, 373.69] | -55.46 [-377.76, 270.14] | -185.44 [-509.8, 140.49] | -31.16 [-279.02, 216.74] | -134.68 [-381.9, 100.9] | 30.29 [-186.74, 249.33] | 70.37 [-249.91, 394.19] | -44.42 [-265.15, 179.23] | -131.89 [-426.42, 166.68] |
| -38.82 [-320.17, 241.8] | -16.47 [-236.75, 204.21] | -50.56 [-316.91, 217.64] | -85.6 [-342.22, 171.16] | -117.29 [-430.01, 196.64] | -80.79 [-373.69, 212.29] | KT | -135.27 [-470.44, 195.57] | -266.91 [-597.08, 67.84] | -112.18 [-405.12, 182.56] | -215.54 [-473.05, 32.21] | -50.84 [-270.51, 170.81] | -9.9 [-338.27, 318.4] | -125.85 [-381.91, 128.64] | -212.92 [-527.02, 102.7] |
| 97.24 [-205.55, 399.72] | 119.76 [-129.34, 366.03] | 86.24 [-204.92, 375.39] | 50.75 [-238.29, 332.53] | 19.23 [-317.85, 354.14] | 55.46 [-270.14, 377.76] | 135.27 [-195.57, 470.44] | LIFU | -131.24 [-485.26, 224.05] | 24.68 [-303.02, 346.17] | -79.2 [-365.56, 190.31] | 85.69 [-194.26, 362.13] | 126.03 [-225.66, 473.19] | 10.42 [-275.4, 294.3] | -77.32 [-415.59, 257.81] |
| 227.5 [-74.71, 533.93] | **249.7 [1.08, 502.23]** | 216.16 [-73.45, 510.15] | 181.08 [-105.81, 463.77] | 150.08 [-188.31, 485.73] | 185.44 [-140.49, 509.8] | 266.91 [-67.84, 597.08] | 131.24 [-224.05, 485.26] | LLLT | 154.39 [-170.12, 478.91] | 50.63 [-233.76, 327.41] | 215.82 [-60.52, 494.57] | 256.19 [-93.51, 606.53] | 140.82 [-144.23, 427.61] | 53.8 [-283.47, 389.62] |
| 73.88 [-196.87, 342.28] | 95.54 [-111.19, 302.75] | 61.98 [-190.78, 318.44] | 26.71 [-195.89, 248.64] | -5.16 [-298.62, 290.01] | 31.16 [-216.74, 279.02] | 112.18 [-182.56, 405.12] | -24.68 [-346.17, 303.02] | -154.39 [-478.91, 170.12] | Massage | -103.81 [-350.25, 133] | 61.86 [-155.27, 278.27] | 101.26 [-215.38, 422.97] | -13.09 [-235.53, 207.47] | -101.56 [-396.72, 194.37] |
| 177.7 [-32.59, 397.09] | **199.48 [80.63, 329.27]** | 165.43 [-2.11, 347.38] | 129.98 [-49.19, 320.6] | 98.48 [-153.26, 361.25] | 134.68 [-100.9, 381.9] | 215.54 [-32.21, 473.05] | 79.2 [-190.31, 365.56] | -50.63 [-327.41, 233.76] | 103.81 [-133, 350.25] | PHO | 165.18 [-3.54, 345.7] | 204.39 [-60.37, 486.11] | 89.65 [-89.95, 280.56] | 2.49 [-251.69, 266.89] |
| 11.84 [-200.43, 223.15] | 33.86 [-87.67, 156.92] | 0.03 [-189.75, 195.54] | -35.4 [-205.16, 132.78] | -66.48 [-318.21, 184.98] | -30.29 [-249.33, 186.74] | 50.84 [-170.81, 270.51] | -85.69 [-362.13, 194.26] | -215.82 [-494.57, 60.52] | -61.86 [-278.27, 155.27] | -165.18 [-345.7, 3.54] | STR | 39.73 [-231.86, 315.92] | -74.98 [-244.79, 92.2] | -162.71 [-413.82, 91.56] |
| -28.5 [-328.56, 272.96] | -5.88 [-252.05, 238.52] | -39.13 [-325.22, 247.52] | -75.31 [-357.92, 205.68] | -107.57 [-438.28, 226.3] | -70.37 [-394.19, 249.91] | 9.9 [-318.4, 338.27] | -126.03 [-473.19, 225.66] | -256.19 [-606.53, 93.51] | -101.26 [-422.97, 215.38] | -204.39 [-486.11, 60.37] | -39.73 [-315.92, 231.86] | SW | -115.34 [-397.9, 166.64] | -202.63 [-537.43, 130.68] |
| 87.38 [-134.66, 306.21] | 109.17 [-27.22, 246.21] | 75.61 [-123.69, 279.72] | 39.93 [-137.61, 215.99] | 8.29 [-248.95, 266.44] | 44.42 [-179.23, 265.15] | 125.85 [-128.64, 381.91] | -10.42 [-294.3, 275.4] | -140.82 [-427.61, 144.23] | 13.09 [-207.47, 235.53] | -89.65 [-280.56, 89.95] | 74.98 [-92.2, 244.79] | 115.34 [-166.64, 397.9] | VIB | -86.58 [-347.59, 171.19] |
| 174.27 [-111.56, 460.45] | 196.56 [-30.34, 423.81] | 162.42 [-106.91, 437.55] | 127.54 [-100.33, 353.5] | 95.9 [-151.08, 344] | 131.89 [-166.68, 426.42] | 212.92 [-102.7, 527.02] | 77.32 [-257.81, 415.59] | -53.8 [-389.62, 283.47] | 101.56 [-194.37, 396.72] | -2.49 [-266.89, 251.69] | 162.71 [-91.56, 413.82] | 202.63 [-130.68, 537.43] | 86.58 [-171.19, 347.59] | WBC |
|  | | | | | | | | | | | | | | |
|  | 95% CrI excludes 0; favors row | |  | Trend favors row treatment | | |  | Trend favors column treatment | | |  | 95% CrI excludes 0; favors column | | |

Note. Data are presented as mean differences (MDs) with 95% credible intervals (95% CrIs). For CK at 24 h after exercise, an MD greater than 0 favors the row-defining intervention, whereas an MD less than 0 favors the column-defining intervention. Network meta-analysis estimates are presented in the lower-left half of the table, and pairwise meta-analysis estimates, where available, are presented in the upper-right half. Values in bold indicate estimates for which the 95% CrI excludes 0.

Supplementary Table 9. League table for CK at 48 h.

| COMP | 52.06 [-274.21, 385.61] | -1.48 [-388.6, 387.59] | 27.49 [-359.02, 420.01] | 3.6 [-463.52, 477.85] | 12.59 [-439.99, 468.15] | 69.29 [-342.39, 485.83] | **-448.94 [-916.94, -8.75]** | 4.05 [-451.52, 460.46] | -200.02 [-567.77, 171.62] | 16.11 [-373.1, 409.02] | -8.54 [-497.69, 489.83] | 35.32 [-350.82, 423.81] | -116.54 [-584.76, 357.24] |
| --- | --- | --- | --- | --- | --- | --- | --- | --- | --- | --- | --- | --- | --- |
| -52.06 [-385.61, 274.21] | CON/PBO | -53.36 [-259.44, 147.75] | -24.14 [-229.12, 178.32] | -48.67 [-386.23, 291.26] | -39.58 [-350.4, 267.84] | 16.56 [-229.97, 267.29] | **-499.96 [-831.59, -203.59]** | -47.11 [-362.06, 260.72] | **-252.5 [-420.45, -87.47]** | -35.69 [-245.28, 171.88] | -60.15 [-429.57, 311.05] | -16.48 [-220.79, 186.8] | -168.96 [-506.92, 172.02] |
| 1.48 [-387.59, 388.6] | 53.36 [-147.75, 259.44] | CRY | 29.12 [-257.32, 317.53] | 5.01 [-385.93, 402.7] | 13.58 [-352.38, 382.13] | 70.11 [-248.74, 395.56] | **-445.81 [-835.5, -88.43]** | 5.63 [-368.07, 374.76] | -198.43 [-417.69, 20.8] | 17.83 [-273.83, 310.53] | -7.42 [-423.75, 422.43] | 36.37 [-246.99, 325.96] | -115.55 [-506.6, 281.65] |
| -27.49 [-420.01, 359.02] | 24.14 [-178.32, 229.12] | -29.12 [-317.53, 257.32] | CWI | -24.47 [-358.2, 315.05] | -14.9 [-348.17, 311.39] | 40.83 [-274.74, 358.62] | **-476.09 [-868.44, -120.43]** | -23.55 [-357.84, 305.66] | -227.61 [-494.32, 36.08] | -10.96 [-279.12, 250.68] | -35.6 [-458.63, 387.59] | 7.63 [-255.91, 269.67] | -145.15 [-482.23, 195.77] |
| -3.6 [-477.85, 463.52] | 48.67 [-291.26, 386.23] | -5.01 [-402.7, 385.93] | 24.47 [-315.05, 358.2] | CWT | 8.33 [-438.36, 449.67] | 65.87 [-353.35, 482.84] | **-451.41 [-934.45, -11.3]** | 0.97 [-446.5, 438.55] | -203.6 [-583.26, 170.16] | 13.19 [-378.28, 396.42] | -11.57 [-512.27, 487.98] | 31.24 [-354.72, 416.67] | -120.69 [-493.19, 252.51] |
| -12.59 [-468.15, 439.99] | 39.58 [-267.84, 350.4] | -13.58 [-382.13, 352.38] | 14.9 [-311.39, 348.17] | -8.33 [-449.67, 438.36] | ES | 55.98 [-327.38, 445.68] | **-459.67 [-915.76, -36.12]** | -8.16 [-381.02, 362.07] | -212.94 [-563.61, 138.09] | 3.37 [-327.03, 339.42] | -20.7 [-499.81, 467.55] | 22.37 [-306.15, 356.34] | -130.01 [-573, 318.73] |
| -69.29 [-485.83, 342.39] | -16.56 [-267.29, 229.97] | -70.11 [-395.56, 248.74] | -40.83 [-358.62, 274.74] | -65.87 [-482.84, 353.35] | -55.98 [-445.68, 327.38] | KT | **-517.56 [-937.78, -133.68]** | -64.93 [-454.84, 323.21] | -269.24 [-571.56, 26.22] | -52.62 [-339.28, 229.3] | -76.77 [-522.58, 369.35] | -33.28 [-350.27, 279.9] | -185.42 [-603.94, 236.43] |
| **448.94 [8.75, 916.94]** | **499.96 [203.59, 831.59]** | **445.81 [88.43, 835.5]** | **476.09 [120.43, 868.44]** | **451.41 [11.3, 934.45]** | **459.67 [36.12, 915.76]** | **517.56 [133.68, 937.78]** | LLLT | **452.04 [29.61, 911.98]** | 247.3 [-91.71, 617.19] | **463.99 [103.61, 860.41]** | 439.16 [-26.84, 942.17] | **482.5 [128.81, 873.32]** | 331.05 [-111.49, 810.82] |
| -4.05 [-460.46, 451.52] | 47.11 [-260.72, 362.06] | -5.63 [-374.76, 368.07] | 23.55 [-305.66, 357.84] | -0.97 [-438.55, 446.5] | 8.16 [-362.07, 381.02] | 64.93 [-323.21, 454.84] | **-452.04 [-911.98, -29.61]** | Massage | -204.19 [-557.02, 148.86] | 12.34 [-317.86, 344.61] | -12 [-493.26, 472.67] | 30.74 [-302.52, 364.79] | -121.18 [-562.58, 324.73] |
| 200.02 [-171.62, 567.77] | **252.5 [87.47, 420.45]** | 198.43 [-20.8, 417.69] | 227.61 [-36.08, 494.32] | 203.6 [-170.16, 583.26] | 212.94 [-138.09, 563.61] | 269.24 [-26.22, 571.56] | -247.3 [-617.19, 91.71] | 204.19 [-148.86, 557.02] | PHO | 216.82 [-49.99, 485.66] | 192.31 [-211.35, 604.81] | 235.14 [-25.6, 500.91] | 83.95 [-290.21, 467.62] |
| -16.11 [-409.02, 373.1] | 35.69 [-171.88, 245.28] | -17.83 [-310.53, 273.83] | 10.96 [-250.68, 279.12] | -13.19 [-396.42, 378.28] | -3.37 [-339.42, 327.03] | 52.62 [-229.3, 339.28] | **-463.99 [-860.41, -103.61]** | -12.34 [-344.61, 317.86] | -216.82 [-485.66, 49.99] | STR | -24.64 [-444.1, 401.68] | 19.03 [-244.29, 285.5] | -133.07 [-515.47, 257.8] |
| 8.54 [-489.83, 497.69] | 60.15 [-311.05, 429.57] | 7.42 [-422.43, 423.75] | 35.6 [-387.59, 458.63] | 11.57 [-487.98, 512.27] | 20.7 [-467.55, 499.81] | 76.77 [-369.35, 522.58] | -439.16 [-942.17, 26.84] | 12 [-472.67, 493.26] | -192.31 [-604.81, 211.35] | 24.64 [-401.68, 444.1] | SW | 43.29 [-377.4, 464.69] | -109.24 [-610.49, 392.88] |
| -35.32 [-423.81, 350.82] | 16.48 [-186.8, 220.79] | -36.37 [-325.96, 246.99] | -7.63 [-269.67, 255.91] | -31.24 [-416.67, 354.72] | -22.37 [-356.34, 306.15] | 33.28 [-279.9, 350.27] | **-482.5 [-873.32, -128.81]** | -30.74 [-364.79, 302.52] | -235.14 [-500.91, 25.6] | -19.03 [-285.5, 244.29] | -43.29 [-464.69, 377.4] | VIB | -152 [-537.99, 234.14] |
| 116.54 [-357.24, 584.76] | 168.96 [-172.02, 506.92] | 115.55 [-281.65, 506.6] | 145.15 [-195.77, 482.23] | 120.69 [-252.51, 493.19] | 130.01 [-318.73, 573] | 185.42 [-236.43, 603.94] | -331.05 [-810.82, 111.49] | 121.18 [-324.73, 562.58] | -83.95 [-467.62, 290.21] | 133.07 [-257.8, 515.47] | 109.24 [-392.88, 610.49] | 152 [-234.14, 537.99] | WBC |
|  | | | | | | | | | | | | | |
|  | 95% CrI excludes 0; favors row | | |  | Trend favors row treatment | | |  | Trend favors column treatment | | |  | 95% CrI excludes 0; favors column |

Note. Data are presented as mean differences (MDs) with 95% credible intervals (95% CrIs). For CK at 48 h after exercise, an MD greater than 0 favors the row-defining intervention, whereas an MD less than 0 favors the column-defining intervention. Network meta-analysis estimates are presented in the lower-left half of the table, and pairwise meta-analysis estimates, where available, are presented in the upper-right half. Values in bold indicate estimates for which the 95% CrI excludes 0.

Supplementary Table 10. League table for IL-6 at 24 h.

| COMP | 0.77 [-61.97, 62.88] | 0.29 [-108.16, 107.64] | -96.56 [-206.53, 4.96] | -90.68 [-198.16, 8.55] | -0.12 [-83.57, 81.52] | -7.28 [-114.45, 101.33] | -64.4 [-181.63, 50.21] | -51.65 [-160.63, 49.93] | -3.08 [-110.51, 105.51] | -41.85 [-150.55, 60.09] | -5.12 [-102.47, 60.37] | 0.96 [-108.29, 109.96] |
| --- | --- | --- | --- | --- | --- | --- | --- | --- | --- | --- | --- | --- |
| -0.77 [-62.88, 61.97] | CON/PBO | -0.51 [-88.93, 88.1] | **-97.26 [-186.18, -15.25]** | **-91.35 [-177.45, -11.83]** | -0.88 [-84.06, 81.09] | -8.04 [-95.61, 79.85] | -64.98 [-164.23, 31.43] | -52.35 [-140.69, 29.2] | -3.81 [-91.47, 84.24] | -42.5 [-131.53, 38.77] | -7.64 [-73.16, 30.07] | 0.19 [-88.8, 88.3] |
| -0.29 [-107.64, 108.16] | 0.51 [-88.1, 88.93] | CRY | -96.51 [-223.61, 23.58] | -90.6 [-216.39, 26.39] | -0.42 [-120.48, 121.85] | -7.49 [-129.81, 118.79] | -64.39 [-197.73, 66.62] | -51.4 [-178.15, 68.8] | -3.32 [-129.16, 124.01] | -41.63 [-167.02, 77.01] | -4.15 [-125.05, 83.25] | 0.67 [-125.57, 126.13] |
| 96.56 [-4.96, 206.53] | **97.26 [15.25, 186.18]** | 96.51 [-23.58, 223.61] | CWI | 5.94 [-86.02, 98.73] | 96.11 [-19.24, 218.09] | 88.84 [-30.13, 215.4] | 32.4 [-95.3, 164.74] | 44.33 [-50.24, 138.87] | 93.06 [-25.7, 220.58] | 54.58 [-39.23, 148.32] | 87.18 [-6.45, 165.01] | 97.03 [-21.42, 224.21] |
| 90.68 [-8.55, 198.16] | **91.35 [11.83, 177.45]** | 90.6 [-26.39, 216.39] | -5.94 [-98.73, 86.02] | ES | 90.35 [-22.41, 211.03] | 82.91 [-34.95, 208.31] | 26.48 [-96.63, 155] | 38.05 [-53.17, 130.34] | 87.35 [-28.6, 211.77] | 48.97 [-42.16, 141.54] | 81.91 [-10.32, 156.39] | 91.4 [-26.05, 216.29] |
| 0.12 [-81.52, 83.57] | 0.88 [-81.09, 84.06] | 0.42 [-121.85, 120.48] | -96.11 [-218.09, 19.24] | -90.35 [-211.03, 22.41] | KT | -7.17 [-126.18, 115.06] | -64.05 [-192.57, 64.32] | -51.08 [-172.16, 63.25] | -2.96 [-121.73, 117.62] | -41.41 [-162.23, 72.65] | -4 [-117.84, 76.8] | 1.1 [-119.36, 122.12] |
| 7.28 [-101.33, 114.45] | 8.04 [-79.85, 95.61] | 7.49 [-118.79, 129.81] | -88.84 [-215.4, 30.13] | -82.91 [-208.31, 34.95] | 7.17 [-115.06, 126.18] | LIFU | -56.6 [-188.57, 72.28] | -43.83 [-171.5, 74.43] | 4.11 [-120.29, 129.52] | -34.27 [-160.26, 83] | 2.88 [-116.6, 88.65] | 8.19 [-117.59, 132.4] |
| 64.4 [-50.21, 181.63] | 64.98 [-31.43, 164.23] | 64.39 [-66.62, 197.73] | -32.4 [-164.74, 95.3] | -26.48 [-155, 96.63] | 64.05 [-64.32, 192.57] | 56.6 [-72.28, 188.57] | LLLT | 11.71 [-119.09, 137.53] | 60.92 [-66.97, 195.02] | 22.75 [-108.53, 148.55] | 55.22 [-64.81, 155.27] | 65.08 [-65.81, 197.88] |
| 51.65 [-49.93, 160.63] | 52.35 [-29.2, 140.69] | 51.4 [-68.8, 178.15] | -44.33 [-138.87, 50.24] | -38.05 [-130.34, 53.17] | 51.08 [-63.25, 172.16] | 43.83 [-74.43, 171.5] | -11.71 [-137.53, 119.09] | Massage | 48.1 [-69.06, 174.81] | 10.77 [-82.81, 103.41] | 42.53 [-49.7, 119.06] | 52.15 [-66.48, 178.07] |
| 3.08 [-105.51, 110.51] | 3.81 [-84.24, 91.47] | 3.32 [-124.01, 129.16] | -93.06 [-220.58, 25.7] | -87.35 [-211.77, 28.6] | 2.96 [-117.62, 121.73] | -4.11 [-129.52, 120.29] | -60.92 [-195.02, 66.97] | -48.1 [-174.81, 69.06] | PHO | -38.5 [-164.68, 79.19] | -0.97 [-119.62, 83.88] | 4.01 [-121.68, 128.35] |
| 41.85 [-60.09, 150.55] | 42.5 [-38.77, 131.53] | 41.63 [-77.01, 167.02] | -54.58 [-148.32, 39.23] | -48.97 [-141.54, 42.16] | 41.41 [-72.65, 162.23] | 34.27 [-83, 160.26] | -22.75 [-148.55, 108.53] | -10.77 [-103.41, 82.81] | 38.5 [-79.19, 164.68] | STR | 33.15 [-60.93, 110.75] | 42.37 [-75.81, 168.88] |
| 5.12 [-60.37, 102.47] | 7.64 [-30.07, 73.16] | 4.15 [-83.25, 125.05] | -87.18 [-165.01, 6.45] | -81.91 [-156.39, 10.32] | 4 [-76.8, 117.84] | -2.88 [-88.65, 116.6] | -55.22 [-155.27, 64.81] | -42.53 [-119.06, 49.7] | 0.97 [-83.88, 119.62] | -33.15 [-110.75, 60.93] | VIB | 4.93 [-80.3, 123.15] |
| -0.96 [-109.96, 108.29] | -0.19 [-88.3, 88.8] | -0.67 [-126.13, 125.57] | -97.03 [-224.21, 21.42] | -91.4 [-216.29, 26.05] | -1.1 [-122.12, 119.36] | -8.19 [-132.4, 117.59] | -65.08 [-197.88, 65.81] | -52.15 [-178.07, 66.48] | -4.01 [-128.35, 121.68] | -42.37 [-168.88, 75.81] | -4.93 [-123.15, 80.3] | WBC |
|  | | | | | | | | | | | | |
|  | 95% CrI excludes 0; favors row | |  | Trend favors row treatment | |  | Trend favors column treatment | | |  | 95% CrI excludes 0; favors column | |

Note. Data are presented as mean differences (MDs) with 95% credible intervals (95% CrIs). For IL-6 at 24 h after exercise, an MD greater than 0 favors the row-defining intervention, whereas an MD less than 0 favors the column-defining intervention. Network meta-analysis estimates are presented in the lower-left half of the table, and pairwise meta-analysis estimates, where available, are presented in the upper-right half. Values in bold indicate estimates for which the 95% CrI excludes 0.

Supplementary Table 11. League table for IL-6 at 48 h.

| COMP | -0.08 [-42.23, 42.34] | -0.29 [-72.02, 72.19] | **-65.69 [-144.41, -3.47]** | -21.66 [-102.74, 39.61] | -0.9 [-55.83, 53.7] | -79.45 [-163.95, 5.42] | -26.13 [-105.37, 33.67] | -2.61 [-74.73, 69.55] | -21.19 [-99.65, 40.3] | -4.14 [-66.39, 42.04] |
| --- | --- | --- | --- | --- | --- | --- | --- | --- | --- | --- |
| 0.08 [-42.34, 42.23] | CON/PBO | -0.24 [-58.56, 57.84] | **-66.19 [-130.87, -17.27]** | -22.22 [-88.27, 26.49] | -0.87 [-55.66, 54.17] | **-79.34 [-153.29, -5.12]** | -26.72 [-91.24, 18.72] | -2.53 [-61.31, 55.09] | -21.48 [-87.28, 26.65] | -5.26 [-46.15, 21.89] |
| 0.29 [-72.19, 72.02] | 0.24 [-57.84, 58.56] | CRY | -64.97 [-153.88, 8.29] | -20.7 [-112.59, 50.04] | -0.61 [-80.93, 79.62] | -79.14 [-172.23, 14.93] | -25.31 [-116.38, 43.57] | -2.35 [-84.25, 79.9] | -20.49 [-111.14, 51.43] | -3.04 [-78.8, 56.07] |
| **65.69 [3.47, 144.41]** | **66.19 [17.27, 130.87]** | 64.97 [-8.29, 153.88] | CWI | 44.06 [-20.59, 106.17] | 64.54 [-5.15, 151.51] | -12.3 [-98.61, 86.76] | 38.81 [-23.19, 98.65] | 62.77 [-9.76, 152.73] | 44.55 [-19.02, 106.98] | **59.36 [5.97, 118.88]** |
| 21.66 [-39.61, 102.74] | 22.22 [-26.49, 88.27] | 20.7 [-50.04, 112.59] | -44.06 [-106.17, 20.59] | ES | 20.36 [-48.53, 109.88] | -55.93 [-142.32, 44.06] | -5.45 [-66.6, 55.73] | 18.48 [-53.86, 110.19] | 0.47 [-62.93, 64.88] | 15.63 [-38.14, 76.22] |
| 0.9 [-53.7, 55.83] | 0.87 [-54.17, 55.66] | 0.61 [-79.62, 80.93] | -64.54 [-151.51, 5.15] | -20.36 [-109.88, 48.53] | KT | -78.58 [-170.67, 14.23] | -24.8 [-113.15, 42.58] | -1.73 [-81.78, 78.41] | -20.02 [-107.55, 50.24] | -2.62 [-75.96, 53.7] |
| 79.45 [-5.42, 163.95] | **79.34 [5.12, 153.29]** | 79.14 [-14.93, 172.23] | 12.3 [-86.76, 98.61] | 55.93 [-44.06, 142.32] | 78.58 [-14.23, 170.67] | LLLT | 51.08 [-48.06, 136.24] | 76.6 [-17.13, 169.92] | 57.09 [-42.21, 144.09] | 72.42 [-12.88, 148.87] |
| 26.13 [-33.67, 105.37] | 26.72 [-18.72, 91.24] | 25.31 [-43.57, 116.38] | -38.81 [-98.65, 23.19] | 5.45 [-55.73, 66.6] | 24.8 [-42.58, 113.15] | -51.08 [-136.24, 48.06] | Massage | 22.94 [-45.99, 113.94] | 5.73 [-54.95, 68.28] | 20.41 [-29.89, 78.67] |
| 2.61 [-69.55, 74.73] | 2.53 [-55.09, 61.31] | 2.35 [-79.9, 84.25] | -62.77 [-152.73, 9.76] | -18.48 [-110.19, 53.86] | 1.73 [-78.41, 81.78] | -76.6 [-169.92, 17.13] | -22.94 [-113.94, 45.99] | PHO | -18.18 [-109.05, 53.82] | -0.85 [-76.47, 58.75] |
| 21.19 [-40.3, 99.65] | 21.48 [-26.65, 87.28] | 20.49 [-51.43, 111.14] | -44.55 [-106.98, 19.02] | -0.47 [-64.88, 62.93] | 20.02 [-50.24, 107.55] | -57.09 [-144.09, 42.21] | -5.73 [-68.28, 54.95] | 18.18 [-53.82, 109.05] | STR | 14.92 [-38.49, 74.47] |
| 4.14 [-42.04, 66.39] | 5.26 [-21.89, 46.15] | 3.04 [-56.07, 78.8] | **-59.36 [-118.88, -5.97]** | -15.63 [-76.22, 38.14] | 2.62 [-53.7, 75.96] | -72.42 [-148.87, 12.88] | -20.41 [-78.67, 29.89] | 0.85 [-58.75, 76.47] | -14.92 [-74.47, 38.49] | VIB |
|  | | | | | | | | | | |
|  | 95% CrI excludes 0; favors row | |  | Trend favors row treatment | |  | Trend favors column treatment | |  | 95% CrI excludes 0; favors column |

Note. Data are presented as mean differences (MDs) with 95% credible intervals (95% CrIs). For IL-6 at 48 h after exercise, an MD greater than 0 favors the row-defining intervention, whereas an MD less than 0 favors the column-defining intervention. Network meta-analysis estimates are presented in the lower-left half of the table, and pairwise meta-analysis estimates, where available, are presented in the upper-right half. Values in bold indicate estimates for which the 95% CrI excludes 0.

5.2 Certainty of evidence: direct, indirect, and network estimates

The certainty ratings in Supplementary Tables 12–17 are network-level CINeMA judgments and should not be interpreted as ratings of direct evidence alone. To distinguish evidence sources explicitly, the GRADE approach for NMA was additionally applied to comparisons for which independent direct and indirect estimates were available. Direct evidence was assessed from head-to-head trials; indirect evidence was assessed from the contributing first-order pathways with consideration of intransitivity; and the final network certainty incorporated source-specific certainty, network-level imprecision, and incoherence^[1, 2]^.

| Supplementary Table 12. Network Summary of Findings (CINeMA) table for DOMS at 24 h. | | | | |
| --- | --- | --- | --- | --- |
| DOMS at 24 h (Total studies: 22; Total participants/arms: 538) | | | | |
| Comparator (reference) | Relative effect** (MD [95% CrI]) | Ranking*** | Certainty of network evidence (CINeMA) | Interpretation of findings |
| WBC (1 RCT, 12 participants) | -38.92 [-69.08, -9.15] Network estimate | 1 | ⊕◯◯◯ Very Low Due to serious risk of bias, reporting bias and incoherence | Estimate favors intervention; evidence remains very uncertain. |
| LIFU (1 RCT, 8 participants) | -34.54 [-62.45, -6.61] Network estimate | 2 | ⊕◯◯◯ Very Low Due to serious risk of bias, reporting bias, heterogeneity and incoherence | Estimate favors intervention; evidence remains very uncertain. |
| CWT (1 RCT, 12 participants) | -26.92 [-56.89, 2.58] Network estimate | 3 | ⊕◯◯◯ Very Low Due to serious risk of bias, reporting bias, heterogeneity and incoherence | Uncertain; 95% CrI includes 0. |
| PHO (4 RCTs, 39 participants) | -18.20 [-32.91, -3.72] Network estimate | 4 | ⊕◯◯◯ Very Low Due to risk of bias, reporting bias, heterogeneity and incoherence | Estimate favors intervention; evidence remains very uncertain. |
| ES (2 RCTs, 21 participants) | -15.55 [-38.87, 7.64] Network estimate | 5 | ⊕◯◯◯ Very Low Due to serious risk of bias, reporting bias, imprecision, heterogeneity and incoherence | Uncertain; 95% CrI includes 0. |
| COMP (3 RCTs, 23 participants) | -10.03 [-26.34, 5.87] Network estimate | 6 | ⊕◯◯◯ Very Low Due to serious risk of bias, reporting bias, imprecision, heterogeneity and incoherence | Uncertain; 95% CrI includes 0. |
| CWI (1 RCT, 12 participants) | -8.92 [-38.92, 20.66] Network estimate | 7 | ⊕◯◯◯ Very Low Due to serious risk of bias, reporting bias, serious imprecision and incoherence | Uncertain; 95% CrI includes 0. |
| CRY (2 RCTs, 22 participants) | -8.59 [-28.40, 10.72] Network estimate | 8 | ⊕◯◯◯ Very Low Due to serious risk of bias, reporting bias, imprecision, heterogeneity and incoherence | Uncertain; 95% CrI includes 0. |
| LLLT (2 RCTs, 25 participants) | -7.21 [-30.97, 17.01] Network estimate | 9 | ⊕◯◯◯ Very Low Due to risk of bias, reporting bias, serious imprecision and incoherence | Uncertain; 95% CrI includes 0. |
| KT (4 RCTs, 58 participants) | -6.20 [-20.06, 7.91] Network estimate | 10 | ⊕◯◯◯ Very Low Due to serious risk of bias, reporting bias, imprecision, heterogeneity and incoherence | Uncertain; 95% CrI includes 0. |
| VIB (2 RCTs, 23 participants) | -4.69 [-24.63, 15.73] Network estimate | 11 | ⊕◯◯◯ Very Low Due to serious risk of bias, reporting bias, serious imprecision and incoherence | Uncertain; 95% CrI includes 0. |
| AC (2 RCTs, 22 participants) | -2.13 [-24.98, 18.56] Network estimate | 12 | ⊕◯◯◯ Very Low Due to risk of bias, reporting bias, serious imprecision and incoherence | Uncertain; 95% CrI includes 0. |
| STR (1 RCT, 14 participants) | -0.03 [-28.40, 28.26] Network estimate | 13 | ⊕◯◯◯ Very Low Due to serious risk of bias, reporting bias, serious imprecision and incoherence | Uncertain; 95% CrI includes 0. |
| Control group | Reference comparator | 14 | No estimate | Reference comparator |

| Supplementary Table 13. Network Summary of Findings (CINeMA) table for DOMS at 48 h. | | | | |
| --- | --- | --- | --- | --- |
| DOMS at 48 h (Total studies: 23; Total participants/arms: 592) | | | | |
| Comparator (reference) | Relative effect** (MD [95% CrI]) | Ranking*** | Certainty of network evidence (CINeMA) | Interpretation of findings |
| LIFU (1 RCT, 8 participants) | -65.81 [-92.30, -39.66] Network estimate | 1 | ⊕◯◯◯ Very Low Due to serious risk of bias, reporting bias and incoherence | Estimate favors intervention; evidence remains very uncertain. |
| WBC (1 RCT, 12 participants) | -40.03 [-67.74, -12.44] Network estimate | 2 | ⊕◯◯◯ Very Low Due to serious risk of bias, reporting bias and incoherence | Estimate favors intervention; evidence remains very uncertain. |
| CWT (1 RCT, 12 participants) | -28.08 [-56.27, -0.26] Network estimate | 3 | ⊕◯◯◯ Very Low Due to serious risk of bias, reporting bias, heterogeneity and incoherence | Estimate favors intervention; evidence remains very uncertain. |
| PHO (4 RCTs, 39 participants) | -19.46 [-33.55, -5.99] Network estimate | 4 | ⊕⊕◯◯ Low Due to risk of bias, reporting bias, heterogeneity and incoherence | Estimate favors intervention; certainty is low. |
| ES (2 RCTs, 21 participants) | -13.64 [-37.64, 10.22] Network estimate | 5 | ⊕◯◯◯ Very Low Due to serious risk of bias, reporting bias, imprecision, heterogeneity and incoherence | Uncertain; 95% CrI includes 0. |
| CRY (2 RCTs, 22 participants) | -9.88 [-28.93, 8.48] Network estimate | 6 | ⊕◯◯◯ Very Low Due to serious risk of bias, reporting bias, imprecision, heterogeneity and incoherence | Uncertain; 95% CrI includes 0. |
| CWI (1 RCT, 12 participants) | -8.06 [-35.87, 19.70] Network estimate | 7 | ⊕◯◯◯ Very Low Due to serious risk of bias, reporting bias, serious imprecision and incoherence | Uncertain; 95% CrI includes 0. |
| VIB (2 RCTs, 23 participants) | -6.56 [-24.97, 12.16] Network estimate | 8 | ⊕◯◯◯ Very Low Due to serious risk of bias, reporting bias, imprecision, heterogeneity and incoherence | Uncertain; 95% CrI includes 0. |
| STR (1 RCT, 14 participants) | -5.10 [-32.95, 23.22] Network estimate | 9 | ⊕◯◯◯ Very Low Due to serious risk of bias, reporting bias, serious imprecision and incoherence | Uncertain; 95% CrI includes 0. |
| AC (2 RCTs, 22 participants) | -4.88 [-26.46, 14.81] Network estimate | 10 | ⊕◯◯◯ Very Low Due to risk of bias, reporting bias, serious imprecision and incoherence | Uncertain; 95% CrI includes 0. |
| KT (5 RCTs, 85 participants) | -4.63 [-16.49, 7.36] Network estimate | 11 | ⊕◯◯◯ Very Low Due to serious risk of bias, reporting bias, imprecision, heterogeneity and incoherence | Uncertain; 95% CrI includes 0. |
| COMP (3 RCTs, 23 participants) | -3.31 [-19.38, 12.08] Network estimate | 12 | ⊕◯◯◯ Very Low Due to serious risk of bias, reporting bias, imprecision, heterogeneity and incoherence | Uncertain; 95% CrI includes 0. |
| LLLT (2 RCTs, 25 participants) | -2.16 [-26.49, 22.86] Network estimate | 13 | ⊕◯◯◯ Very Low Due to risk of bias, reporting bias, serious imprecision and incoherence | Uncertain; 95% CrI includes 0. |
| Control group | Reference comparator | 14 | No estimate | Reference comparator |

| Supplementary Table 14. Network Summary of Findings (CINeMA) table for CK at 24 h. | | | | |
| --- | --- | --- | --- | --- |
| CK at 24 h (Total studies: 21; Total participants/arms: 515) | | | | |
| Comparator (reference) | Relative effect** (MD [95% CrI]) | Ranking*** | Certainty of network evidence (CINeMA) | Interpretation of findings |
| LLLT (1 RCT, 7 participants) | -249.7 [-502.23, -1.08] Network estimate | 1 | ⊕⊕◯◯ Low Due to risk of bias, reporting bias and incoherence | Estimate favors intervention; certainty is low. |
| PHO (4 RCTs, 38 participants) | -199.48 [-329.27, -80.63] Network estimate | 2 | ⊕⊕◯◯ Low Due to risk of bias, reporting bias and incoherence | Estimate favors intervention; certainty is low. |
| WBC (1 RCT, 12 participants) | -196.56 [-423.81, 30.34] Network estimate | 3 | ⊕◯◯◯ Very low Due to serious risk of bias, reporting bias and incoherence | Uncertain; 95% CrI includes 0. |
| VIB (3 RCTs, 28 participants) | -109.17 [-246.21, 27.22] Network estimate | 4 | ⊕◯◯◯ Very low Due to serious risk of bias, reporting bias, heterogeneity and incoherence | Uncertain; 95% CrI includes 0. |
| LIFU (1 RCT, 8 participants) | -119.76 [-366.03, 129.34] Network estimate | 5 | ⊕◯◯◯ Very low Due to serious risk of bias, reporting bias, imprecision and incoherence | Uncertain; 95% CrI includes 0. |
| CWT (1 RCT, 12 participants) | -100.84 [-326.74, 125.49] Network estimate | 6 | ⊕◯◯◯ Very low Due to serious risk of bias, reporting bias, imprecision and incoherence | Uncertain; 95% CrI includes 0. |
| Massage (1 RCT, 5 participants) | -95.54 [-302.75, 111.19] Network estimate | 7 | ⊕⊕◯◯ Low Due to risk of bias, reporting bias, imprecision and incoherence | Uncertain; 95% CrI includes 0. |
| CWI (3 RCTs, 25 participants) | -69.16 [-209.3, 69.09] Network estimate | 8 | ⊕◯◯◯ Very low Due to serious risk of bias, reporting bias, imprecision and incoherence | Uncertain; 95% CrI includes 0. |
| ES (1 RCT, 5 participants) | -64.14 [-272.28, 141.85] Network estimate | 9 | ⊕⊕◯◯ Low Due to risk of bias, reporting bias, imprecision, heterogeneity and incoherence | Uncertain; 95% CrI includes 0. |
| CRY (4 RCTs, 48 participants) | -33.66 [-181.53, 116.66] Network estimate | 10 | ⊕⊕◯◯ Low Due to risk of bias, reporting bias, imprecision, heterogeneity and incoherence | Uncertain; 95% CrI includes 0. |
| STR (4 RCTs, 49 participants) | -33.86 [-156.92, 87.67] Network estimate | 11 | ⊕⊕◯◯ Low Due to risk of bias, reporting bias, imprecision, heterogeneity and incoherence | Uncertain; 95% CrI includes 0. |
| COMP (3 RCTs, 27 participants) | -22 [-197.51, 150.41] Network estimate | 12 | ⊕◯◯◯ Very low Due to risk of bias, reporting bias, serious imprecision and incoherence | Uncertain; 95% CrI includes 0. |
| SW (1 RCT, 16 participants) | 5.88 [-238.52, 252.05] Network estimate | 13 | ⊕◯◯◯ Very low Due to risk of bias, reporting bias, serious imprecision and incoherence | Uncertain; 95% CrI includes 0. |
| KT (1 RCT, 17 participants) | 16.47 [-204.21, 236.75] Network estimate | 14 | ⊕⊕◯◯ Low Due to risk of bias, reporting bias, imprecision, heterogeneity and incoherence | Uncertain; 95% CrI includes 0. |
| CON/PBO (21 studies, 218 participants) | Reference comparator | 15 | No estimate | Reference comparator |

| Supplementary Table 15. Network Summary of Findings (CINeMA) table for CK at 48 h. | | | | |
| --- | --- | --- | --- | --- |
| CK at 48 h (Total studies: 21 Total participants: 575) | | | | |
| Comparator (reference) | Relative effect** (MD [95% CrI]) | Ranking*** | Certainty of network evidence (CINeMA) | Interpretation of findings |
| LLLT (2 RCTs, 25 participants) | -499.96  [-831.59, -203.59] Network estimate | 1 | ⊕⊕◯◯ Low Due to risk of bias, reporting bias and incoherence | Estimate favors intervention; certainty is low. |
| PHO (5 RCTs, 50 participants) | -252.5  [-420.45, -87.47] Network estimate | 2 | ⊕⊕◯◯ Low Due to risk of bias, reporting bias and incoherence | Estimate favors intervention; certainty is low. |
| WBC (1 RCT, 12 participants) | -168.96  [-506.92, 172.02] Network estimate | 3 | ⊕◯◯◯ Very low Due to serious risk of bias, reporting bias and incoherence | Uncertain; 95% CrI includes 0. |
| SW (1 RCT, 16 participants) | -60.15  [-429.57, 311.05] Network estimate | 4 | ⊕⊕◯◯ Low Due to risk of bias, reporting bias, imprecision and incoherence | Uncertain; 95% CrI includes 0. |
| CRY (5 RCTs, 58 participants) | -53.36  [-259.44, 147.75] Network estimate | 5 | ⊕⊕◯◯ Low Due to risk of bias, reporting bias, heterogeneity and incoherence | Uncertain; 95% CrI includes 0. |
| COMP (2 RCTs, 16 participants) | -52.06  [-385.61, 274.21] Network estimate | 6 | ⊕◯◯◯ Very low Due to risk of bias, reporting bias, serious imprecision and incoherence | Uncertain; 95% CrI includes 0. |
| Massage (1 RCT, 5 participants) | -47.11  [-362.06, 260.72] Network estimate | 7 | ⊕⊕◯◯ Low Due to risk of bias, reporting bias, imprecision and incoherence | Uncertain; 95% CrI includes 0. |
| CWT (1 RCT, 12 participants) | -48.67  [-386.23, 291.26] Network estimate | 8 | ⊕◯◯◯ Very low Due to serious risk of bias, reporting bias, imprecision and incoherence | Uncertain; 95% CrI includes 0. |
| ES (1 RCT, 5 participants) | -39.58  [-350.4, 267.84] Network estimate | 9 | ⊕⊕◯◯ Low Due to risk of bias, reporting bias, imprecision, heterogeneity and incoherence | Uncertain; 95% CrI includes 0. |
| STR (3 RCTs, 36 participants) | -35.69  [-245.28, 171.88] Network estimate | 10 | ⊕⊕◯◯ Low Due to risk of bias, reporting bias, heterogeneity and incoherence | Uncertain; 95% CrI includes 0. |
| CWI (3 RCTs, 25 participants) | -24.14  [-229.12, 178.32] Network estimate | 11 | ⊕◯◯◯ Very low Due to serious risk of bias, reporting bias, heterogeneity and incoherence | Uncertain; 95% CrI includes 0. |
| VIB (3 RCTs, 28 participants) | -16.48  [-220.79, 186.8] Network estimate | 12 | ⊕◯◯◯ Very low Due to serious risk of bias, reporting bias, serious heterogeneity and incoherence | Uncertain; 95% CrI includes 0. |
| KT (2 RCTs, 44 participants) | 16.56  [-229.97, 267.29] Network estimate | 14 | ⊕◯◯◯ Very low Due to risk of bias, reporting bias, serious heterogeneity and incoherence | Uncertain; 95% CrI includes 0. |
| Control group (CON/PBO) | Reference comparator | 13 | No estimate | Reference comparator |

Supplementary Figure 1. Forest plots of IL-6 at 24 h and 48 h.

| Supplementary Table 16. Network Summary of Findings (CINeMA) table for IL-6 at 24 h. | | | | |
| --- | --- | --- | --- | --- |
|   Supplementary Figure 2. Effects of different recovery and regeneration modalities on IL-6 at 24 h. (a) Network plot showing direct and indirect comparisons in the network meta-analysis. Node size represents the number of participants assigned to each intervention. Lines between nodes indicate direct comparisons between interventions, and line thickness represents the amount of direct evidence. (b) Bayesian ranking panel. The cumulative probability indicates the probability that an intervention occupies a given rank. Relative rankings were summarized using the surface under the cumulative ranking curve (SUCRA). Higher SUCRA values, corresponding to curves closer to the upper-left corner, indicate a higher relative ranking. | | | | |
| Comparator (reference) | Relative effect** (MD [95% CrI]) | Ranking*** | Certainty of network evidence (CINeMA) | Interpretation of findings |
| CWI (1 RCT, 5 participants) | -97.26 [-186.18, -15.25] Network estimate | 1 | ⊕◯◯◯ Very Low Due to risk of bias, reporting bias and serious incoherence | Estimate favors intervention; evidence remains very uncertain. |
| ES (1 RCT, 5 participants) | -91.35 [-177.45, -11.83] Network estimate | 2 | ⊕◯◯◯ Very Low Due to risk of bias, reporting bias and serious incoherence | Estimate favors intervention; evidence remains very uncertain. |
| LLLT (1 RCT, 7 participants) | -64.98 [-164.23, 31.43] Network estimate | 3 | ⊕◯◯◯ Very Low Due to risk of bias, reporting bias, serious heterogeneity and incoherence | Uncertain; 95% CrI includes 0. |
| Massage (1 RCT, 5 participants) | -52.35 [-140.69, 29.20] Network estimate | 4 | ⊕◯◯◯ Very Low Due to risk of bias, reporting bias, serious heterogeneity and serious incoherence | Uncertain; 95% CrI includes 0. |
| STR (1 RCT, 5 participants) | -42.50 [-131.53, 38.77] Network estimate | 5 | ⊕◯◯◯ Very Low Due to risk of bias, reporting bias, serious heterogeneity and serious incoherence | Uncertain; 95% CrI includes 0. |
| LIFU (1 RCT, 8 participants) | -8.04 [-95.61, 79.85] Network estimate | 6 | ⊕◯◯◯ Very Low Due to serious risk of bias, reporting bias and incoherence | Uncertain; 95% CrI includes 0. |
| VIB (3 RCTs, 28 participants) | -7.64 [-73.16, 30.07] Network estimate | 7 | ⊕◯◯◯ Very Low Due to serious risk of bias, reporting bias, imprecision, heterogeneity and incoherence | Uncertain; 95% CrI includes 0. |
| PHO (1 RCT, 12 participants) | -3.81 [-91.47, 84.24] Network estimate | 8 | ⊕◯◯◯ Very Low Due to risk of bias, reporting bias, serious heterogeneity and incoherence | Uncertain; 95% CrI includes 0. |
| KT (1 RCT, 8 participants) | -0.88 [-84.06, 81.09] Network estimate | 9 | ⊕◯◯◯ Very Low Due to serious risk of bias, reporting bias, imprecision and heterogeneity | Uncertain; 95% CrI includes 0. |
| COMP (2 RCTs, 16 participants) | -0.77 [-62.88, 61.97] Network estimate | 10 | ⊕◯◯◯ Very Low Due to serious risk of bias, reporting bias, imprecision, heterogeneity and incoherence | Uncertain; 95% CrI includes 0. |
| CRY (1 RCT, 12 participants) | -0.51 [-88.93, 88.10] Network estimate | 11 | ⊕◯◯◯ Very Low Due to serious risk of bias, reporting bias, serious imprecision and incoherence | Uncertain; 95% CrI includes 0. |
| WBC (1 RCT, 11 participants) | 0.19 [-88.80, 88.30] Network estimate | 12 | ⊕◯◯◯ Very Low Due to risk of bias, reporting bias, serious imprecision and incoherence | Uncertain; 95% CrI includes 0. |
| Control group | Reference comparator | 13 | No estimate | Reference comparator |

| Supplementary Table 17. Network Summary of Findings (CINeMA) table for IL-6 at 48 h. | | | | |
| --- | --- | --- | --- | --- |
|   Supplementary Figure 3. Effects of different recovery and regeneration modalities on IL-6 at 48 h. (a) Network plot showing direct and indirect comparisons in the network meta-analysis. Node size represents the number of participants assigned to each intervention. Lines between nodes indicate direct comparisons between interventions, and line thickness represents the amount of direct evidence. (b) Bayesian ranking panel. The cumulative probability indicates the probability that an intervention occupies a given rank. Relative rankings were summarized using the surface under the cumulative ranking curve (SUCRA). Higher SUCRA values, corresponding to curves closer to the upper-left corner, indicate a higher relative ranking. | | | | |
| Comparator (reference) | Relative effect** (MD [95% CrI]) | Ranking*** | Certainty of network evidence (CINeMA) | Interpretation of findings |
| LLLT (1 RCT, 7 participants) | -79.34 [-153.29, -5.12] Network estimate | 1 | ⊕◯◯◯ Very Low Due to risk of bias, reporting bias, serious heterogeneity and incoherence | Estimate favors intervention; evidence remains very uncertain. |
| CWI (1 RCT, 5 participants) | -66.19 [-130.87, -17.27] Network estimate | 2 | ⊕◯◯◯ Very Low Due to risk of bias, reporting bias and serious incoherence | Estimate favors intervention; evidence remains very uncertain. |
| Massage (1 RCT, 5 participants) | -26.72 [-91.24, 18.72] Network estimate | 3 | ⊕◯◯◯ Very Low Due to risk of bias, reporting bias, serious heterogeneity and serious incoherence | Uncertain; 95% CrI includes 0. |
| ES (1 RCT, 5 participants) | -22.22 [-88.27, 26.49] Network estimate | 4 | ⊕◯◯◯ Very Low Due to risk of bias, reporting bias, serious imprecision and serious incoherence | Uncertain; 95% CrI includes 0. |
| STR (1 RCT, 5 participants) | -21.48 [-87.28, 26.65] Network estimate | 5 | ⊕◯◯◯ Very Low Due to risk of bias, reporting bias, serious imprecision and serious incoherence | Uncertain; 95% CrI includes 0. |
| VIB (3 RCTs, 28 participants) | -5.26 [-46.15, 21.89] Network estimate | 6 | ⊕◯◯◯ Very Low Due to serious risk of bias, reporting bias, serious heterogeneity and incoherence | Uncertain; 95% CrI includes 0. |
| PHO (1 RCT, 12 participants) | -2.53 [-61.31, 55.09] Network estimate | 7 | ⊕⊕◯◯ Low Due to risk of bias, reporting bias, imprecision, heterogeneity and incoherence | Uncertain; 95% CrI includes 0. |
| KT (1 RCT, 8 participants) | -0.87 [-55.66, 54.17] Network estimate | 8 | ⊕◯◯◯ Very Low Due to serious risk of bias, reporting bias, imprecision and heterogeneity | Uncertain; 95% CrI includes 0. |
| CRY (1 RCT, 12 participants) | -0.24 [-58.56, 57.84] Network estimate | 9 | ⊕◯◯◯ Very Low Due to serious risk of bias, reporting bias, serious imprecision and incoherence | Uncertain; 95% CrI includes 0. |
| COMP (2 RCTs, 16 participants) | 0.08 [-42.34, 42.23] Network estimate | 10 | ⊕◯◯◯ Very Low Due to serious risk of bias, reporting bias, serious imprecision and incoherence | Uncertain; 95% CrI includes 0. |
| Control group | Reference comparator | 11 | No estimate | Reference comparator |

Supplementary Table 17A. GRADE assessment of direct, indirect, and network evidence for mixed-evidence contrasts.

| **Outcome/time** | **Comparison (direct studies)** | **Direct MD (95% CrI)** | **Direct certainty** | **Indirect MD (95% CrI)** | **Indirect certainty** | **Network MD (95% CrI)** | **Network certainty** |
| --- | --- | --- | --- | --- | --- | --- | --- |
| DOMS 24 h | KT vs COMP (1) | −5.07 (−33.4 to 23.5) | Low | 10.0 (−16.0 to 37.6) | Low | 3.85 (−15.1 to 23.4) | Very low |
| DOMS 24 h | CRY vs PHO (1) | 3.94 (−23.5 to 31.5) | Low | 12.4 (−23.5 to 47.9) | Low | 9.60 (−11.9 to 30.8) | Very low |
| DOMS 48 h | KT vs COMP (1) | −3.65 (−29.4 to 21.5) | Low | −3.54 (−26.1 to 22.3) | Low | −1.34 (−18.9 to 17.1) | Very low |
| DOMS 48 h | CRY vs PHO (1) | 5.45 (−20.2 to 31.1) | Low | 8.15 (−26.0 to 42.4) | Low | 9.66 (−10.8 to 29.8) | Very low |
| CK 24 h | STR vs CWI (1) | −4.96 (−284 to 272) | Moderate | 69.2 (−193 to 333) | Low | 35.1 (−135 to 207) | Very low |
| CK 24 h | VIB vs CWI (1) | −31.1 (−305 to 244) | Moderate | −48.9 (−329 to 236) | Low | −40.1 (−217 to 140) | Very low |
| CK 24 h | VIB vs STR (1) | −26.3 (−299 to 245) | Moderate | −121 (−377 to 137) | Low | −75.5 (−245 to 95.3) | Very low |
| CK 24 h | CRY vs PHO (2) | 102 (−176 to 381) | Low | 238 (−17.5 to 518) | Low | 165 (−3.53 to 350) | Very low |
| CK 48 h | VIB vs CWI (1) | −6.15 (−422 to 410) | Moderate | 18.9 (−397 to 434) | Low | 7.46 (−256 to 270) | Very low |
| CK 48 h | STR vs CWI (1) | −4.12 (−419 to 416) | Moderate | −20.5 (−451 to 405) | Low | −10.7 (−275 to 253) | Very low |
| CK 48 h | CRY vs PHO (2) | 130 (−156 to 419) | Low | 310 (−172 to 755) | Low | 199 (−22.0 to 417) | Very low |
| CK 48 h | STR vs VIB (1) | 0.848 (−415 to 417) | Moderate | −39.7 (−471 to 383) | Low | −17.6 (−286 to 246) | Very low |
| CK 48 h | KT vs STR (1) | 28.9 (−381 to 444) | Moderate | 88.8 (−413 to 595) | Low | 53.6 (−229 to 340) | Very low |

Note. Numbers in parentheses after each comparison indicate the number of studies providing direct head-to-head evidence. Source-specific certainty was rated to inform the network estimate. In line with updated GRADE guidance, imprecision was judged primarily at the final network-estimate level to avoid double-counting. Direct evidence was downgraded for risk of bias where appropriate; indirect certainty was based on the lowest-certainty direct evidence in the contributing first-order pathway and consideration of intransitivity. The final network certainty was very low for all 13 mixed-evidence contrasts because the network estimates remained markedly imprecise. Node-splitting did not identify statistically detectable incoherence, so no additional downgrade for incoherence was applied. For IL-6 at 24 h and 48 h, independent direct and indirect evidence could not be separated for the same contrast; source-specific certainty was therefore not estimable, and network-level CINeMA confidence is reported in Supplementary Tables 16–17. CINeMA confidence ratings in Supplementary Tables 12–17 and GRADE certainty ratings in Supplementary Table 17A were generated using distinct assessment frameworks and should not be interpreted as interchangeable categories.

# **6. Sensitivity analyses**

6.1 Potential studies with extreme effect sizes

Supplementary Table 18. Potential studies with extreme effect sizes included in leave-one-out sensitivity analyses.

| Outcome | Time point | Candidate study | Intervention node | Rationale |
| --- | --- | --- | --- | --- |
| DOMS | 24 h | Xia 2022 | LIFU | Effect size markedly larger than those of other studies in the same network |
| DOMS | 48 h | Xia 2022 | LIFU | Very large reduction in DOMS, with the node supported mainly by a single study |
| DOMS | 24 h/48 h | Antonialli 2014 | PHO | Large effects observed for both DOMS and CK |
| DOMS | 24 h/48 h | Xue 2023 | KT/COMP | Large mean difference relative to other studies in the same network. |
| CK | 24 h/48 h | Antonialli 2014 | PHO | CK reduction markedly larger than in other studies |
| CK | 24 h/48 h | Vanin 2016 | LLLT | Large CK effect size, with potential influence on the ranking of LLLT |
| CK | 24 h/48 h | Qu 2020 | WBC/CWT | Influenced multiple cryotherapy-related nodes |
| CK | 24 h/48 h | Machado 2020 | PHO | Large effect size |
| CK | 24 h/48 h | de Paiva 2016 | PHO | A small SD may have inflated the standardized effect size |

6.2 Leave-one-out sensitivity analyses excluding potential extreme-effect studies

Supplementary Table 19. Leave-one-out sensitivity analyses excluding potential extreme-effect studies.

| Outcome | Time point | Excluded study | Main affected node | Main-analysis MD, 95% CrI | Post-exclusion MD, 95% CrI | Change in significance | Clear change in ranking | Interpretation |
| --- | --- | --- | --- | --- | --- | --- | --- | --- |
| DOMS | 24 h | Xia 2022 | LIFU | -34.54 [-62.45, -6.61] | Not estimable, LIFU node removed | NA | NA | Xia 2022 was the only study contributing to the LIFU node; after its exclusion, the LIFU node was removed from the network and a within-node sensitivity comparison could not be performed. |
| DOMS | 48 h | Xia 2022 | LIFU | -65.81 [-92.30, -39.66] | Not estimable, LIFU node removed | NA | NA | Xia 2022 was the only study contributing to the LIFU node; after its exclusion, the LIFU node was removed from the network and a within-node sensitivity comparison could not be performed. |
| DOMS | 24 h | Antonialli 2014 | PHO | -18.20 [-32.91, -3.72] | -12.84 [-29.14, 2.94] | Yes | Yes / possibly lower | After exclusion of Antonialli 2014, the PHO point estimate for DOMS at 24 h remained below zero, but the 95% CrI crossed 0 and the effect changed from significant to non-significant, indicating that the DOMS 24 h result for PHO was sensitive to Antonialli 2014. |
| DOMS | 48 h | Xue 2023 | KT | -4.63 [-16.49, 7.36] | -1.56 [-14.32, 11.93] | No; both estimates were non-significant | No clear change | After exclusion of Xue 2023, the KT point estimate for DOMS at 48 h remained below zero, but the effect estimate decreased and the credible interval still crossed 0, indicating no stable significant advantage for KT. |
| DOMS | 48 h | Xue 2023 | COMP | -3.31 [-19.38, 12.08] | 2.20 [-19.73, 21.24] | No; both estimates were non-significant | Possibly lower | After exclusion of Xue 2023, the direction of the COMP versus CON/PBO comparison changed, but the credible intervals crossed 0 both before and after exclusion, indicating that the direction of this estimate was sensitive to study exclusion. |
| CK | 24 h | Antonialli 2014 | PHO | -199.48 [-329.27, -80.63] | -91.27 [-151.87, -38.59] | No; remained significant | PHO ranking decreased / effect attenuated | After exclusion of Antonialli 2014, PHO was associated with lower CK levels at 24 h than CON/PBO, with a 95% CrI below zero, although the effect size was substantially attenuated. This indicates that the CK 24 h result for PHO was partly influenced by Antonialli 2014, while the direction of effect and statistical significance remained stable. |
| CK | 48 h | Vanin 2016 | LLLT | -499.96 [-831.59, -203.59] | -892.35 [-1465.47, -306.52] | No; remained significant | No clear change | After exclusion of Vanin 2016, LLLT was associated with lower CK levels at 48 h than CON/PBO, with a 95% CrI below zero, and the direction of effect was preserved, indicating that the LLLT result did not depend entirely on Vanin 2016. |
| CK | 48 h | Qu 2020 | CWT | -48.67 [-386.23, 291.26] | Not estimable, CWT node removed | NA | NA | Qu 2020 was the key or sole source of evidence for the CWT node in the CK 48 h network; after its exclusion, the CWT node was removed from the network and a within-node sensitivity comparison could not be performed. |
| CK | 48 h | Qu 2020 | WBC | -168.96 [-506.92, 172.02] | Not estimable, WBC node removed | NA | NA | Qu 2020 was the key or sole source of evidence for the WBC node in the CK 48 h network; after its exclusion, the WBC node was removed from the network and the post-exclusion effect estimate could not be calculated. |

6.3 Robustness assessment for sparse and single-study findings

6.3.1 Statistical significance versus robustness

A statistically non-null network estimate was not considered synonymous with robust evidence when the treatment node was supported by a single small study or when the estimate was materially altered by removal of an influential dataset. Robustness was judged by considering the number of contributing studies, leave-one-out sensitivity results, effect direction and 95% CrI, prediction intervals, and the stability of the treatment node within the network.

Supplementary Table 19A. Robustness interpretation of selected sparse or influential findings.

| **Outcome/time** | **Node** | **Sensitivity finding** | **Robustness interpretation** |
| --- | --- | --- | --- |
| DOMS 24 h | LIFU | Xia 2022 was the only contributing study; excluding it removed the LIFU node. | Estimated reduction in DOMS in the primary NMA, but not robust evidence of superiority. |
| DOMS 48 h | LIFU | Xia 2022 was the only contributing study; excluding it removed the LIFU node. | Large estimated reduction in DOMS, but completely dependent on one small study; independent replication is required. |
| DOMS 24 h | PHO | Excluding Antonialli 2014 changed MD from -18.20 (-32.91 to -3.72) to -12.84 (-29.14 to 2.94). | Direction retained, but the CrI crossed the null; the primary non-null estimate is sensitive to this study. |
| CK 24 h | PHO | Excluding Antonialli 2014 changed MD from -199.48 (-329.27 to -80.63) to -91.27 (-151.87 to -38.59). | Non-null CrI retained, but effect magnitude was substantially attenuated. |
| CK 48 h | LLLT | Excluding Vanin 2016 yielded MD -892.35 (-1465.47 to -306.52). | Direction and non-null CrI were retained; this finding was relatively more stable to study removal. |
| CK 48 h | CWT/WBC | Excluding Qu 2020 removed the CWT and WBC nodes from the network. | No robust within-node conclusion is possible because these nodes depended on that dataset. |

Note. A non-null 95% CrI indicates statistical evidence within the fitted network but does not, by itself, establish robust clinical superiority. Node removal after exclusion of a single study indicates complete dependence of that node on the excluded dataset.

6.3.2 Interpretation of SUCRA rankings

SUCRA values were interpreted only as relative ranking summaries. In sparse networks, a treatment can rank highly because of a large estimate from a single or small study. Rankings were therefore considered together with effect estimates, 95% CrIs, the number of contributing studies, leave-one-out sensitivity findings, prediction intervals, and confidence in the evidence, and were not used alone to claim clinical superiority.

6.4 Sensitivity analyses excluding small-sample studies

6.4.1 Sensitivity analysis excluding small-sample studies for DOMS

Supplementary Figure 6. Sensitivity analyses excluding small-sample studies for DOMS at 24 h and 48 h.

6.4.2 Sensitivity analysis excluding small-sample studies for CK

Supplementary Figure 7. Sensitivity analyses excluding small-sample studies for CK at 24 h and 48 h.

6.5 Sensitivity analyses excluding randomized crossover trials

Ten randomized crossover trials were included in the review: Duffield 2010, Fonseca 2016, Pruscino 2013, Qu 2020, Gatterer 2015, Kirmizigil 2020, McLoughlin 2004, Szymura 2016, Krueger 2019, and Pinto 2022. In the primary NMA, crossover trials were entered using the reported condition-specific means, standard deviations, and sample sizes as marginal arm-level summaries. The extracted NMA dataset did not contain paired-difference standard deviations or within-participant correlation coefficients for the required outcome/time-point combinations; no correlation coefficient was imputed, and within-participant covariance was therefore not explicitly modelled in the arm-based MetaInsight analysis. No additional statistical adjustment for period or carryover effects was applied because the information required for such modelling was not consistently reported. To assess the influence of this limitation, all randomized crossover trials contributing to the DOMS and CK networks were excluded and the networks were re-estimated. The crossover-specific risk-of-bias assessment separately considered intervention-sequence randomization, washout, period effects, and carryover effects (Supplementary Table 5).

6.5.1 Sensitivity analysis excluding randomized crossover trials for DOMS

Supplementary Figure 8. Sensitivity analyses excluding randomized crossover trials for DOMS at 24 h and 48 h.

Five crossover trials contributed to the DOMS networks (Qu 2020, Gatterer 2015, Kirmizigil 2020, McLoughlin 2004, and Szymura 2016). After their exclusion, 17 studies/36 arms remained at 24 h and 18 studies/38 arms at 48 h. LIFU and PHO were associated with lower DOMS than CON/PBO at both time points: at 24 h, LIFU MD = -34.52 (95% CrI -64.98 to -3.76) and PHO MD = -18.12 (-33.93 to -2.59); at 48 h, LIFU MD = -65.8 (-96.7 to -34.9) and PHO MD = -19.7 (-35.9 to -3.93). Several nodes supported entirely by crossover evidence were no longer estimable after exclusion, so ranking changes were interpreted cautiously.

6.5.2 Sensitivity analysis excluding randomized crossover trials for CK

Supplementary Figure 9. Sensitivity analyses excluding randomized crossover trials for CK at 24 h and 48 h.

For CK, four crossover trials contributed at 24 h (Duffield 2010, Fonseca 2016, Pruscino 2013, and Qu 2020) and four at 48 h (Fonseca 2016, Pruscino 2013, Qu 2020, and Pinto 2022). After exclusion, 17 studies/40 arms remained at 24 h and 17 studies/41 arms at 48 h. At 24 h, PHO was associated with lower CK levels [MD = -205 (95% CrI -365 to -55.6)], whereas the LLLT point estimate remained below zero but its CrI crossed the null [-249 (-566 to 67.0)]. At 48 h, both LLLT [-515 (-907 to -170)] and PHO [-292 (-519 to -74.2)] had 95% CrIs below zero. Thus, exclusion of crossover evidence did not produce a systematic reversal of the principal CK effect directions, although precision was reduced for some comparisons.

Supplementary Table 19B. Design-specific sensitivity analyses excluding randomized crossover trials.

| **Outcome/time** | **Studies/arms** | **Dbar / pD / DIC** | **τ (95% CrI)** | **Key effects vs CON/PBO after exclusion** | **Interpretation** |
| --- | --- | --- | --- | --- | --- |
| DOMS 24 h | 17 / 36 | 36.109 / 34.515 / 70.624 | 14.30 (7.97 to 24.94) | LIFU: -34.52 (-64.98 to -3.76); PHO: -18.12 (-33.93 to -2.59) | Both 95% CrIs remained below zero; some crossover-only nodes were removed. |
| DOMS 48 h | 18 / 38 | 38.787 / 36.527 / 75.313 | 14.67 (8.35 to 25.43) | LIFU: -65.8 (-96.7 to -34.9); PHO: -19.7 (-35.9 to -3.93) | Both 95% CrIs remained below zero; some crossover-only nodes were removed. |
| CK 24 h | 17 / 40 | 39.896 / 38.339 / 78.235 | 147.48 (84.03 to 252.88) | LLLT: -249 (-566 to 67.0); PHO: -205 (-365 to -55.6) | The PHO 95% CrI remained below zero; the LLLT point estimate was negative but its CrI included zero. |
| CK 48 h | 17 / 41 | 40.639 / 38.436 / 79.075 | 214.46 (128.39 to 360.87) | LLLT: -515 (-907 to -170); PHO: -292 (-519 to -74.2) | Both 95% CrIs remained below zero after exclusion. |

Note. Dbar, posterior mean deviance; pD, effective number of parameters; DIC, deviance information criterion; τ, between-study standard deviation; MD, mean difference; CrI, credible interval. Model-fit values are reported descriptively for the reduced datasets and should not be interpreted as formal model-selection comparisons with the primary analyses because the underlying study sets differ.

# 7. Heterogeneity and prediction intervals

Network-level heterogeneity was summarized using tau-squared (τ²), tau (τ), and I². Prediction intervals were calculated for each intervention versus CON/PBO by combining the network meta-analysis mean difference and its uncertainty with the heterogeneity summary reported in Supplementary Table 20. These descriptive heterogeneity statistics and prediction intervals are reported separately from the posterior between-study standard deviations of the primary Bayesian models in Supplementary Table 32 and should not be interpreted as identical quantities. Because several treatment nodes were informed by few studies, prediction intervals were treated as supplementary uncertainty summaries and were not used to override the primary Bayesian 95% CrIs, certainty assessments, or conclusions.

Supplementary Table 20. Descriptive network-level heterogeneity statistics used for supplementary prediction-interval summaries.

Note. These descriptive heterogeneity statistics are reported separately from the posterior between-study standard deviations from the primary Bayesian NMA (Supplementary Table 32).

| Outcome | Studies | Treatments | Contrasts | τ² | τ | I² (%) | Q (df) |
| --- | --- | --- | --- | --- | --- | --- | --- |
| DOMS at 24 h | 22 | 14 | 26 | 146.00 | 12.08 | 86.59 | 96.97 (13) |
| DOMS at 48 h | 23 | 14 | 27 | 143.17 | 11.97 | 94.20 | 241.29 (14) |
| CK at 24 h | 21 | 15 | 29 | 11183.69 | 105.75 | 85.87 | 106.14 (15) |
| CK at 48 h | 21 | 14 | 30 | 26300.92 | 162.18 | 92.42 | 224.28 (17) |
| IL-6 at 24 h | 10 | 13 | 15 | 0.13 | 0.35 | 78.49 | 13.95 (3) |
| IL-6 at 48 h | 8 | 11 | 13 | 0.00 | 0.00 | 73.58 | 11.36 (3) |

Supplementary Table 21. Prediction intervals for DOMS at 24 h.

| Treatment | Comparator | MD [95% CrI] | Prediction interval | Interpretation |
| --- | --- | --- | --- | --- |
| WBC | CON/PBO | -38.92 [-69.08, -9.15] | -77.11 to -0.73 | PI does not cross 0 |
| LIFU | CON/PBO | -34.54 [-62.45, -6.61] | -71.15 to 2.07 | PI crosses 0 |
| CWT | CON/PBO | -26.92 [-56.89, 2.58] | -64.93 to 11.09 | PI crosses 0 |
| PHO | CON/PBO | -18.20 [-32.91, -3.72] | -46.02 to 9.62 | PI crosses 0 |
| ES | CON/PBO | -15.55 [-38.87, 7.64] | -48.74 to 17.64 | PI crosses 0 |
| COMP | CON/PBO | -10.03 [-26.34, 5.87] | -38.67 to 18.61 | PI crosses 0 |
| CWI | CON/PBO | -8.92 [-38.92, 20.66] | -46.98 to 29.14 | PI crosses 0 |
| CRY | CON/PBO | -8.59 [-28.40, 10.72] | -39.31 to 22.13 | PI crosses 0 |
| LLLT | CON/PBO | -7.21 [-30.97, 17.01] | -40.92 to 26.50 | PI crosses 0 |
| KT | CON/PBO | -6.20 [-20.06, 7.91] | -33.70 to 21.30 | PI crosses 0 |
| VIB | CON/PBO | -4.69 [-24.63, 15.73] | -35.80 to 26.42 | PI crosses 0 |
| AC | CON/PBO | -2.13 [-24.98, 18.56] | -34.30 to 30.04 | PI crosses 0 |
| STR | CON/PBO | -0.03 [-28.40, 28.26] | -36.96 to 36.90 | PI crosses 0 |

Supplementary Table 22. Prediction intervals for DOMS at 48 h.

| Treatment | Comparator | MD [95% CrI] | Prediction interval | Interpretation |
| --- | --- | --- | --- | --- |
| LIFU | CON/PBO | -65.81 [-92.30, -39.66] | -101.06 to -30.56 | PI does not cross 0 |
| WBC | CON/PBO | -40.03 [-67.74, -12.44] | -76.29 to -3.77 | PI does not cross 0 |
| CWT | CON/PBO | -28.08 [-56.27, -0.26] | -64.61 to 8.45 | PI crosses 0 |
| PHO | CON/PBO | -19.46 [-33.55, -5.99] | -46.66 to 7.74 | PI crosses 0 |
| ES | CON/PBO | -13.64 [-37.64, 10.22] | -47.15 to 19.87 | PI crosses 0 |
| CRY | CON/PBO | -9.88 [-28.93, 8.48] | -39.88 to 20.12 | PI crosses 0 |
| CWI | CON/PBO | -8.06 [-35.87, 19.70] | -44.42 to 28.30 | PI crosses 0 |
| VIB | CON/PBO | -6.56 [-24.97, 12.16] | -36.47 to 23.35 | PI crosses 0 |
| STR | CON/PBO | -5.10 [-32.95, 23.22] | -41.69 to 31.49 | PI crosses 0 |
| AC | CON/PBO | -4.88 [-26.46, 14.81] | -36.12 to 26.36 | PI crosses 0 |
| KT | CON/PBO | -4.63 [-16.49, 7.36] | -30.94 to 21.68 | PI crosses 0 |
| COMP | CON/PBO | -3.31 [-19.38, 12.08] | -31.55 to 24.93 | PI crosses 0 |
| LLLT | CON/PBO | -2.16 [-26.49, 22.86] | -36.20 to 31.88 | PI crosses 0 |

Supplementary Table 23. Prediction intervals for CK at 24 h.

| Treatment | Comparator | MD [95% CrI] | Prediction interval | Interpretation |
| --- | --- | --- | --- | --- |
| LLLT | CON/PBO | -249.70 [-502.23, -1.08] | -574.89 to 75.49 | PI crosses 0 |
| PHO | CON/PBO | -199.48 [-329.27, -80.63] | -441.18 to 42.22 | PI crosses 0 |
| WBC | CON/PBO | -196.56 [-423.81, 30.34] | -504.01 to 110.89 | PI crosses 0 |
| LIFU | CON/PBO | -119.76 [-366.03, 129.34] | -442.73 to 203.21 | PI crosses 0 |
| VIB | CON/PBO | -109.17 [-246.21, 27.22] | -357.47 to 139.13 | PI crosses 0 |
| CWT | CON/PBO | -100.84 [-326.74, 125.49] | -407.58 to 205.90 | PI crosses 0 |
| Massage | CON/PBO | -95.54 [-302.75, 111.19] | -388.46 to 197.38 | PI crosses 0 |
| CWI | CON/PBO | -69.16 [-209.30, 69.09] | -318.84 to 180.52 | PI crosses 0 |
| ES | CON/PBO | -64.14 [-272.28, 141.85] | -357.12 to 228.84 | PI crosses 0 |
| STR | CON/PBO | -33.86 [-156.92, 87.67] | -274.52 to 206.80 | PI crosses 0 |
| CRY | CON/PBO | -33.66 [-181.53, 116.66] | -288.99 to 221.67 | PI crosses 0 |
| COMP | CON/PBO | -22.00 [-197.51, 150.41] | -292.60 to 248.60 | PI crosses 0 |
| SW | CON/PBO | 5.88 [-238.52, 252.05] | -315.26 to 327.02 | PI crosses 0 |
| KT | CON/PBO | 16.47 [-204.21, 236.75] | -286.14 to 319.08 | PI crosses 0 |

Supplementary Table 24. Prediction intervals for CK at 48 h.

| Treatment | Comparator | MD [95% CrI] | Prediction interval | Interpretation |
| --- | --- | --- | --- | --- |
| LLLT | CON/PBO | -499.96 [-831.59, -203.59] | -946.76 to -53.16 | PI does not cross 0 |
| PHO | CON/PBO | -252.50 [-420.45, -87.47] | -611.33 to 106.33 | PI crosses 0 |
| WBC | CON/PBO | -168.96 [-506.92, 172.02] | -634.02 to 296.10 | PI crosses 0 |
| SW | CON/PBO | -60.15 [-429.57, 311.05] | -548.17 to 427.87 | PI crosses 0 |
| CRY | CON/PBO | -53.36 [-259.44, 147.75] | -430.84 to 324.12 | PI crosses 0 |
| COMP | CON/PBO | -52.06 [-385.61, 274.21] | -510.18 to 406.06 | PI crosses 0 |
| CWT | CON/PBO | -48.67 [-386.23, 291.26] | -513.20 to 415.86 | PI crosses 0 |
| Massage | CON/PBO | -47.11 [-362.06, 260.72] | -492.08 to 397.86 | PI crosses 0 |
| ES | CON/PBO | -39.58 [-350.40, 267.84] | -482.97 to 403.81 | PI crosses 0 |
| STR | CON/PBO | -35.69 [-245.28, 171.88] | -415.88 to 344.50 | PI crosses 0 |
| CWI | CON/PBO | -24.14 [-229.12, 178.32] | -401.68 to 353.40 | PI crosses 0 |
| VIB | CON/PBO | -16.48 [-220.79, 186.80] | -394.06 to 361.10 | PI crosses 0 |
| KT | CON/PBO | 16.56 [-229.97, 267.29] | -386.99 to 420.11 | PI crosses 0 |

Supplementary Table 25. Prediction intervals for IL-6 at 24 h.

| Treatment | Comparator | MD [95% CrI] | Prediction interval | Interpretation |
| --- | --- | --- | --- | --- |
| CWI | CON/PBO | -97.26 [-186.18, -15.25] | -182.73 to -11.79 | PI does not cross 0 |
| ES | CON/PBO | -91.35 [-177.45, -11.83] | -174.16 to -8.54 | PI does not cross 0 |
| LLLT | CON/PBO | -64.98 [-164.23, 31.43] | -162.81 to 32.85 | PI crosses 0 |
| Massage | CON/PBO | -52.35 [-140.69, 29.20] | -137.30 to 32.60 | PI crosses 0 |
| STR | CON/PBO | -42.50 [-131.53, 38.77] | -127.65 to 42.65 | PI crosses 0 |
| LIFU | CON/PBO | -8.04 [-95.61, 79.85] | -95.77 to 79.69 | PI crosses 0 |
| VIB | CON/PBO | -7.64 [-73.16, 30.07] | -59.26 to 43.98 | PI crosses 0 |
| PHO | CON/PBO | -3.81 [-91.47, 84.24] | -91.67 to 84.05 | PI crosses 0 |
| KT | CON/PBO | -0.88 [-84.06, 81.09] | -83.46 to 81.70 | PI crosses 0 |
| COMP | CON/PBO | -0.77 [-62.88, 61.97] | -63.20 to 61.66 | PI crosses 0 |
| CRY | CON/PBO | -0.51 [-88.93, 88.10] | -89.03 to 88.01 | PI crosses 0 |
| WBC | CON/PBO | 0.19 [-88.80, 88.30] | -88.36 to 88.74 | PI crosses 0 |

Supplementary Table 26. Prediction intervals for IL-6 at 48 h.

| Treatment | Comparator | MD [95% CrI] | Prediction interval | Interpretation |
| --- | --- | --- | --- | --- |
| LLLT | CON/PBO | -79.34 [-153.29, -5.12] | -153.43 to -5.26 | PI does not cross 0 |
| CWI | CON/PBO | -66.19 [-130.87, -17.27] | -122.99 to -9.39 | PI does not cross 0 |
| Massage | CON/PBO | -26.72 [-91.24, 18.72] | -81.70 to 28.26 | PI crosses 0 |
| ES | CON/PBO | -22.22 [-88.27, 26.49] | -79.60 to 35.16 | PI crosses 0 |
| STR | CON/PBO | -21.48 [-87.28, 26.65] | -78.45 to 35.48 | PI crosses 0 |
| VIB | CON/PBO | -5.26 [-46.15, 21.89] | -39.28 to 28.76 | PI crosses 0 |
| PHO | CON/PBO | -2.53 [-61.31, 55.09] | -60.73 to 55.67 | PI crosses 0 |
| KT | CON/PBO | -0.87 [-55.66, 54.17] | -55.78 to 54.05 | PI crosses 0 |
| CRY | CON/PBO | -0.24 [-58.56, 57.84] | -58.44 to 57.96 | PI crosses 0 |
| COMP | CON/PBO | 0.08 [-42.34, 42.23] | -42.20 to 42.37 | PI crosses 0 |

**8. Exploratory network meta-regression**

8.1 Rationale and covariate coding

Exploratory study-level covariates were coded to assess whether selected design, population, and exercise-induced muscle damage characteristics could explain between-study heterogeneity in the network meta-analyses.

Supplementary Table 27. Coding rules for exploratory network meta-regression.

| Covariate | Coding | Rationale |
| --- | --- | --- |
| InductionType | 0 = localized eccentric/isokinetic model; 1 = running, jumping, sprint, sport-specific or whole-body/high-intensity model | Exercise-induced muscle damage models may differ in systemic load and recovery response. |
| MuscleRegion | 0 = upper limb/local upper muscle; 1 = lower limb, whole-body, or systemic intervention/body region | Intervention/body region may modify treatment effects and heterogeneity. |
| TrainingStatus | 0 = untrained/healthy/recreational; 1 = trained/athletic/sport-specific participants | Training status may influence muscle damage and recovery kinetics. |
| Design | 0 = parallel-group randomized trial; 1 = randomized crossover trial | Study design may influence precision and within-participant variation. |
| HighRoB | 0 = low or some concerns; 1 = high overall risk of bias | Risk of bias may modify apparent treatment effects. |
| SmallSample | 0 = total N >= 15; 1 = total N < 15 | Small studies may be more unstable and contribute to heterogeneity. |

Supplementary Table 28. Study-level covariates used in exploratory network meta-regression.

| Study | InductionType | MuscleRegion | TrainingStatus | Design | HighRoB | SmallSample | Coding notes |
| --- | --- | --- | --- | --- | --- | --- | --- |
| Antonialli_2014 | 0 | 1 | 0 | 0 | 0 | 0 | NA |
| Boobphachart_2017 | 0 | 1 | 0 | 0 | 0 | 0 | NA |
| Cheng_2022 | 1 | 1 | 1 | 0 | 1 | 0 | NA |
| Cheng_2025 | 1 | 1 | 1 | 0 | 1 | 0 | NA |
| De_Marchi_2017 | 0 | 0 | 0 | 0 | 0 | 0 | Physically active but not clearly athlete/trained; coded 0 conservatively. |
| Duffield_2010 | 1 | 1 | 1 | 1 | 0 | 1 | NA |
| Fonseca_2016 | 1 | 1 | 1 | 1 | 1 | 1 | NA |
| Guilhem_2013 | 0 | 0 | 0 | 0 | 1 | 0 | NA |
| Kim_2017 | 0 | 0 | 0 | 0 | 1 | 0 | NA |
| Machado_2020 | 0 | 1 | 0 | 0 | 0 | 0 | NA |
| Oakley_2013 | 0 | 1 | 0 | 0 | 0 | 0 | Mixed trained/untrained population; coded 0 conservatively per prespecified rule. |
| Pruscino_2013 | 1 | 1 | 1 | 1 | 0 | 1 | NA |
| Qu_2020 | 1 | 1 | 1 | 1 | 1 | 1 | NA |
| Eston_1999 | 0 | 0 | 0 | 0 | 0 | 0 | NA |
| Torres_2013 | 0 | 1 | 0 | 0 | 1 | 0 | NA |
| Wei_2025 | 1 | 1 | 0 | 0 | 0 | 0 | NA |
| de_Paiva_2016 | 0 | 1 | 0 | 0 | 1 | 0 | NA |
| Hazar_Kanik_2018 | 1 | 1 | 0 | 0 | 1 | 0 | NA |
| Douris_2006 | 0 | 0 | 0 | 0 | 0 | 0 | NA |
| Fleckenstein_2016 | 0 | 0 | 0 | 0 | 0 | 0 | NA |
| Gatterer_2015 | 1 | 1 | 1 | 1 | 1 | 1 | NA |
| Itoh_2008 | 0 | 0 | 0 | 0 | 1 | 0 | NA |
| Kirmizigil_2020 | 1 | 1 | 1 | 1 | 1 | 0 | NA |
| Lee_2015 | 0 | 0 | 0 | 0 | 1 | 0 | NA |
| McLoughlin_2004 | 0 | 0 | 0 | 1 | 1 | 1 | NA |
| Cerqueira_2015 | 0 | 0 | 0 | 0 | 1 | 1 | NA |
| Szymura_2016 | 1 | 1 | 0 | 1 | 1 | 1 | NA |
| Xue_2023 | 1 | 1 | 0 | 0 | 1 | 0 | NA |
| Krueger_2019 | 1 | 1 | 1 | 1 | 0 | 1 | Characteristics table year differs from risk-of-bias/data files; harmonized as Krueger_2019 and |
| Kay_2018 | 1 | 1 | 0 | 0 | 0 | 0 | NA |
| Vanin_2016 | 0 | 1 | 1 | 0 | 0 | 1 | NA |
| Xia_2022 | 1 | 1 | 0 | 0 | 1 | 0 | NA |
| Sudhakar_2023 | 0 | 0 | 1 | 0 | 0 | 0 | NA |
| Pinto_2022 | 1 | 1 | 1 | 1 | 0 | 1 | NA |
| Baroni_2010 | 0 | 1 | 0 | 0 | 0 | 0 | Physically active but not clearly athlete/trained; coded 0 conservatively. |

8.2 Meta-regression methods

Exploratory network meta-regression analyses were conducted to examine whether selected study-level characteristics were associated with variation in treatment effects. The fitted analyses comprised 33 univariable shared-β models: three for DOMS at 24 h and six for each of the remaining five outcome/time-point networks. The corresponding network study counts were 22 for DOMS 24 h, 23 for DOMS 48 h, 21 for CK 24 h, 21 for CK 48 h, 10 for IL-6 24 h, and 8 for IL-6 48 h. Each model included one covariate only and used the studies available in the corresponding network. This one-covariate-at-a-time specification limited parameter burden and avoided fitting a multivariable meta-regression to relatively small networks. Random-effects Bayesian models with a shared regression coefficient were fitted using MetaInsight, and results were interpreted jointly using β estimates and 95% CrIs, DIC, residual deviance, and heterogeneity standard deviation (τ). No formal multiplicity adjustment was applied across the 33 exploratory models; accordingly, isolated non-null coefficients were interpreted in the context of the overall pattern of model-fit and heterogeneity results.

For transparency, the number of fitted models and contributing studies for each network is reported with Supplementary Table 29. The larger DOMS and CK networks provided more information for exploratory assessment of effect modification than the smaller IL-6 networks; precision was judged directly from the posterior 95% CrIs together with model-fit and heterogeneity changes rather than from a post-hoc power threshold.

8.3 Summary of meta-regression results

Across DOMS and CK outcomes, the β coefficients for the fitted covariates generally had wide 95% CrIs overlapping zero, and covariate adjustment did not consistently improve DIC or reduce heterogeneity. For DOMS 24 h, the three fitted models (InductionType, MuscleRegion, and TrainingStatus) all had 95% CrIs overlapping zero; although the TrainingStatus model reduced τ from 13.26 to 12.70, DIC did not improve. At IL-6 48 h, several covariates yielded non-null β estimates and lower DIC values, but these models were based on eight studies and heterogeneity increased rather than decreased. These findings were therefore interpreted as exploratory signals rather than stable effect-modification patterns.

Supplementary Table 29. Summary of exploratory network meta-regression results.

| Outcome | Time point | Covariate | DIC unadjusted | DIC adjusted | ΔDIC | Heterogeneity SD unadjusted | Heterogeneity SD adjusted | β | 95% CrI | Interpretation |
| --- | --- | --- | --- | --- | --- | --- | --- | --- | --- | --- |
| DOMS | 24 h | InductionType | 93.97 | 94.22 | 0.25 | 13.26 | 13.78 | -3.70 | -21.60 to 18.38 | No clear treatment–covariate interaction. |
| DOMS | 24 h | MuscleRegion | 93.97 | 94.11 | 0.13 | 13.26 | 13.69 | -4.40 | -19.90 to 11.78 | No clear treatment–covariate interaction. |
| DOMS | 24 h | TrainingStatus | 93.97 | 94.23 | 0.26 | 13.26 | 12.70 | 12.98 | -7.77 to 33.77 | No clear treatment–covariate interaction. |
| DOMS | 48 h | InductionType | 98.39 | 99.39 | 1.00 | 12.64 | 18.98 | 11.96 | -42.91 to 198.40 | No clear treatment–covariate interaction. |
| DOMS | 48 h | MuscleRegion | 98.39 | 99.08 | 0.69 | 12.64 | 18.33 | 15.05 | -16.90 to 189.62 | No clear treatment–covariate interaction. |
| DOMS | 48 h | TrainingStatus | 98.39 | 98.90 | 0.51 | 12.64 | 19.18 | 39.14 | -9.85 to 202.89 | No clear treatment–covariate interaction. |
| DOMS | 48 h | Design | 98.39 | 98.28 | -0.11 | 12.64 | 13.48 | 7.06 | -19.11 to 35.77 | No clear treatment–covariate interaction. |
| DOMS | 48 h | HighRoB | 98.39 | 98.68 | 0.29 | 12.64 | 15.03 | 7.43 | -25.18 to 132.02 | No clear treatment–covariate interaction. |
| DOMS | 48 h | SmallSample | 98.39 | 97.40 | -0.99 | 12.64 | 12.32 | 14.26 | -10.67 to 36.21 | No clear treatment–covariate interaction. |
| CK | 24 h | InductionType | 96.61 | 98.58 | 1.96 | 118.31 | 252.41 | 441.82 | -392.01 to 1795.00 | No clear treatment–covariate interaction. |
| CK | 24 h | MuscleRegion | 96.61 | 99.47 | 2.85 | 118.31 | 244.20 | 446.12 | -335.50 to 1680.40 | No clear treatment–covariate interaction. |
| CK | 24 h | TrainingStatus | 96.61 | 98.36 | 1.75 | 118.31 | 244.27 | 473.53 | -209.86 to 1624.89 | No clear treatment–covariate interaction. |
| CK | 24 h | Design | 96.61 | 98.11 | 1.50 | 118.31 | 213.32 | 374.57 | -173.25 to 1547.47 | No clear treatment–covariate interaction. |
| CK | 24 h | HighRoB | 96.61 | 98.81 | 2.19 | 118.31 | 262.97 | 391.70 | -340.42 to 1651.20 | No clear treatment–covariate interaction. |
| CK | 24 h | SmallSample | 96.61 | 98.11 | 1.50 | 118.31 | 221.19 | 414.07 | -250.13 to 1643.70 | No clear treatment–covariate interaction. |
| CK | 48 h | InductionType | 98.08 | 99.50 | 1.41 | 180.22 | 431.02 | 850.23 | -438.60 to 3128.12 | No clear treatment–covariate interaction. |
| CK | 48 h | MuscleRegion | 98.08 | 99.92 | 1.84 | 180.22 | 420.87 | 867.61 | -424.30 to 3172.96 | No clear treatment–covariate interaction. |
| CK | 48 h | TrainingStatus | 98.08 | 99.33 | 1.25 | 180.22 | 428.61 | 817.41 | -423.90 to 3048.91 | No clear treatment–covariate interaction. |
| CK | 48 h | Design | 98.08 | 99.31 | 1.22 | 180.22 | 389.84 | 785.57 | -302.30 to 3131.21 | No clear treatment–covariate interaction. |
| CK | 48 h | HighRoB | 98.08 | 100.11 | 2.03 | 180.22 | 454.43 | 853.13 | -419.70 to 3260.42 | No clear treatment–covariate interaction. |
| CK | 48 h | SmallSample | 98.08 | 99.10 | 1.02 | 180.22 | 406.15 | 803.69 | -339.90 to 3136.44 | No clear treatment–covariate interaction. |
| IL-6 | 24 h | InductionType | 53.13 | 53.43 | 0.30 | 30.02 | 28.52 | 181.40 | -281.10 to 480.82 | No clear treatment–covariate interaction. |
| IL-6 | 24 h | MuscleRegion | 53.13 | 53.09 | -0.04 | 30.02 | 31.61 | 192.23 | -253.16 to 476.97 | No clear treatment–covariate interaction. |
| IL-6 | 24 h | TrainingStatus | 53.13 | 50.07 | -3.06 | 30.02 | 105.53 | 173.13 | -258.95 to 456.30 | No clear treatment–covariate interaction. |
| IL-6 | 24 h | Design | 53.13 | 49.91 | -3.22 | 30.02 | 97.54 | 173.43 | -257.56 to 469.52 | No clear treatment–covariate interaction. |
| IL-6 | 24 h | HighRoB | 53.13 | 50.09 | -3.04 | 30.02 | 105.75 | 166.73 | -272.04 to 447.10 | No clear treatment–covariate interaction. |
| IL-6 | 24 h | SmallSample | 53.13 | 50.02 | -3.11 | 30.02 | 98.26 | 173.12 | -264.48 to 455.34 | No clear treatment–covariate interaction. |
| IL-6 | 48 h | InductionType | 44.73 | 44.28 | -0.46 | 20.19 | 21.22 | 182.33 | 94.41 to 273.42 | Non-zero beta, but fit/heterogeneity not both improved; sparse exploratory signal. |
| IL-6 | 48 h | MuscleRegion | 44.73 | 44.70 | -0.03 | 20.19 | 20.26 | 174.69 | 63.00 to 278.95 | Non-zero beta, but fit/heterogeneity not both improved; sparse exploratory signal. |
| IL-6 | 48 h | TrainingStatus | 44.73 | 42.06 | -2.67 | 20.19 | 67.75 | 169.34 | 66.56 to 260.44 | Non-zero beta, but fit/heterogeneity not both improved; sparse exploratory signal. |
| IL-6 | 48 h | Design | 44.73 | 41.97 | -2.76 | 20.19 | 65.05 | 178.00 | 64.70 to 271.64 | Non-zero beta, but fit/heterogeneity not both improved; sparse exploratory signal. |
| IL-6 | 48 h | HighRoB | 44.73 | 42.05 | -2.68 | 20.19 | 70.43 | 178.97 | 92.15 to 279.94 | Non-zero beta, but fit/heterogeneity not both improved; sparse exploratory signal. |
| IL-6 | 48 h | SmallSample | 44.73 | 41.99 | -2.74 | 20.19 | 64.67 | 171.72 | 83.27 to 256.75 | Non-zero beta, but fit/heterogeneity not both improved; sparse exploratory signal. |

8.4 Outcome-specific interpretation

No covariate consistently explained between-study heterogeneity across outcomes. The clearest pattern was the absence of a reproducible treatment–covariate interaction for DOMS and CK. For IL-6, especially at 48 h, several non-null β estimates were observed, but they were not accompanied by a reduction in heterogeneity. Interpretation therefore emphasized coherence across β estimates, 95% CrIs, model fit, and τ rather than isolated non-null coefficients.

8.5 Limitations of meta-regression

The amount of information available for meta-regression varied across networks (8–23 studies), with fewer studies contributing to the IL-6 analyses than to DOMS and CK. The univariable shared-β specification limited the number of additional parameters estimated in each model, but the precision of effect-modification estimates remained dependent on study count and covariate distribution. In addition, 33 exploratory models were examined without multiplicity adjustment, which increases the possibility that isolated non-null coefficients may be false-positive or unstable. Each signal was therefore evaluated in the context of its 95% CrI, DIC, residual deviance, and change in τ, and isolated meta-regression results were not used as the sole basis for the primary treatment-effect conclusions. These analyses were used as complementary assessments of potential effect modification and as a basis for hypotheses to be evaluated in future studies.

Supplementary Table 30. Computational failures or non-convergence in exploratory meta-regression models.

| Outcome | Time point | Covariate | Reason | Handling |
| --- | --- | --- | --- | --- |
| All | All | All | No computational failure or persistent non-convergence was identified. | All completed models were retained for exploratory interpretation. |

9. Bayesian model-fit, convergence, and inconsistency diagnostics

9.1 Bayesian analysis specification

All six networks were analyzed using random-effects Bayesian models for continuous outcomes, with mean difference (MD) as the effect measure and CON/PBO as the reference treatment. Analyses were conducted using MetaInsight v7.1.1. MetaInsight displays normal priors using a mean–variance parameterization, whereas the underlying JAGS code parameterizes normal distributions in terms of mean and precision.

Supplementary Table 31. Bayesian model settings used for the six primary networks.

| **Item** | **Specification** |
| --- | --- |
| Application | MetaInsight web application v7.1.1 |
| Analysis module | Bayesian network meta-analysis |
| Model | Random-effects model |
| Outcome type | Continuous outcome |
| Effect measure | Mean difference (MD) |
| Reference treatment | CON/PBO |
| Seed | 74 |
| MCMC chains | 4 |
| Burn-in iterations | 5,000 |
| Sampling iterations | 20,000 per chain |
| Thinning interval | 1 |
| Retained posterior samples | 80,000 |
| Relative treatment effects prior | Normal(0, 342225) |
| Study-specific intercept prior | Normal(0, 342225) |
| Heterogeneity SD prior | Uniform(0, 39) |
| Normal parameterization | MetaInsight displays Normal(mean, variance); JAGS code uses Normal(mean, precision). |

9.2 Model fit and heterogeneity

Supplementary Table 32. Dataset mapping, Bayesian consistency-model fit, and between-study heterogeneity.

| **Model** | **Outcome/time point** | **Studies** | **Arms/data points** | **Dbar** | **pD** | **DIC** | **Between-study SD (95% CrI)** |
| --- | --- | --- | --- | --- | --- | --- | --- |
| RE Model | DOMS 24 h | 22 | 48 | 47.914 | 45.653 | 93.568 | 13.26 (7.88 to 21.81) |
|  | DOMS 48 h | 23 | 50 | 50.825 | 47.565 | 98.390 | 12.64 (7.63 to 20.76) |
|  | CK 24 h | 21 | 50 | 49.669 | 46.944 | 96.613 | 118.31 (71.18 to 189.75) |
|  | CK 48 h | 21 | 51 | 50.638 | 47.443 | 98.081 | 180.22 (115.77 to 281.35) |
|  | IL-6 24 h | 10 | 25 | 27.605 | 25.525 | 53.130 | 30.02 (0.32 to 101.17) |
|  | IL-6 48 h | 8 | 21 | 23.249 | 21.483 | 44.732 | 20.19 (0.09 to 69.09) |

Supplementary Table 33. MCMC convergence diagnostics for the Bayesian network meta-analysis models

| **Outcome** | **Time** | **Brooks–Gelman–Rubin** | **Trace plots** | **Posterior density** | **Overall** |
| --- | --- | --- | --- | --- | --- |
| DOMS | 24 h | Shrink factors approached 1 for all monitored parameters | Satisfactory mixing without evident trend or persistent chain separation | Stable distributions without evident multimodality | Satisfactory |
| DOMS | 48 h | Shrink factors approached 1 | Satisfactory mixing without evident trend or persistent chain separation | Stable distributions without evident multimodality | Satisfactory |
| CK | 24 h | Shrink factors approached 1 | Satisfactory mixing without evident trend or persistent chain separation | Stable distributions without evident multimodality | Satisfactory |
| CK | 48 h | Shrink factors approached 1 | Satisfactory mixing without evident trend or persistent chain separation | Stable distributions without evident multimodality | Satisfactory |
| IL-6 | 24 h | Shrink factors approached 1 | Satisfactory chain mixing; no persistent separation | Posterior distributions were stable, although some were relatively broad because of sparse evidence | Satisfactory |
| IL-6 | 48 h | Shrink factors approached 1 | Satisfactory chain mixing; no persistent separation | Posterior distributions were stable, although uncertainty was greater in the sparse network | Satisfactory |

9.3 Deviance report for all studies

9.3.1 Residual deviance from NMA model and UME inconsistency model for all studies

Each point represents the contribution of an individual data point to the posterior residual deviance under the consistency model (horizontal axis) and the unrelated mean effects (UME) inconsistency model (vertical axis). Points close to the line of equality indicate similar model fit under the two models. Points above the line indicate lower residual deviance under the consistency model, whereas points below the line indicate lower residual deviance under the UME model. The plot was interpreted together with Dbar, pD, and DIC rather than as a stand-alone test of inconsistency. The limitations of the UME model for multi-arm trials were also considered.[3]

Supplementary Figure 4. Residual deviance from NMA model and UME inconsistency model for all studies.

9.3.2 Per-arm residual deviance for all studies

Each stem represents the posterior residual deviance for an individual study arm. Shorter stems indicate smaller residual deviance and therefore better agreement between the observed data and the fitted model. Study-arm identifiers were used to inspect whether any individual observations contributed disproportionately to overall model misfit.

Supplementary Figure 5. Per-arm residual deviance for all studies.

9.4 Inconsistency assessment

Supplementary Table 34. Global inconsistency assessment by comparing the consistency model with the UME inconsistency model.

| **Outcome/time point** | **Studies** | **Arms/data points** | **Consistency Dbar** | **UME Dbar** | **Consistency pD** | **UME pD** | **Consistency DIC** | **UME DIC** | **ΔDIC (UME − consistency)** | **Interpretation** |
| --- | --- | --- | --- | --- | --- | --- | --- | --- | --- | --- |
| DOMS 24 h | 22 | 48 | 47.914 | 47.915 | 45.653 | 45.653 | 93.568 | 93.568 | +0.000 | No evidence that the UME model improved fit; no important global inconsistency |
| DOMS 48 h | 23 | 50 | 50.825 | 50.697 | 47.565 | 47.545 | 98.390 | 98.242 | -0.148 | UME model showed only trivial improvement; no important global inconsistency |
| CK 24 h | 21 | 50 | 49.669 | 49.722 | 46.944 | 46.990 | 96.613 | 96.713 | +0.100 | No improvement in fit with the UME model; no important global inconsistency |
| CK 48 h | 21 | 51 | 50.638 | 50.759 | 47.443 | 47.661 | 98.081 | 98.420 | +0.339 | No improvement in fit with the UME model; no important global inconsistency |
| IL-6 24 h | 10 | 25 | 27.605 | 27.728 | 25.525 | 25.542 | 53.130 | 53.270 | +0.140 | No improvement in fit with the UME model; no important global inconsistency |
| IL-6 48 h | 8 | 21 | 23.249 | 23.368 | 21.483 | 21.619 | 44.732 | 44.987 | +0.255 | No improvement in fit with the UME model; no important global inconsistency |

Supplementary Table 35. Local inconsistency assessment using node-splitting analyses

| **Outcome** | **Time point** | **Comparison** | **Direct MD (95% CrI)** | **Indirect MD (95% CrI)** | **Network MD (95% CrI)** | **P value** | **Interpretation** |
| --- | --- | --- | --- | --- | --- | --- | --- |
| DOMS | 24 h | KT vs COMP | −5.07 (−33.4 to 23.5) | 10.0 (−16.0 to 37.6) | 3.85 (−15.1 to 23.4) | 0.402 | No statistically detectable local inconsistency |
| DOMS | 24 h | CRY vs PHO | 3.94 (−23.5 to 31.5) | 12.4 (−23.5 to 47.9) | 9.60 (−11.9 to 30.8) | 0.678 | No statistically detectable local inconsistency |
| DOMS | 48 h | KT vs COMP | −3.65 (−29.4 to 21.5) | −3.54 (−26.1 to 22.3) | −1.34 (−18.9 to 17.1) | 0.993 | No statistically detectable local inconsistency |
| DOMS | 48 h | CRY vs PHO | 5.45 (−20.2 to 31.1) | 8.15 (−26.0 to 42.4) | 9.66 (−10.8 to 29.8) | 0.888 | No statistically detectable local inconsistency |
| CK | 24 h | STR vs CWI | −4.96 (−284 to 272) | 69.2 (−193 to 333) | 35.1 (−135 to 207) | 0.670 | No statistically detectable local inconsistency |
| CK | 24 h | VIB vs CWI | −31.1 (−305 to 244) | −48.9 (−329 to 236) | −40.1 (−217 to 140) | 0.916 | No statistically detectable local inconsistency |
| CK | 24 h | VIB vs STR | −26.3 (−299 to 245) | −121 (−377 to 137) | −75.5 (−245 to 95.3) | 0.582 | No statistically detectable local inconsistency |
| CK | 24 h | CRY vs PHO | 102 (−176 to 381) | 238 (−17.5 to 518) | 165 (−3.53 to 350) | 0.448 | No statistically detectable local inconsistency |
| CK | 48 h | VIB vs CWI | −6.15 (−422 to 410) | 18.9 (−397 to 434) | 7.46 (−256 to 270) | 0.928 | No statistically detectable local inconsistency |
| CK | 48 h | STR vs CWI | −4.12 (−419 to 416) | −20.5 (−451 to 405) | −10.7 (−275 to 253) | 0.950 | No statistically detectable local inconsistency |
| CK | 48 h | CRY vs PHO | 130 (−156 to 419) | 310 (−172 to 755) | 199 (−22.0 to 417) | 0.479 | No statistically detectable local inconsistency |
| CK | 48 h | STR vs VIB | 0.848 (−415 to 417) | −39.7 (−471 to 383) | −17.6 (−286 to 246) | 0.886 | No statistically detectable local inconsistency |
| CK | 48 h | KT vs STR | 28.9 (−381 to 444) | 88.8 (−413 to 595) | 53.6 (−229 to 340) | 0.844 | No statistically detectable local inconsistency |
| IL-6 | 24 h | Not estimable | — | — | — | — | No node-splitting comparison was estimable because independent direct and indirect evidence was unavailable for the same treatment contrast |
| IL-6 | 48 h | Not estimable | — | — | — | — | No node-splitting comparison was estimable because independent direct and indirect evidence was unavailable for the same treatment contrast |

10. Transitivity assessment

Transitivity was assessed by examining the distribution of selected potential effect modifiers across direct treatment comparisons. The primary effect modifiers considered were the exercise-induced muscle damage model, intervention/body region, participant training status, and study design, using the coding rules defined in Supplementary Table 27. Because many direct comparisons were represented by a single study, comparison-level distributions are presented for direct contrasts supported by at least two studies; single-study contrasts were considered structurally sparse and were interpreted with additional caution.

The operational distinction between laser-only LLLT and LED-containing PHO is detailed in Supplementary Table 3A. Baroni 2010, Vanin 2016, Antonialli 2014 and de Paiva 2016 used knee-extensor eccentric exercise protocols comprising 75 contractions and treated the quadriceps region. These shared features support the clinical rationale for comparing the represented configurations and were considered alongside differences in participant characteristics and treatment timing. The exploratory meta-regression did not identify a consistent effect-modification pattern for DOMS or CK. These findings were interpreted together with the precision of the models and the observed distribution of potential effect modifiers. Comparisons involving PHO and LLLT therefore concern the treatment configurations represented in these nodes; broader application requires consideration of the light sources, additional components and populations studied.

Supplementary Table 36. Distribution of potential effect modifiers across direct treatment comparisons.

| **Outcome/time** | **Direct comparison** | **Studies** | **Systemic/whole-body induction** | **Lower-limb/whole-body region** | **Trained/athletic** | **Crossover** |
| --- | --- | --- | --- | --- | --- | --- |
| DOMS 24 h | CON/PBO vs KT | 4 | 3/4 (75%) | 3/4 (75%) | 1/4 (25%) | 2/4 (50%) |
| DOMS 24 h | CON/PBO vs PHO | 4 | 0/4 (0%) | 3/4 (75%) | 0/4 (0%) | 0/4 (0%) |
| DOMS 24 h | COMP vs CON/PBO | 3 | 1/3 (33%) | 1/3 (33%) | 0/3 (0%) | 0/3 (0%) |
| DOMS 24 h | AC vs CON/PBO | 2 | 0/2 (0%) | 0/2 (0%) | 0/2 (0%) | 0/2 (0%) |
| DOMS 24 h | CON/PBO vs CRY | 2 | 0/2 (0%) | 1/2 (50%) | 0/2 (0%) | 0/2 (0%) |
| DOMS 24 h | CON/PBO vs ES | 2 | 1/2 (50%) | 1/2 (50%) | 1/2 (50%) | 2/2 (100%) |
| DOMS 24 h | CON/PBO vs LLLT | 2 | 0/2 (0%) | 2/2 (100%) | 1/2 (50%) | 0/2 (0%) |
| DOMS 24 h | CON/PBO vs VIB | 2 | 2/2 (100%) | 2/2 (100%) | 2/2 (100%) | 0/2 (0%) |
| DOMS 48 h | CON/PBO vs KT | 5 | 4/5 (80%) | 4/5 (80%) | 1/5 (20%) | 2/5 (40%) |
| DOMS 48 h | CON/PBO vs PHO | 4 | 0/4 (0%) | 3/4 (75%) | 0/4 (0%) | 0/4 (0%) |
| DOMS 48 h | COMP vs CON/PBO | 3 | 1/3 (33%) | 1/3 (33%) | 0/3 (0%) | 0/3 (0%) |
| DOMS 48 h | AC vs CON/PBO | 2 | 0/2 (0%) | 0/2 (0%) | 0/2 (0%) | 0/2 (0%) |
| DOMS 48 h | CON/PBO vs CRY | 2 | 0/2 (0%) | 1/2 (50%) | 0/2 (0%) | 0/2 (0%) |
| DOMS 48 h | CON/PBO vs ES | 2 | 1/2 (50%) | 1/2 (50%) | 1/2 (50%) | 2/2 (100%) |
| DOMS 48 h | CON/PBO vs LLLT | 2 | 0/2 (0%) | 2/2 (100%) | 1/2 (50%) | 0/2 (0%) |
| DOMS 48 h | CON/PBO vs VIB | 2 | 2/2 (100%) | 2/2 (100%) | 2/2 (100%) | 0/2 (0%) |
| CK 24 h | CON/PBO vs CRY | 4 | 0/4 (0%) | 1/4 (25%) | 0/4 (0%) | 0/4 (0%) |
| CK 24 h | CON/PBO vs PHO | 4 | 0/4 (0%) | 3/4 (75%) | 0/4 (0%) | 0/4 (0%) |
| CK 24 h | CON/PBO vs STR | 4 | 2/4 (50%) | 4/4 (100%) | 0/4 (0%) | 0/4 (0%) |
| CK 24 h | COMP vs CON/PBO | 3 | 2/3 (67%) | 2/3 (67%) | 2/3 (67%) | 2/3 (67%) |
| CK 24 h | CON/PBO vs CWI | 3 | 3/3 (100%) | 3/3 (100%) | 2/3 (67%) | 2/3 (67%) |
| CK 24 h | CON/PBO vs VIB | 3 | 3/3 (100%) | 3/3 (100%) | 2/3 (67%) | 0/3 (0%) |
| CK 48 h | CON/PBO vs CRY | 5 | 0/5 (0%) | 2/5 (40%) | 0/5 (0%) | 0/5 (0%) |
| CK 48 h | CON/PBO vs PHO | 5 | 1/5 (20%) | 4/5 (80%) | 1/5 (20%) | 1/5 (20%) |
| CK 48 h | CON/PBO vs CWI | 3 | 3/3 (100%) | 3/3 (100%) | 2/3 (67%) | 2/3 (67%) |
| CK 48 h | CON/PBO vs STR | 3 | 1/3 (33%) | 3/3 (100%) | 0/3 (0%) | 0/3 (0%) |
| CK 48 h | CON/PBO vs VIB | 3 | 3/3 (100%) | 3/3 (100%) | 2/3 (67%) | 0/3 (0%) |
| CK 48 h | COMP vs CON/PBO | 2 | 1/2 (50%) | 1/2 (50%) | 1/2 (50%) | 1/2 (50%) |
| CK 48 h | CON/PBO vs KT | 2 | 1/2 (50%) | 2/2 (100%) | 0/2 (0%) | 0/2 (0%) |
| CK 48 h | CON/PBO vs LLLT | 2 | 0/2 (0%) | 2/2 (100%) | 1/2 (50%) | 0/2 (0%) |
| CK 48 h | CRY vs PHO | 2 | 0/2 (0%) | 1/2 (50%) | 0/2 (0%) | 0/2 (0%) |
| IL-6 24 h | CON/PBO vs VIB | 3 | 3/3 (100%) | 3/3 (100%) | 2/3 (67%) | 0/3 (0%) |
| IL-6 24 h | COMP vs CON/PBO | 2 | 2/2 (100%) | 2/2 (100%) | 1/2 (50%) | 1/2 (50%) |
| IL-6 48 h | CON/PBO vs VIB | 3 | 3/3 (100%) | 3/3 (100%) | 2/3 (67%) | 0/3 (0%) |
| IL-6 48 h | COMP vs CON/PBO | 2 | 2/2 (100%) | 2/2 (100%) | 1/2 (50%) | 1/2 (50%) |

Note. Values represent the number/proportion of studies within each direct comparison classified as category 1 according to the coding rules in Supplementary Table 27. Only direct comparisons supported by at least two studies are presented because a meaningful distribution of effect modifiers cannot be evaluated empirically for single-study contrasts. InductionType: systemic/whole-body/high-intensity versus localized eccentric/isokinetic model; MuscleRegion: lower-limb/whole-body/systemic versus upper/local region; TrainingStatus: trained/athletic versus untrained/healthy/recreational participants; Design: randomized crossover versus parallel-group trial.

11. Control-condition classification and sensitivity analyses

To examine the influence of pooling different inactive comparators, control arms were reclassified from the original reports as passive/no-treatment/usual-recovery control (CON), placebo/sham control (PBO_SHAM), or unclear. Across the 35 included studies, 19 were classified as CON, 14 as PBO_SHAM, and 2 as unclear. The original comparator description and classification are summarized in Supplementary Table 37. In the control-split sensitivity analyses, CON and PBO_SHAM were treated as separate nodes. Studies with an unclear comparator were excluded from the relevant DOMS or CK sensitivity network. The same Bayesian random-effects model, priors, MCMC settings, and convergence criteria as in the primary analyses were used.

Supplementary Table 37. Classification of control conditions in the included studies.

| **Study** | **Design** | **Original comparator description** | **Classification** | **Outcome/time point(s)** |
| --- | --- | --- | --- | --- |
| Antonialli2014 | parallel RCT | group D: 0 J in each site (placebo) | PBO_SHAM | DOMS 24 h,48 h; CK 24 h,48 h |
| Boobphachart 2017 | parallel RCT | placebo tape group | PBO_SHAM | CK 24 h,48 h |
| Cheng 2022 | parallel RCT | control group (CG, 0 Hz) | PBO_SHAM | DOMS 24 h,48 h; CK 24 h,48 h; IL-6 24 h,48 h |
| Cheng 2025 | parallel RCT | control group with vibration platform closed/turned off | PBO_SHAM | DOMS 24 h,48 h; CK 24 h,48 h; IL-6 24 h,48 h |
| De Marchi 2017 | parallel RCT | placebo group; 0 J placebo PBMT | PBO_SHAM | CK 24 h,48 h |
| Duffield 2010 | crossover RCT | without lower-body compression garments | CON | CK 24 h |
| Fonseca 2016 | crossover RCT | control condition (passive recovery) | CON | CK 24 h,48 h |
| Guilhem 2013 | parallel RCT | control group (CONT; passive recovery) | CON | DOMS 24 h,48 h; CK 24 h,48 h; IL-6 24 h,48 h |
| Kim 2017 | parallel RCT | control group (CON), content not described | UNCLEAR | DOMS 24 h,48 h; CK 24 h,48 h |
| Machado 2020 | parallel RCT | placebo PBMT-sMF program | PBO_SHAM | DOMS 24 h,48 h; CK 24 h,48 h |
| Oakley 2013 | parallel RCT | control group; no intervention | CON | CK 24 h,48 h |
| Pruscino 2013 | crossover RCT | loose pants (CON) | CON | CK 24 h,48 h; IL-6 24 h,48 h |
| Qu 2020 | crossover RCT | control group; seated recovery | CON | DOMS 24 h,48 h; CK 24 h,48 h |
| Eston 1999 | parallel RCT | control group (no treatment) | CON | CK 24 h,48 h |
| Torres 2013 | parallel RCT | eccentric exercise only (ECCG) | CON | DOMS 24 h,48 h; CK 24 h,48 h |
| Wei 2025 | parallel RCT | Group F (Control): no intervention | CON | CK 24 h,48 h; IL-6 24 h,48 h |
| de Paiva 2016 | parallel RCT | placebo PBMT devices | PBO_SHAM | DOMS 24 h,48 h; CK 24 h,48 h |
| Hazar Kanik 2019 | parallel RCT | placebo KT group | PBO_SHAM | DOMS 48 h; CK 48 h |
| Douris 2006 | parallel RCT | both no-treatment and sham groups; pooled row not uniquely traceable | UNCLEAR | DOMS 24 h,48 h |
| Fleckenstein 2016 | parallel RCT | no treatment (CON) | CON | DOMS 24 h,48 h |
| Gatterer 2015 | crossover RCT | sham treatment | PBO_SHAM | DOMS 24 h,48 h |
| Itoh 2008 | parallel RCT | control group; no treatment | CON | DOMS 24 h,48 h |
| Kirmizigil 2020 | crossover RCT | non-KT control trial | CON | DOMS 24 h,48 h |
| Lee 2015 | parallel RCT | control; no treatment | CON | DOMS 24 h,48 h |
| McLoughlin 2004 | crossover RCT | electrodes with no electrical current | PBO_SHAM | DOMS 24 h,48 h |
| Mikhail Santos Cerqueira 2015 | parallel RCT | control; no intervention | CON | DOMS 24 h,48 h |
| Szymura 2016 | crossover RCT | passive recovery/passive rest | CON | DOMS 24 h,48 h |
| Xue 2023 | parallel RCT | control; no intervention except DOMS modelling | CON | DOMS 24 h,48 h; IL-6 24 h,48 h |
| Malte Krueger 2019 | crossover RCT | passive control condition (CON) | CON | IL-6 24 h |
| Kay 2018 | parallel RCT | control continued normal physical activities | CON | CK 24 h |
| Vanin 2016 | parallel RCT | 0 J placebo LLLT | PBO_SHAM | DOMS 24 h,48 h; CK 24 h,48 h; IL-6 24 h,48 h |
| Xia 2022 | parallel RCT | sham LIFU without energy output | PBO_SHAM | DOMS 24 h,48 h; CK 24 h; IL-6 24 h |
| Sudhakar 2023 | parallel RCT | Group C received no treatment | CON | CK 24 h,48 h |
| Pinto 2022 | crossover RCT | placebo PBMT-sMF mode | PBO_SHAM | CK 48 h; IL-6 24 h,48 h |
| Baroni 2010 | parallel RCT | placebo treatment; device turned off | PBO_SHAM | DOMS 24 h,48 h; CK 48 h |

Note. CON includes passive recovery, usual recovery, no treatment, or no active recovery intervention. PBO_SHAM includes placebo/sham devices, sham stimulation, placebo taping, or other procedures intended to mimic the active intervention without the therapeutic component. Douris 2006 and Kim 2017 were classified as unclear because the comparator used for the pooled NMA row could not be uniquely determined from the available report. Hazar Kanik was published in 2019.

Control-split sensitivity analyses were feasible for DOMS and CK at both 24 h and 48 h. For DOMS, Douris 2006 and Kim 2017 were excluded because of unclear control classification; for CK, Kim 2017 was excluded. All four sensitivity models showed satisfactory MCMC convergence. For IL-6, separating CON from PBO_SHAM disconnected the already sparse networks, so a valid control-split NMA was not estimable. Model-fit and heterogeneity summaries are reported in Supplementary Table 38, and the principal treatment-effect comparisons are shown in Supplementary Table 39.

Supplementary Table 38. Model fit and heterogeneity for control-split sensitivity analyses.

| **Outcome/time** | **Studies** | **Arms** | **Dbar** | **pD** | **DIC** | **Between-study SD (95% CrI)** |
| --- | --- | --- | --- | --- | --- | --- |
| DOMS 24 h | 20 | 44 | 43.815 | 42.584 | 86.399 | 14.56 (8.30 to 25.50) |
| DOMS 48 h | 21 | 46 | 46.700 | 44.425 | 91.124 | 13.85 (7.99 to 24.00) |
| CK 24 h | 20 | 48 | 47.702 | 45.614 | 93.317 | 129.18 (76.48 to 213.83) |
| CK 48 h | 20 | 49 | 48.917 | 46.230 | 95.147 | 196.95 (123.60 to 319.51) |
| IL-6 24 h | — | — | — | — | — | Not estimable: split network disconnected |
| IL-6 48 h | — | — | — | — | — | Not estimable: split network disconnected |

Note. CON and PBO_SHAM were treated as separate nodes. The same random-effects Bayesian model and MCMC settings as in the primary analyses were used. DIC values should not be directly compared with the primary analyses as a formal model-selection test because studies with unclear control classification were excluded from the sensitivity datasets.

Supplementary Table 39. Principal treatment effects in the primary and control-split sensitivity analyses.

| **Outcome/time** | **Comparison** | **Primary analysis MD (95% CrI)** | **Control-split MD (95% CrI)** | **Interpretation** |
| --- | --- | --- | --- | --- |
| DOMS 24 h | WBC vs control | −38.92 (−69.08 to −9.15) | −38.98 (−71.28 to −5.95) | Direction and non-null CrI retained |
| DOMS 24 h | LIFU vs control | −34.54 (−62.45 to −6.61) | −38.14 (−91.08 to 15.80) | Negative point estimate retained; CrI included 0 |
| DOMS 24 h | PHO vs control | −18.20 (−32.91 to −3.72) | −25.50 (−69.90 to 17.59) | Negative point estimate retained; CrI included 0 |
| DOMS 24 h | PBO_SHAM vs CON | Not applicable | −3.68 (−47.13 to 40.33) | No clear difference detected; imprecise |
| DOMS 48 h | LIFU vs control | −65.81 (−92.30 to −39.66) | −73.30 (−112.41 to −33.69) | Direction and non-null CrI retained |
| DOMS 48 h | WBC vs control | −40.03 (−67.74 to −12.44) | −39.92 (−69.90 to −9.19) | Direction and non-null CrI retained |
| DOMS 48 h | PHO vs control | −19.46 (−33.55 to −5.99) | −26.19 (−56.23 to 3.21) | Negative point estimate retained; CrI included 0 |
| DOMS 48 h | PBO_SHAM vs CON | Not applicable | −7.22 (−33.70 to 19.18) | No clear difference detected; imprecise |
| CK 24 h | LLLT vs control | −249.70 (−502.23 to −1.08) | −197.25 (−526.24 to 133.53) | Negative point estimate retained; CrI included 0 |
| CK 24 h | PHO vs control | −199.48 (−329.27 to −80.63) | −154.44 (−372.41 to 60.05) | Negative point estimate retained; CrI included 0 |
| CK 24 h | PBO_SHAM vs CON | Not applicable | 52.34 (−131.10 to 244.03) | No clear difference detected; imprecise |
| CK 48 h | LLLT vs control | −499.96 (−831.59 to −203.59) | −524.83 (−1002.45 to −106.10) | Direction and non-null CrI retained |
| CK 48 h | PHO vs control | −252.50 (−420.45 to −87.47) | −270.90 (−606.25 to 41.33) | Negative point estimate retained; CrI included 0 |
| CK 48 h | PBO_SHAM vs CON | Not applicable | −19.24 (−312.11 to 258.22) | No clear difference detected; imprecise |

Note. In the primary analysis, the comparator was the combined CON/PBO node; in the control-split analysis, the active-treatment estimates shown here are relative to CON. Negative MD values favor the active recovery intervention for DOMS and CK. A 95% CrI crossing 0 indicates that the estimate was compatible with no difference. The PBO_SHAM versus CON estimates are presented to assess whether a systematic difference between control types was detectable; wide CrIs preclude claims of equivalence.

[1]	PUHAN M A, SCHüNEMANN H J, MURAD M H, et al. A GRADE Working Group approach for rating the quality of treatment effect estimates from network meta-analysis [J]. BMJ : British Medical Journal, 2014, 349: g5630.

[2]	BRIGNARDELLO-PETERSEN R, BONNER A, ALEXANDER P E, et al. Advances in the GRADE approach to rate the certainty in estimates from a network meta-analysis [J]. J Clin Epidemiol, 2018, 93: 36-44.

[3]	DIAS S, ADES, A. E., WELTON, N. J., JANSEN, J. P. & SUTTON, A. J. Network meta-analysis for decision-making. [J]. 2018.
